# An axis of genetic heterogeneity in autism is indexed by age at diagnosis and is associated with varying developmental and mental health profiles

**DOI:** 10.1101/2024.07.31.24311279

**Published:** 2024-08-02

**Authors:** Xinhe Zhang, Jakob Grove, Yuanjun Gu, Cornelia K. Buus, Lea K. Nielsen, Sharon A.S. Neufeld, Mahmoud Koko, Daniel S Malawsky, Emma Wade, Ellen Verhoef, Anna Gui, Laura Hegemann, APEX consortium, iPSYCH Autism Consortium, PGC-PTSD Consortium, Daniel H. Geschwind, Naomi R. Wray, Alexandra Havdahl, Angelica Ronald, Beate St. Pourcain, Elise B. Robinson, Thomas Bourgeron, Simon Baron-Cohen, Anders D. Børglum, Hilary C. Martin, Varun Warrier

**Affiliations:** 1 Department of Psychiatry, University of Cambridge; 2 Autism Research Centre, Department of Psychiatry, University of Cambridge, Cambridge, UK; 3 The Lundbeck Foundation Initiative for Integrative Psychiatric Research, iPSYCH, Aarhus, Denmark; 4 Center for Genomics and Personalized Medicine (CGPM), Aarhus University, Aarhus, Denmark; 5 Department of Biomedicine (Human Genetics) and iSEQ Center, Aarhus University, Aarhus, Denmark; 6 Bioinformatics Research Centre, Aarhus University, Aarhus, Denmark; 7 Human Genetics Programme, Wellcome Sanger Institute, Wellcome Genome Campus, Hinxton, CB10 1SA, UK; 8 Language and Genetics Department, Max Planck Institute for Psycholinguistics, Nijmegen, The Netherlands; 9 Department of Psychology, University of Essex, Wivenhoe Park, Colchester, CO4 3SQ, United Kingdom; 10 Centre for Brain and Cognitive Development, Department of Psychological Sciences, Birkbeck University of London, London, WC1E 7HX, United Kingdom; 11 Department of Psychology, University of Oslo, Oslo, Norway; 12 Nic Waals Institute, Lovisenberg Diaconal Hospital, Oslo, Norway; 13 PsychGen Center for Genetic Epidemiology and Mental Health, Norwegian Institute of Public Health, Oslo, Norway; 14 Program in Neurobehavioral Genetics and Center for Autism Research and Treatment, Semel Institute, David Geffen School of Medicine, University of California, Los Angeles, Los Angeles, CA 90095, USA.; 15 Program in Neurogenetics, Department of Neurology, David Geffen School of Medicine, University of California, Los Angeles, Los Angeles, CA 90095, USA.; 16 Department of Psychiatry, Semel Institute, David Geffen School of Medicine, University of California, Los Angeles, Los Angeles, CA 90095, USA.; 17 Department of Human Genetics, David Geffen School of Medicine, University of California, Los Angeles, Los Angeles, CA 90095, USA.; 18 Institute for Molecular Bioscience, University of Queensland, Brisbane, QLD, Australia; 19 Department of Psychiatry, University of Oxford, Oxford, UK; 20 School of Psychology, University of Surrey, Guildford, Surrey, GU2 7XH, United Kingdom; 21 MRC Integrative Epidemiology Unit, University of Bristol, United Kingdom.; 22 Donders Institute for Brain, Cognition and Behaviour, Radboud University, The Netherlands.; 23 Center for Genomic Medicine, Massachusetts General Hospital, Boston, MA, USA; 24 The Broad Institute of MIT and Harvard, Cambridge, MA, USA; 25 Human Genetics and Cognitive Functions, Institut Pasteur, UMR3571 CNRS, IUF, Université Paris Cité, Paris, France; 26 Department of Psychology, University of Cambridge, Cambridge, UK

## Abstract

There is growing recognition that earliest signs of autism need not clearly manifest in the first three years of life. To what extent is this variation in developmental trajectories associated with age at autism diagnosis? Does the genetic profile of autism vary with age at autism diagnosis? Using longitudinal data from four birth cohorts, we demonstrate that two different trajectories of socio-emotional behaviours are associated with age at diagnosis. We further demonstrate that the age at autism diagnosis is partly heritable (h^2^_SNP_ = 0.12, s.e.m = 0.01), and is associated with two moderately correlated (r_g_ = 0.38, s.e.m = 0.07) autism polygenic factors. One of these factors is associated with earlier diagnosis of autism, lower social and communication abilities in early childhood. The second factor is associated with later autism diagnosis, increased socio-emotional difficulties in adolescence, and has moderate to high positive genetic correlations with Attention-Deficit/Hyperactivity Disorder, mental health conditions, and trauma. Overall, our research identifies an axis of heterogeneity in autism, indexed by age at diagnosis, which partly explains heterogeneity in autism and the profiles of co-occurring neurodevelopmental and mental health profiles. Our findings have important implications for how we conceptualise autism and provide one model to explain some of the diversity within autism.

Autism is a term used to describe a group of conditions characterised by difficulties in social-communication, unusually restricted and repetitive interests, and sensory differences^1^. Ever since its earliest descriptions in the 1940s^2,3^, autism has been thought of as a condition that typically emerges and is diagnosed in early childhood. However, recent studies demonstrate that more autistic individuals are now receiving an autism diagnosis from mid-childhood onwards than in early childhood^4–6^.

One factor that may explain these findings is a shift in the conceptualisation of autism over time. There is a growing recognition that the signs of autism may not clearly manifest in the first three years of life^1,7–9^, which has been recognised by the changes to the diagnostic criteria for autism by DSM-5 and ICD-11. Supporting this, several studies have demonstrated that a subset of children who do not initially meet the criteria for an autism diagnosis later receive a diagnosis^7,10–14^

These findings pose a series of fundamental questions regarding the aetiology of autism. For instance, given the substantial heritability of developmental phenotypes^15–17^, to what extent does the genetic profile of autism vary with age at receiving an autism diagnosis? How does the developmental variation in the emergence of autism features contribute to age at autism diagnosis, and consequently, the genetic heterogeneity within autism? Is the higher prevalence of mental health diagnosis among autistic individuals diagnosed later in life^18,19^ partly due to genetic factors?

We address these questions using multiple epidemiological and genetic datasets. Using longitudinal data from birth cohorts, we demonstrate that individual differences in socio-behavioural trajectories are associated with age at autism diagnosis. We further demonstrate that age at autism diagnosis is heritable, and this heritability can be partly explained by two correlated polygenic autism factors. The two polygenic factors are differentially associated with developmental and mental health profiles, and partly explain the genetic heterogeneity of autism. We provide a summary of the study and address potential questions regarding the implications of the findings in the Supplementary FAQs.

## Socio-behavioural trajectories are linked to varying age at autism diagnosis

**Extended Figure 1:**
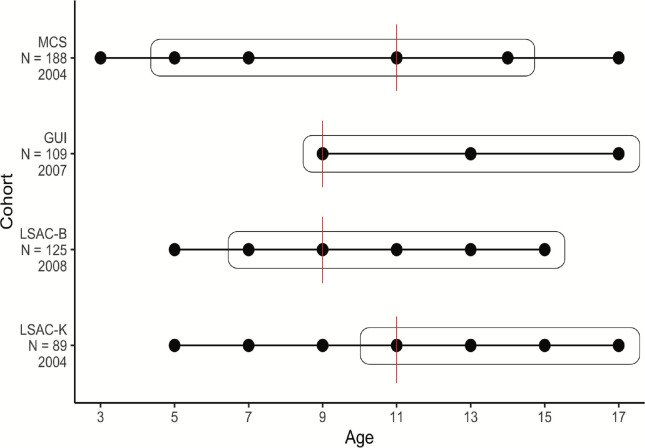
Schematic diagram of the cohorts included in the study and the ages when data was collected for SDQ scores (dots) and autism diagnosis (in boxes). MCS = Millennium Cohort Study; GUI = Growing up in Ireland (cohort ‘98); LSAC-B = Longitudinal Study of Australian Children (Birth cohort); LSAC-K = Longitudinal Study of Australian Children (Kindergarten cohort). Sample sizes and the year of initial SDQ data collection for each cohort are shown on the ordinate axis. The age cutoff used in the Latent Growth Curve Models for each cohort is indicated by a red line.

We first investigated the association between variable developmental trajectories and age at autism diagnosis using four birth cohorts. This included Growing Up in Ireland (GUI, born in 1998), Millennium Cohort Study (MCS, 2000), and Longitudinal Study of Australian Children: Kindergarten cohort (LSAC-K, 1999) and Birth cohort (LSAC-B, 2003) (Supplementary Table 1, Extended Figure 1, Supplementary Note 1). All four cohorts collected longitudinal information on socio-behaviour measured using the caregiver-reported Strengths and Difficulties Questionnaire (SDQ)^20^ and its subscales, and autism diagnosis in data collection sweeps (hereafter “sweeps”) at different ages. The SDQ is widely used, has excellent psychometric properties^21–23^, and is largely invariant across age, sex, and different populations^24–26^, meaning that it is measuring the same latent trait across these demographic variables.

Given increasing number of autistic individuals being diagnosed in adolescence^4,5^, we wondered if there are broad differences in the trajectories of SDQ total and subscale scores among autistic individuals diagnosed before the ages of 9 – 11 (childhood diagnosed group, N = 39 – 118 across cohorts, Supplementary Table 1) and after (adolescent diagnosed group, N = 27 −73 across cohorts) (Methods). This age cutoff period corresponds to the onset of puberty, the transition from primary to secondary school, and the beginning of an increase of incidence in diagnosis of autism in girls^27,28,29^. The specific cutoff age was cohort-dependent, as different birth cohorts collected information on autism diagnosis at different ages.

**Figure 1:**
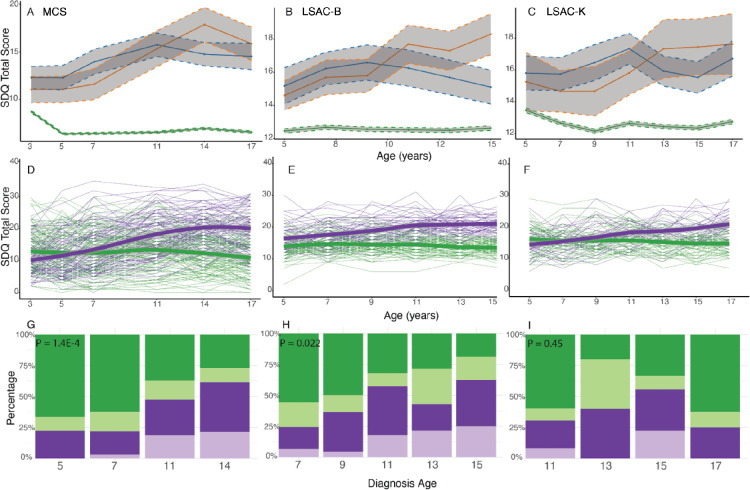
Trajectory analyses in three of the four birth cohorts. A - C: Mean SDQ total scores in autistic individuals diagnosed in childhood (blue) and adolescence (orange), and individuals without an autism diagnosis (green) in the MCS (A), LSAC-B (B), and LSAC-K (C) cohorts. Grey regions indicate 95% confidence intervals. D - F: Longitudinal growth mixture models of SDQ total scores among autistic individuals, demonstrating the presence of two groups (green indicating early childhood emergent latent class and purple indicating late childhood emergent latent class) in the MCS (D), LSAC-B (E) and LSAC-K (F) cohorts. G - I: Stacked bar charts providing the proportion of individuals who had been diagnosed as autistic at specific ages, categorised by membership in the latent classes identified from the growth mixture models in MCS (G), LSAC-B (H), and LSAC-K (I) cohorts. Darker colours indicate males and lighter colours indicate females. P-values (inset) are from chi-square tests comparing the distribution of age at autism diagnosis between the two latent classes. Results from GUI have not been plotted here and are available in Supplementary Figure 6.

We used Latent Growth Curve Models to linearly model the trajectories of SDQ total score and subscales in all four cohorts for both childhood and adolescent diagnosed groups. Across four cohorts, Latent Growth Curve Models identified different trajectories of SDQ total scores between the childhood and adolescent diagnosed groups (Mean scores in Figure 1A–C, Supplementary Figures 1 - 6, Supplementary Table 2). Similar results were obtained for peer relationship problems and prosocial behaviours in all four cohorts (Supplementary Figures 4 and 5). Compared with individuals without an autism diagnosis at any time point, the childhood diagnosed autistic group had higher difficulties in early childhood that remained relatively stable or gently declined in adolescence. Compared to the childhood diagnosed autistic group, the adolescent diagnosed autistic group had fewer difficulties during early childhood, but difficulties increased in later childhood and persisted into adolescence.

In MCS, we ran a series of sensitivity analyses to check the robustness of the above results. We obtained consistent results: (1) in an expanded sample of MCS which included autistic individuals with co-occurring Attention-Deficit/Hyperactivity Disorder (ADHD) and inconsistent reports of an autism diagnosis (MCS-expanded, Methods, Supplementary Table 2); (2) after imputing missing SDQ scores (MCS-imputed) (Supplementary Table 3, Supplementary Note 2); and (3) when restricting to males. In all four birth cohorts, models stratified by age at diagnosis were generally a better fit to the data than sex-stratifed models (Supplementary Table 2), suggesting that the results do not primarily reflect sex differences in age at autism diagnosis (Supplementary Note 3).

To assess the specificity for autism, we ran Latent Growth Curve Models on SDQ total scores and subscales with children with an ADHD but without an autism diagnosis in the MCS cohort (N = 89). Children with ADHD diagnosed in childhood and adolescence differed only nominally in the slopes of the hyperactivity/inattention (P = 0.026) and prosocial behaviour subscales (P = 0.029) (Supplementary Table 4 and Supplementary Figures 7 and 8). In MCS, compared to adolescent diagnosed children with ADHD, adolescent diagnosed autistic children had a steeper increase in peer relationship problems (P = 5.77×10^−3^) and emotional symptoms (P = 0.012) across development. However, these results must be interpreted cautiously given the low number of children with only ADHD in MCS.

Recognising that the age at diagnosis threshold used to categorise autistic individuals into two groups is to some extent arbitrary, we used Growth Mixture Models (GMMs) to identify latent trajectories of SDQ total and subscale scores among autistic individuals in all four cohorts. GMMs do not require *a priori* grouping but identify subgroups based on longitudinal changes in SDQ scores.

Across three of the four birth cohorts, GMMs identified two-trajectory models as being optimal for SDQ total scores and a majority of subscale scores (Supplementary Table 5). The exception was the GUI cohort, where a one-trajectory model was optimal, likely due to fewer sweeps (three) for SDQ scores. Amongst the other cohorts, one latent trajectory was characterised by difficulties in early childhood, which remained stable or gently declined with age (early childhood emergent latent class). The other latent trajectory was characterised by fewer difficulties in early childhood which increased in late childhood and adolescence (late childhood emergent latent class) (Figure 1 D–F). Autistic individuals in the early childhood emergent latent class were more likely to be diagnosed in childhood compared to autistic individuals in the late childhood emergent latent class in MCS (P = 1.43×10^−4^, chi-square test) and LSAC-B (P = 0.022, chi-square test) (Figure 1 G–I, Supplementary Table 6), but this difference was not significant in LSAC-K, possibly because age 11 was the earliest when an autism diagnosis was recorded.

Similar results were obtained for all the SDQ subscales in MCS, LSAC-B and LSAC-K except for conduct and peer relationship problems in LSAC-K, where no distinct trajectory groups were identified (Supplementary Table 5, Supplementary Figures 1 - 5). In MCS, these results were largely consistent: (1) in the expanded sample after including individuals with co-occurring ADHD and inconsistent autism diagnoses (Supplementary Table 4); (2) after imputation (Supplementary Table 3, Supplementary Note 4); and (3) when restricting to only males (Supplementary Table 5). In contrast, although we obtained two latent classes based on SDQ and subscale trajectories among individuals with a diagnosis of ADHD but not autism, these latent classes were not significantly associated with age at ADHD diagnosis (Supplementary Table 4, Supplementary Figures 7 and 8).

In all three cohorts, sex ratio in the late childhood emergent latent class of SDQ total scores compared to the early childhood emergent class was statistically similar (male: female ratio = 3.92 – 2.11; P > 0.05, chi-square tests, Supplementary Table 6). After accounting for sex, individuals in the late childhood emergent latent class were more likely than those in the early childhood emergent class to have higher depressive symptoms measured using the Short Mood and Feelings Questionnaire^30^ (MCS-C, P = 3.84×10^−4^; LSAC-B, P = 9.82×10^−13^; LSAC-K, P = 1.86×10^−6^), have higher rates of diagnosed anxiety (LSAC-B, P = 5.54×10^−7^; LSAC-K, P = 3.99×10^−3^) and depression (LSAC-B, P = 3.67×10^−4^; LSAC-K, P = 4.69×10^−3^), were more likely to self-harm (LSAC-B, P = 0.018), or have higher rates of suicidal ideation (MCS, P = 2.50×10^−3^) (Supplementary Table 7).

Given the significant association between age at autism diagnosis and the GMM latent class membership in LSAC-B and MCS, we wondered if these socio-behavioural trajectories explain any variance in age at autism diagnosis after accounting for socio-demographic factors (e.g., sex, ethnic minority, SES, living area deprivation) and child’s cognitive aptitude, all associated with age at autism diagnosis^31,32^. Multiple linear regression models indicated that latent class membership from the GMM, socio-demographic factors, and child’s cognitive aptitude together accounted for 17.4% (LSAC-B) - 35.0% (MCS) of the variance in age at autism diagnosis (Supplementary Table 8). Latent classes of SDQ total and subscale scores alone explained 9.9% (LSAC-B) - 30.3% (MCS) of total variance. In MCS-expanded, the full model explained 14.8% of the variance, and SDQ total and subscale scores latent class memberships explained 10.0% of the variance. In MCS-imputed, the full model explained 59.8% of the variance and the latent class memberships of SDQ total and subscale scores explained 56.6% of the variance (Supplementary Table 3).

We further investigated if the effects of socio-demographic factors and cognitive aptitude on age at diagnosis were partly mediated by SDQ latent classes, but did not identify a significant mediated effect. This suggests that the effects of the demographic variables on age at autism diagnosis is largely independent of the effects of SDQ latent classes (Supplementary Table 8, Supplementary Note 5).

## Age at autism diagnosis is partly genetic

The above analyses demonstrate that variation in socio-behavioural trajectories, measured using the SDQ, is associated with age at autism diagnosis. Previous research has demonstrated that developmental variation in socio-behavioural profiles is partly explained by genetic factors^33–38^. A corollary of this is that genetic factors may also be associated with age at autism diagnosis.

**Figure 2:**
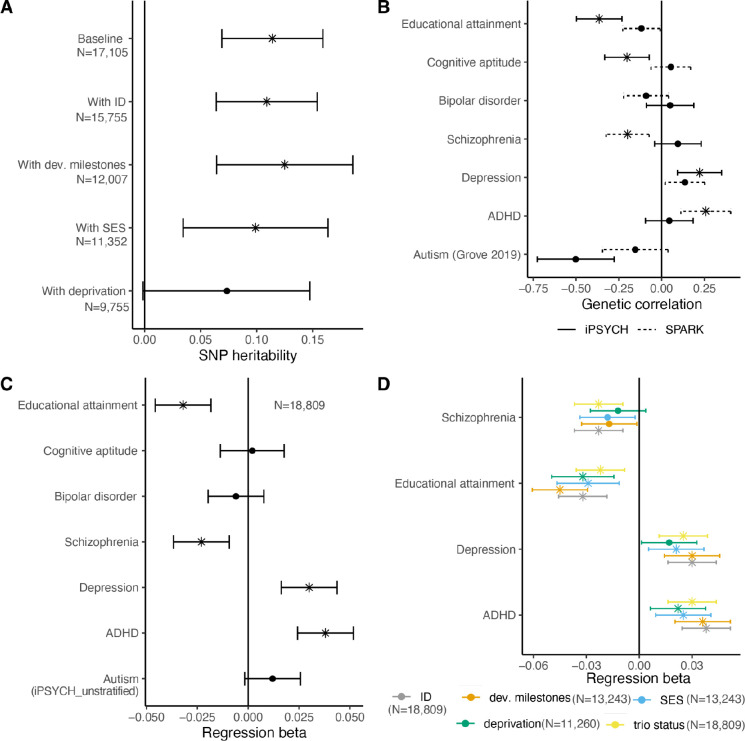
Genetic correlates of age at autism diagnosis A. SNP-based heritability of age at autism diagnosis with and without the inclusion of covariates. B. Genetic correlation between age at autism diagnosis genome-wide association studies (GWAS) from SPARK and iPSYCH and psychiatric, neurodevelopmental, and cognitive phenotypes. The Autism GWAS used is the Grove et al., 2019 GWAS. C. Association between PGS for selected neurodevelopmental, cognitive and psychiatric phenotypes and age at autism diagnosis in SPARK. Estimates provided after correcting for ID, sex, age at recruitment into the study, and 10 genetic principal components. Sample sizes provided in inset. D. For those significant in C, association of PGS for schizophrenia, educational attainment, depression, cognitive aptitude and ADHD with age at autism diagnosis in baseline models and after correcting for intellectual disability (ID), developmental (dev.) milestones, socio-economic status (SES) and deprivation. Sample sizes provided in parenthesis. For all plots, points indicate the estimate, whiskers indicate 95% confidence intervals. For plots B-D points with an asterisk (*) indicate significant associations with Benjamini-Yekutieli (BY) adjustment. For plot A, an asterisk (*) indicates significance at P < 0.05 as no multiple testing adjustment is needed for the sensitivity analyses, and whiskers indicate 95% confidence intervals.

We tested this in a US-based cohort of autistic individuals (SPARK^39^ : N_max_ = 17,105) where we identified a significant heritability estimated from single nucleotide polymorphisms (SNP-based heritability) for age at autism diagnosis (h^2^_SNP_ = 0.11, s.e.m = 0.01). This heritability did not significantly attenuate after accounting for the child’s developmental phenotypes (age of walking, age at first words, and intellectual disability [ID]), parental socio-economic status that may correlate with greater parental awareness and ability to access diagnostic services, and neighbourhood socioeconomic deprivation which may index availability of healthcare services (Figure 2A, Supplementary Table 9).

We used genetic correlation and polygenic score (PGS) analyses to better characterise the genetics of age at autism diagnosis (N_max_ = 18,809). Later age at autism diagnosis was significantly positively genetically correlated with ADHD^40^, and negatively with schizophrenia^41^ (Figure 2B and 2C, Supplementary Table 10). In SPARK, PGS for ADHD and educational attainment remained significantly associated with age at diagnosis after accounting for ID, developmental milestones, socio-economic status and neighbourhood deprivation, and trio status (i.,e, two parents and one child, Figure 2D, Supplementary Table 11).

As females are significantly more likely to be diagnosed as autistic later than males^27^, we investigated if there is an interaction effect between sex and the PGS for four phenotypes significantly associated with age at autism diagnosis. We did not identify any significant PGS-by-sex interaction effects (Supplementary Table 12).

Parents play an important role in recognising autistic features in their children and pursuing an autism diagnosis. Consequently, we wondered if the associations between ADHD and educational attainment (EA) PGS on age at autism diagnosis were due to parental indirect genetic effects (where parental genetics impacts child’s age at autism diagnosis via parental behaviours) or child’s direct genetic effects. In 6,554 trios we observed significant direct effects of ADHD (P = 0.015) and significant indirect effects of EA PGS (P = 6.54×10^−4^) on increasing age at autism diagnosis respectively (Supplementary Table 13). These indirect effects must be interpreted cautiously as they are not immune to confounding, including participation bias.

Previous research has demonstrated that autistic individuals are enriched for *de novo* and rare inherited protein truncating or missense variants in genes intolerant to loss of function mutations (constrained genes)^42,43^. Using trios (N = 6,206), we investigated if rare *de novo* or inherited protein truncating variants or missense variants in highly constrained genes were associated with age at autism diagnosis. We found no significant associations with age at autism diagnosis for either type of variants (Supplementary Table 11). This may possibly reflect later autism diagnosis in some carriers of *de novo* mutations due to diagnostic overshadowing by co-occurring ID or global developmental delay.

**Extended Figure 2:**
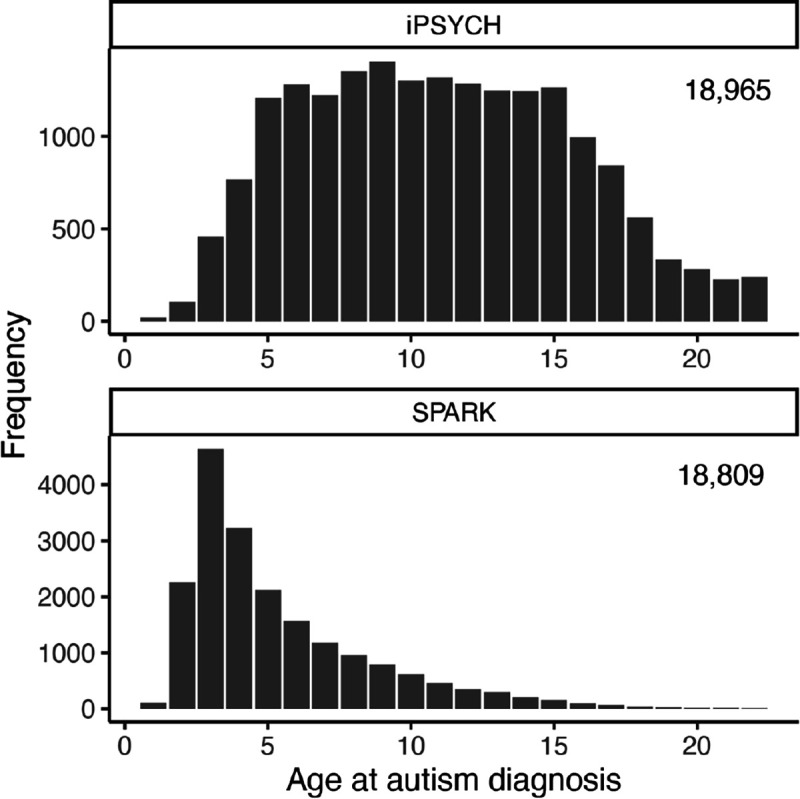
Distribution of age at autism diagnosis in SPARK and iPSYCH. Frequency histograms of age at autism diagnosis in iPSYCH and SPARK. Sample sizes have been provided in the inset.

We examined the generalisability of our findings in the Danish iPSYCH cohort (18,965 autistic individuals, mean diagnosis age 10.98 years, SD = 4.64; compared to SPARK’s 4.97 years, SD = 3.28, Extended Figure 2). A GWAS of age at autism diagnosis in iPSYCH (N = 19,931) showed similar LDSC-based SNP heritability (h^2^_SNP_ = 0.10, s.e.m = 0.03) to SPARK (h^2^_SNP_ = 0.09, s.e.m = 0.03) and moderate genetic correlation between cohorts (r_g_ = 0.51, s.e.m = 0.19, P = 7.56×10^−3^). The iPSYCH GWAS had positive genetic correlation with depression^44^ and negative correlation with educational attainment^45^ and cognitive aptitude^46^. Differences in genetic correlations may be due to varying age distributions and potential participation bias in SPARK.

## Characterising the genetic relationship between age at autism diagnosis and autism

The previous findings collectively demonstrate that age at autism diagnosis is heritable but with complex genetic correlations that vary by cohort. Subsequently, we sought to characterise the genetic relationship between age at autism diagnosis and autism.

**Figure 3:**
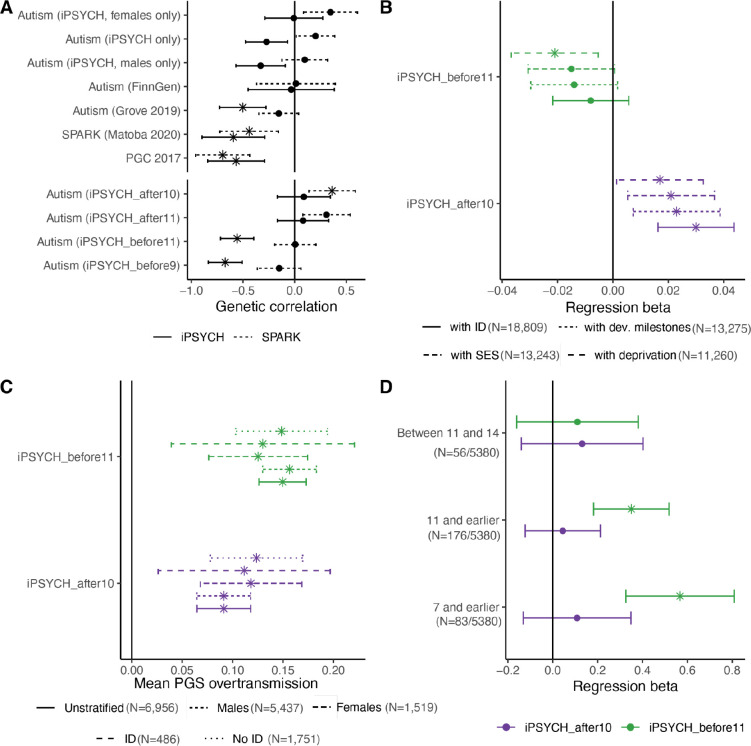
Genetic correlates of age at diagnosis stratified autism GWAS. A. Genetic correlation between age at autism diagnosis in SPARK and different autism GWAS. Sample sizes are provided in Supplementary Table 14. B. Association between age at autism diagnosis PGS for iPSYCH_before11_ and iPSYCH_after10_ in the SPARK cohort. Estimates provided after correcting for ID, developmental (dev.) milestones, socio-economic status (SES) and deprivation. C. Over-transmission of PGS for iPSYCH_before11_ and iPSYCH_after10_ from parents to autistic children in the SPARK cohort. Estimates provided for unstratified and sex-stratified analyses. Children’s PGS have been standardised to parental mean PGS, with the line at zero indicating no over-transmission. D. Association between autism diagnosed in childhood and adolescence and PGS for iPSYCH_befoere 11_and iPSYCH_after10_ GWAS in the MCS cohort. For all plots, points indicate the estimate, whiskers indicate 95% confidence intervals. For graphs A, C, and D, points with an asterisk (*) indicate significant associations with Benjamini-Yekutieli adjustment. N indicates sample size. For graph B, points with an asterisk (*) indicate significant association after Bonferroni correction within each sensitivity analysis. N indicates sample size. For graph D, sample sizes are provided as N_autistic_/N_nonautistic_.

Across phenotypes such as schizophrenia^47^ and depression^48^, the age at diagnosis/onset is largely negatively genetically correlated with the phenotype itself^49^. This indicates that earlier diagnosis/onset is associated with greater polygenic propensity for the condition. However, with autism, we observed variable genetic correlations between age at autism diagnosis and different GWAS of autism, including a nominally significant positive genetic correlation with the females-only iPSYCH^50^ autism GWAS and the SPARK age at autism diagnosis GWAS (Figure 3A, Supplementary Table 14). In addition, both age of diagnosis GWAS had moderate negative genetic correlations with both the Psychiatric Genomics Consortium 2017 (PGC-2017) autism GWAS^51^ and a GWAS of autism in SPARK^52^, consistent with the average age at autism diagnosis in PGC-2017 and SPARK being lower than that of iPSYCH. The pattern of genetic correlations between age at autism diagnosis and the various autism GWAS does not align well with differences in sex-ratio among the GWAS (Supplementary Figure 9), but does align reasonably well with the median age at diagnosis for the autism GWAS.

Given these varying genetic correlations, we wondered if the polygenic signal for age at autism diagnosis reflects a mixture of different age-dependent polygenic traits. To test this, we conducted GWAS of autism within the iPSYCH dataset, stratifying participants into two groups: those diagnosed before age 11 (iPSYCH_before11_, N_autistic_ = 9,500) and those diagnosed at age 11 or later (iPSYCH_after10_, N_autistic_ = 9,231). This roughly coincided with the age window of 9 – 11 where we find an increase in SDQ difficulties in the adolescent diagnosed group. We identified moderate positive genetic correlation (r_g_ = 0.70, s.e.m = 0.06) between the two GWAS, which was significantly less than 1 (P = 3.68×10^−7^). To provide further resolution based on age at diagnosis, we also generated two additional, smaller GWAS of autism stratified by age at diagnosis in iPSYCH: autism diagnosed before age nine (iPSYCH_before9_, N_autistic_ = 5,451) and autism diagnosed after age 11 (iPSYCH_after11_, N_autistic_ = 8,260). We used the same group of unrelated individuals without an autism diagnosis as controls for all four GWAS (N_control_ = 36,667).

Both genetic correlation and PGS association identified positive shared genetics between iPSYCH_after10_ autism and age at autism diagnosis (Figure 3A and B, Supplementary Tables 14 and 15), confirming the validity of the age of diagnosis GWAS. Further sensitivity analyses confirmed that the PGS association could not be explained by: (1) Diagnostic overshadowing due to ADHD; (2) Trio status; or (3) Changes in diagnostic criteria between DSM-IV and DSM-5.

Furthermore, in SPARK, both sets of PGS were over-transmitted from parents to their autistic children with a larger over-transmission of iPSYCH_before11_ compared to iPSYCH_after10_ PGS (P = 1.28×10^−3^) (Supplementary Table 15, Figure 3C). This is consistent with 90% of autistic individuals in SPARK being diagnosed before age 10. We observed consistent results after stratifying by sex and ID (Supplementary Table 15). Finally, in MCS, iPSYCH_before11_ but not iPSYCH_after10_ was associated with autism diagnosed before age 11 (Supplementary Table 16, Figure 3D). Taken together, this suggests that although both sets of PGS are associated with autism, their effects on autism vary by age at diagnosis.

**Extended Figure 3:**
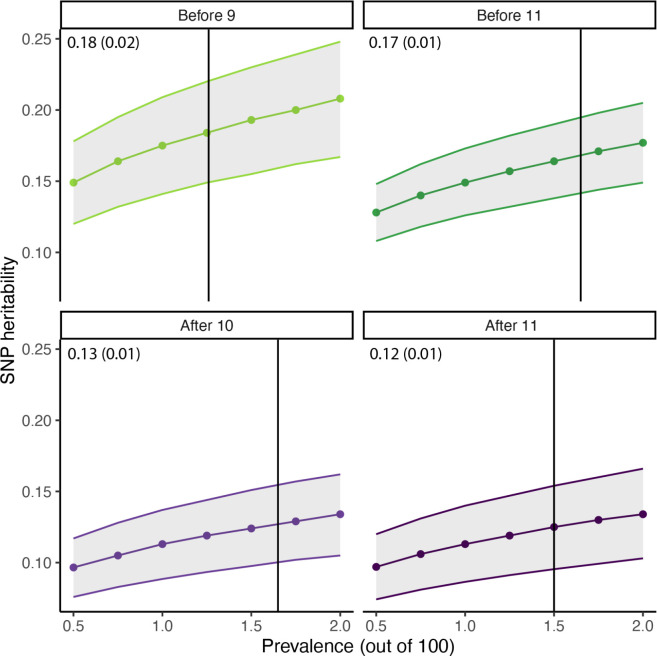
SNP heritability for age at diagnosis stratified autism GWAS. SNP heritability (points) by age at autism diagnosis for varying levels of autism prevalence. Shaded regions, 95% confidence intervals. Each vertical line indicates the best guess autism prevalence for each age at diagnosis stratified autism GWAS. SNP heritability and associated standard error (in parentheses) of autism at the best guess prevalence estimate provided in the top left corner of each facet.

Across a range of prevalence estimates, iPSYCH_before11_ had moderately higher SNP-based heritability (h^2^ = 0.18, s.e.m = 0.02) compared to the iPSYCH_after10_ GWAS (h^2^ = 0.13, s.e.m = 0.01) (Supplementary Table 17, Extended Figure 3). The heritability of iPSYCH_before11_ was statistically similar to SPARK (h^2^ =0.19, s.e.m = 0.03) and PGC-2017 (h^2^ = 0.20, s.e.m = 0.02), suggesting that stratifying by age at diagnosis identifies similar SNP-based heritability between iPSYCH, SPARK, and PGC-2017.

**Extended Figure 4:**
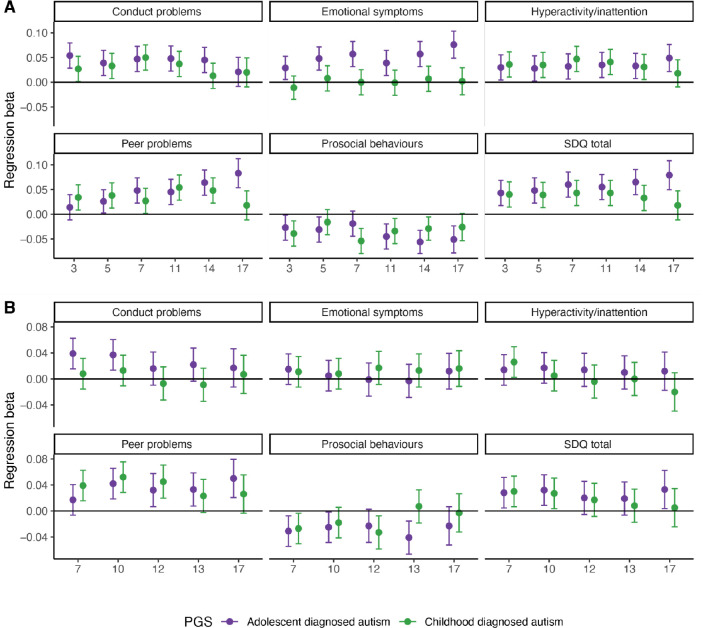
Cross-sectional association between PGS for age at diagnosis stratified autism GWAS and socio-behavioural traits measured at different ages. A. Association between PGS for iPSYCH_after10_ and iPSYCH_before11_ and scores on the SDQ total and subscales in (A) the MCS cohort at six ages (3 – 17) and (B) ALSPAC at five ages (7 – 17). For all plots, points indicate the estimate, whiskers indicate 95% confidence intervals.

We further investigated whether genetics from age-stratified GWAS supported trajectory modelling findings. In the MCS cohort (N = 6,142 – 5,135), multivariate linear mixed effect models with age-by-PGS interaction showed that iPSYCH_after10_ PGS, but not iPSYCH_before11_, was significantly associated with increasing emotional symptoms (BY adjusted P = 7.11×10^−4^), peer relationship problems (BY adjusted P = 1.75×10^−8^), SDQ total scores (BY adjusted P = 2.87×10^−3^), and decreasing prosocial behaviours (FDR adjusted P = 0.030) with age (Supplementary Table 18, Extended Figure 4). Consistent results were obtained when including weights to account for sampling bias (Supplementary Table 18).

In age-by-PGS interaction analyses in ALSPAC (N = 7,172 – 4,977), iPSYCH_before11_ PGS was associated only with decreasing hyperactivity/inattention (BY adjusted P = 4.51×10^−3^), while iPSYCH_after10_ PGS showed nominal associations with increasing peer relationship problems and SDQ total scores with age (Supplementary Table 18). In both cohorts, iPSYCH_after10_ PGS showed larger increases in effect on SDQ total and peer relationship problems scores from childhood to adolescence compared to iPSYCH_before11_ PGS. Entropy balancing did not alter these findings, suggesting that the differences in results are unlikely to be due to ascertainment differences between cohorts, but may instead reflect secular trends in mental health trajectories^53^ or variations in the developmental periods analysed.

## Two correlated autism polygenic factors are associated with differing age at autism diagnosis

The above age at diagnosis stratified analyses suggested that the genetic signal underlying age at autism is likely a mixture of two or more genetic signals, with varying effects on socio-behavioural trajectories. However, any age-based cutoff for diagnosis is inherently arbitrary. Recognising this, we sought to understand the latent genetic structures in autism using different autism GWAS and their relationship with age at autism diagnosis by modelling the genetic covariances among the different autism GWAS.

To enable this and provide greater resolution based on age at diagnosis, we generated three additional age at diagnosis stratified GWAS of autism in SPARK using (unscreened) non-autistic parents and siblings as controls (N_control_ = 24,965). The three GWAS were: SPARK, diagnosed before age 6 (SPARK_before6_; N_autistic_ = 14,578); (2) SPARK, diagnosed before age 11 (SPARK_before11_, N_autistic_ = 18,719); and (3) SPARK, diagnosed after age 10 (SPARK_after10_, N_autistic_ = 3,358).

**Figure 4:**
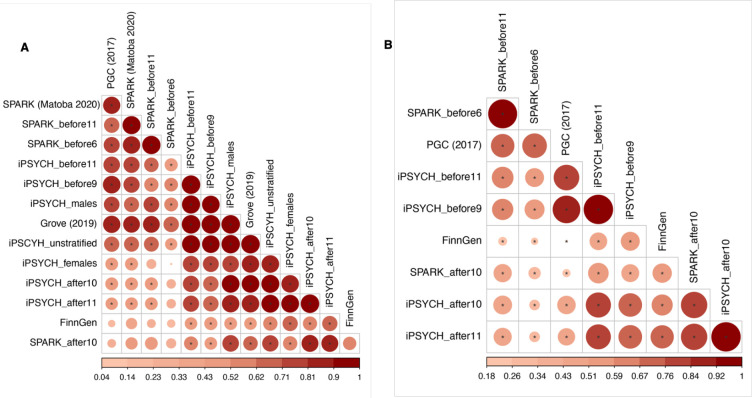
Genetic correlation heatmaps between different GWAS of autism. Genetic correlation heatmaps of (A) all GWAS of autism, and (B) GWAS of autism after excluding GWAS not stratified by age at diagnosis in SPARK and iPSYCH. GWAS have been ordered based on hierarchical clustering of the genetic correlations. Asterisks (*) indicate significant genetic correlations after Benjamini-Yekutieli adjustment.

Next, we generated genetic correlations among all the GWAS of autism we had access to. We observed genetic correlations ranging from 0.04 (s.e.m = 0.14) to 0.98 (s.e.m = 0.01) (Figure 4A, Supplementary Table 19). This was not entirely explained by cohort differences or sex differences (Supplementary Note 3).

Hierarchical clustering of the genetic correlations identified two broad, overlapping clusters (Figure 4A), one comprising GWAS of autism with predominantly childhood diagnosed individuals, and another comprising GWAS of autism with a large fraction of individuals diagnosed in adolescence or later. This pattern became clearer when excluding GWAS not stratified by age at diagnosis in SPARK and iPSYCH (Figure 4B). This is indicative of genetic heterogeneity indexed by age at autism diagnosis.

**Figure 5:**
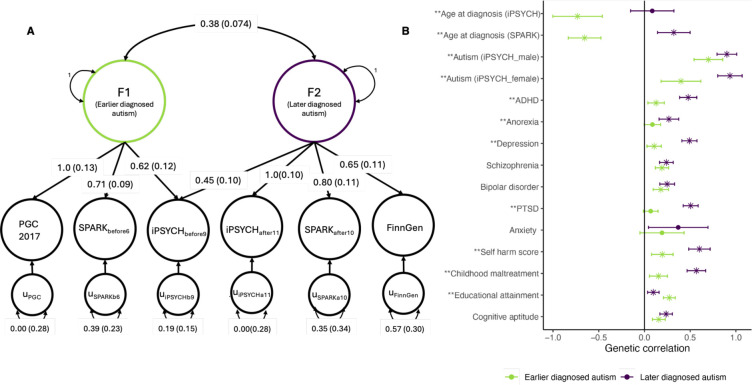
Two genetic latent factors in autism. A. Path diagram illustrating the two-correlated-genetic-factor models for autism, using six minimally overlapping autism GWAS datasets. F1 = Factor 1, F2 = Factor 2. One-headed arrows depict the regression relationship pointing from the independent variables to the dependent variables. The numbers are the regression coefficients of the factor loadings, with the standard errors provided in parentheses. Covariance between variables are represented as two-headed arrows linking the variables. The numbers on the two-headed arrows can be interpreted as genetic correlation estimates with the standard errors provided in parentheses. Residual variances are represented using a two-headed arrow connecting the residual variable (u) to itself. Standard errors are provided in parentheses. B. Genetic correlation between the two autism factors and a range of mental health, neurodevelopmental, and cognitive traits. Points indicate the estimate, whiskers indicate 95% confidence intervals, and points with an asterisk (*) indicate significant associations with Benjamini-Yekutieli adjustment. Two asterisks (**) indicate phenotypes where the difference in genetic correlation between earlier and later diagnosed autism is statistically significant at P < 0.05.

To formally test whether the varying genetic correlation patterns among the different GWAS of autism emerge from different age at diagnosis correlated latent genetic traits, we modelled the genetic covariance using genomicSEM^54^. GenomicSEM uses structural equation models to identify latent factors. We investigated if two genetic latent traits underlie this heterogeneity, and compared it against five alternative models including a common-factor autism model (Supplementary Table 20). Using six minimally overlapping GWAS for autism with wide variation in age at autism diagnosis, we identified a correlated two-factor model that was the most parsimonious and fit the data best (Akaike information criterion [AIC]: 30.09, confirmatory fit index [CFI]: 1, standardised root mean residual [SRMR]: 0.039, Figure 5A).

Factor 1 (Earlier diagnosed autism factor) was defined by the GWAS with predominantly early childhood diagnosed individuals (PGC-2017, SPARK_before6_). Factor 2 (Later diagnosed autism factor) was defined primarily by GWAS with adolescent or adult diagnosed individuals (iPSYCH_after10_, FinnGen, and SPARK_after10_). The cross loading of iPSYCH_before9_ suggests that Factor 2 may impact behaviours in mid/late childhood as well, leading to a diagnosis before age nine. The two factors had a moderate genetic correlation (r_g_ = 0.38, s.e.m = 0.07). Factor 1 was negatively genetically correlated with both the age at autism GWAS, whilst Factor 2 positively genetically correlated with age at autism diagnosis in SPARK (Figure 5B), confirming that age at autism diagnosis is linked to genetic heterogeneity in autism.

Sensitivity analyses using partly different GWAS identified a two-correlated-factor model as the best fitting model, with similar moderate genetic correlations between the two factors (r_g_ = 0.39 (0.08) - 0.52 (0.09), Supplementary Table 20, Supplementary Figure 10).

## Earlier and later diagnosed autism genetic factors are associated with different mental health profiles

GenomicSEM analyses revealed at least two correlated autism genetic factors. We wondered if these factors are differentially genetically correlated with cognitive, psychiatric and neurodevelopmental traits. Given the higher prevalence of mental health diagnoses in later-diagnosed autistic individuals^18,19^, we hypothesised this might partly stem from differences in shared genetics between these autism factors and other mental health and cognitive phenotypes. We tested this hypothesis using genetic correlation analyses.

The earlier diagnosed autism factor (Factor 1) was positively correlated with educational attainment and cognitive aptitude but had modest genetic correlations with measures of trauma and ADHD (Supplementary Table 21, Figure 5B). The later diagnosed autism factor (Factor 2) had lower genetic correlation with educational attainment but had significant and higher positive genetically correlations with a range of other mental health conditions, including internalising disorders/problems, trauma and related sequelae (Depression, PTSD, childhood maltreatment, and suicide attempts) and ADHD. The iPSYCH female-stratified autism GWAS (iPSYCH_females_) had significantly smaller (P = 0.028) genetic correlation with Factor 1 than the iPSYCH male-stratified autism GWAS (iPSYCH_males_), consistent with epidemiological observations that autistic females are, on average, diagnosed later than males^27^.

Sensitivity analyses using age of diagnosis stratified GWAS from iPSYCH and SPARK yielded largely consistent genetic correlation results (Supplementary Note 6).

We wondered if the genetic signal for later diagnosed autism can be explained by diagnostic misclassification. However, decomposition of the iPSYCH autism genetic signal using genomicSEM indicated that later diagnosed autism cannot be entirely explained by diagnostic misclassification, although genetic effects of ADHD accounted for some of the genetic variance in later diagnosed autism (Supplementary Note 3). Accounting for ADHD’s genetic effects revealed attenuated but significant moderate genetic correlations between iPSYCH_after10_ and mental health conditions, suggesting shared genetics with ADHD do not fully explain the elevated correlation between later diagnosed autism and mental health phenotypes (Supplementary Note 6).

**Figure 6:**
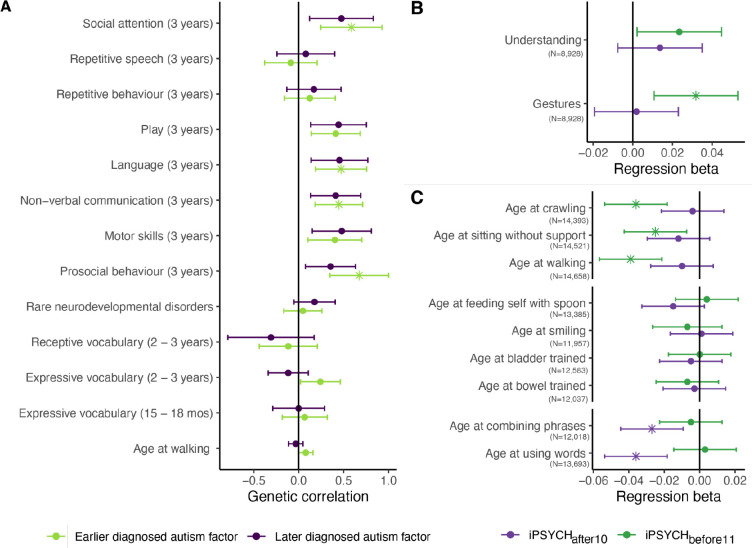
Association between age at diagnosis stratified autism and developmental milestones. A. Genetic correlation between earlier and later diagnosed autism genetic factors and a range of developmental phenotypes. mos = months. B. Association between PGS for iPSYCH_before11_ and iPSYCH_after10_ and social communication skills at 15 months in ALSPAC. N is the sample size. C. Association between PGS for iPSYCH_before11_ and iPSYCH_after10_ and developmental milestones among autistic individuals in SPARK. N is the sample size. For all plots, points indicate the estimate, whiskers indicate 95% confidence intervals, and points with an asterisk (*) indicates significant associations after Benjamini-Yekutieli adjustment. For all plots, positive values indicate greater difficulties/delays.

We further wondered if there is genetic overlap between the earlier diagnosed autism factor and developmental milestones, as delays in developmental milestones are the earliest indicator that a child may be autistic^55^. The earlier but not later diagnosed autism factor was positively and significantly genetically correlated with greater difficulties in social behaviour at age three (Supplementary Table 22, Figure 6A). Supporting these findings, PGS for iPSYCH_before11_ but not iPSYCH_after10_ GWAS was associated with greater difficulties in social communication (gestures) at 15 months (Figure 6B, Supplementary Table 23). Nevertheless, the genetic correlations and regression coefficients did not statistically differ between the two GWAS. Furthermore, PGS for neither iPSYCH_before11_ nor iPSYCH_after10_ were associated with later attainment of developmental milestones among autistic individuals from SPARK. However, these results may reflect collider bias as autistic carriers of rare genetic variants within the cohort have lower autism PGS and substantially delayed developmental milestones^50^ (Supplementary Table 24, Figure 6C).

## Discussion

Understanding why some autistic individuals receive a diagnosis earlier than others has been an important scientific and societal question. Here we show, using multiple methods and datasets, that some of the variability in age at autism diagnosis is linked to differences in socio-behavioural trajectories and associated genetic profiles among autistic individuals. The genetic variation associated with age at autism diagnosis is also associated with the genetic heterogeneity within autism. This relationship can partly explain the often contradictory patterns of genetic correlations between autism and various cognitive, neurodevelopmental, and psychiatric phenotypes across different autism GWAS. This axis of genetic heterogeneity within autism, indexed by age at autism diagnosis, is not fully explained by several other factors that may influence age at autism diagnosis including sex, co-occurring ID and developmental delays, changes to the diagnostic criteria, cohort differences, diagnostic misclassification, or parental factors influencing diagnosis (Supplementary Note 3).

Our findings are consistent with the wider literature that demonstrates that genetic influences on social-communication vary across development in the general population^37,38^. However, extending this line of enquiry, we show that genetic influences on clinically diagnosed autism too vary based on age at diagnosis. Modelling the genetic correlations among various GWAS of autism identified two correlated autism polygenic factors that explained the data better than the alternative models considered, including a single autism factor model (Figure 5). Notably, the genetic correlation between the factors is 0.38, which is similar to the genetic correlation between depression and schizophrenia^56^. It is likely that other dimensions contribute to heterogeneity in autism, including potentially further genetic differences based on age at diagnosis. For example, a significant proportion of the variation in the FinnGen autism GWAS was not explained by either of the two factors (Figure 5A) and the correlation between some autism GWAS (e.g., iPSYCH_females_ and SPARK_before6_) is even lower than the genetic correlation observed between earlier and later diagnosed autism factors.

Importantly, the low genetic correlation between the factors strongly suggests that later diagnosed autism is not merely a broader autism subtype or that they emerge from the same underlying latent genetic distribution. This is further supported by differences in the patterns of genetic correlation between the two autism genetic factors and other phenotypes, and trajectory modelling of SDQ total and subscale scores.

Of the two autism polygenic factors, we found some evidence to suggest that the earlier diagnosed autism genetic factor was associated with social communication difficulties in early childhood (Figure 6). However, neither of the two autism polygenic factors are predominantly driven by a subset of children with co-occurring ID or neurodevelopmental delays. The heritability of age at autism diagnosis does not attenuate when accounting for ID or developmental delays, and there is no association between either factor and neurodevelopmental conditions or delays in developmental milestones (for example, significant delays in walking or first words). Our findings imply that profound neurodevelopmental disabilities among autistic individuals may be aetiologically distinct from the two autism polygenic factors.

Elevated polygenic propensity for the later diagnosed autism genetic factor may lead to less clear difficulties in early childhood and thus may not be picked up by caregivers as reasons to pursue diagnosis or support. These are consistent with findings that children who do not initially meet the criteria for an autism diagnosis may later meet them^7,10^, and with parental reports of on average “milder” autism features among later diagnosed autistic individuals^57^.

In contrast, both the later diagnosed genetic autism factor (Figure 5) and the late emergent latent class of SDQ total scores (Figure 1) are associated with greater mental health problems, particularly internalising difficulties, self-harm, and correlates of trauma. The later diagnosed autism genetic factor is also associated with a larger increase in socio-behavioural difficulties in adolescence. Yet again, this is consistent with the epidemiological findings of greater mental health difficulties among later diagnosed autistic individuals^18,19^. However, our findings demonstrate that part of the epidemiological findings may be explained by genetic heterogeneity in autism indexed by age at autism diagnosis. How exactly these genetic differences lead to greater mental health problems remains to be resolved.

The variation in genetic correlation between ADHD and autism stratified by age of diagnosis is particularly noteworthy. Older GWAS of autism (including the PGC-2017) were not significantly genetically correlated with ADHD^51,58,59^ whereas more recent GWAS for autism have moderate genetic correlations with ADHD^60^. Genetic correlation analyses (Figure 5) indicate that the genetic correlation with ADHD increases with a later diagnosis of autism. We confirmed this using within-family analyses: autistic individuals diagnosed before age five do not over-inherit PGS for ADHD (Supplementary Table 13c). However, even within ADHD, there is genetic heterogeneity based on age at diagnosis^61^. ADHD diagnosed in childhood shows a larger genetic correlation with autism and lower genetic correlation with internalising disorders^62^ compared to late-diagnosed ADHD. These results suggest a complex genetic interplay between autism and ADHD that is dependent on age at diagnosis.

Several sensitivity analyses indicate that our findings are not primarily capturing sex differences (Supplementary Note 3). However, given that autistic females are, on average, diagnosed later than males^4,5^, research that investigates sex and gender differences in both autism and co-occurring conditions^63^ needs to account for genetic confounding associated with age at autism diagnosis. Findings that may be thought to reflect sex differences may additionally reflect differences in age at diagnosis. For example, the higher prevalence of mental health problems in autistic females compared to males^64^ attenuates when restricting to autistic individuals diagnosed before age five^18^.

Although we explored the impact of several additional clinical and demographic factors on age at autism diagnosis, these account for less than 50% of the variance in age at diagnosis (Supplementary Table 8 and Supplementary Note 5). Notably, there is substantial variation across the datasets explored, highlighting that age at diagnosis of autism is immensely complex, varying across geography and time. Local cultural factors, access to healthcare, gender bias, stigma, and camouflaging, all of which are difficult to measure, likely have an impact on who receives a diagnosis and when.

Of interest is camouflaging, which has been hypothesised as one reason for later diagnosis^65^ particularly among autistic females. Our results cannot be fully explained by camouflaging. Although children as young as seven years of age may camouflage^66^, it is unlikely that infants can camouflage behaviours or developmental milestones in infancy or early toddlerhood. Furthermore, even among autistic individuals, there is variation in camouflaging^67,68^. Although we are unable to explicitly test the impact of camouflaging, our results are consistent with the correlates of camouflaging. For instance, it is known that autistic females are more likely to camouflage^69^, and higher camouflaging is associated with greater mental health difficulties^70^, and later autism diagnosis^65,71^.

In conclusion, using genetic data and longitudinal analyses of birth cohorts, we identify an axis of heterogeneity in autism which is indexed by age at autism diagnosis. This axis of heterogeneity partly explains the varying genetic correlations among the different GWAS of autism and between autism and various mental health conditions. These findings provide further support to the hypothesis that the umbrella term “autism” describes multiple phenomena with differing aetiologies, developmental trajectories, and correlations with mental health conditions. These findings have implications for how we conceptualise neurodevelopment more broadly, and for understanding diagnosis, sex and gender differences, and co-occurring health profiles in autism.

## Methods

### A note on terminology

We use the term autistic to refer to people who have an autism diagnosis^72^. We use non-autistic to refer to people who do not have an autism diagnosis. We use sex to refer to sex assigned at birth, and use the terms males and females to refer to sex.

### Analyses of birth cohorts

#### Study design and participants

We used four population-based birth cohorts that vary in ages covered and when data were collected (Extended Figure 1). Briefly, the four cohorts included are the UK-based Millennium Cohort Study (MCS)^73^, the Ireland-based Growing Up in Ireland (GUI) Child cohort (aka Cohort 98’)^74^, and the Australia-based Longitudinal Study of Australian Children - Birth (LSAC-B) and Kindergarten (LSAC-K) cohorts^75^. All children included in the cohorts were born in the 21st century. Further details about the cohorts are provided in the Supplementary Note 1.

As indicated in Supplementary Table 1, these cohorts were adopted for their longitudinal nature, the nationally representative cohort members, and the availability of data on behavioural profiles and neurodevelopmental diagnosis allowing for cross-country comparisons and generalisation^76^.

#### Measures

##### Autism and ADHD diagnosis and age at diagnosis

In all cohorts, across multiple sweeps, the main caregiver was asked if the participant had a diagnosis of autism (Extended Figure 1). For age at diagnosis, we used the age at the sweep when participants first reported being diagnosed as autistic in every cohort, to maximise sample sizes and ensure consistency across cohorts for effective comparisons. For instance, if a participant first reported an autism diagnosis at the age 11 sweep, we considered age at diagnosis to be 11 years. Although the specific age at diagnosis was provided for LSAC-B and LSAC-K, we opted not to use this, as we identified errors in some reports where months and years of diagnosis were swapped or not reported.

In MCS, we had reports of both autism and ADHD diagnoses, allowing us to conduct several sensitivity analyses. For our primary analyses, we included a narrowly defined sample of children with consistently reported autism diagnoses by primary and proxy caregivers (when both were available) and no other reported neurodevelopmental diagnosis (particularly ADHD). To assess the generalizability of our results and increase the sample size, we then expanded the sample to include all children with any reported diagnosis of autism. This expanded sample, which we refer to as “MCS-expanded”, included cases regardless of whether the diagnoses were consistent across sweeps or caregivers, and included those with co-occurring ADHD. Additionally, we imputed the independent variables and covariates for autistic individuals with missing information, as detailed below. We refer to this sample as “MCS-imputed”. Finally, to assess the specificity of the trajectories for autism, we conducted analyses among children who had a consistent ADHD diagnosis but no diagnosis of autism. We refer to this sample as “MCS-ADHD”.

##### Strengths and Difficulties Questionnaire (SDQ)

We used the SDQ to capture social, emotional, and behavioural profiles of participants, with repeated measures from 3 to 18 years across cohorts (Extended Figure 1). SDQ comprises 25 statements that respondents are asked to rate on a 3-point Likert scale (“not true”, “somewhat true”, and “certainly true”) based on the child’s symptoms or behaviours over the past six months. There are five subscales, each containing five items, which assess emotional symptoms, conduct problems, hyperactivity-inattention, peer relationship problems, and prosocial behaviours respectively^20^. The first four subscales assess difficulties, and the total score ranges from 0 to 40, with higher scores indicating more significant difficulties. The fifth subscale represents strengths and has a total score ranging from 0 to 10, with higher scores indicating more prosocial behaviours. The SDQ demonstrates good test-retest reliability and criterion validity across countries^21–23^. Each subscale of SDQ has been found to exhibit correlations with diagnosis of autism and ADHD^77^. Its five-factor structure (each subscale as a factor) has shown consistency and invariance across age, sex, and ethnic background^21,25^. The SDQ captures several core features of mental health and neurodevelopmental conditions, including autism and ADHD^78^. Only children with complete data of SDQ across all sweeps were included in the analyses, except for imputation analyses.

##### Socio-demographic measures

Socio-demographic measures were included as covariates to account for their impact on age at diagnosis in each cohort (Supplementary Table 25). Specific measures and available information vary across cohorts, but we generally included sex, ethnic background, maternal age at delivery, child’s cognitive aptitude, household socio-economic status (SES), and deprivation level of the living area, to account for some factors that may impact the age when someone receives an autism diagnosis^32,79^. Only subsets of children in the complete-SDQ samples, with complete data for these socio-demographic factors, were included in the respective analyses, resulting in a further reduction in sample sizes.

In MCS, although various census classifications for ethnic groups were available, we opted to use a binary indicator to identify non-white ethnic minorities. This approach was chosen to maintain consistency with other cohorts. Ethnicity data were not collected in either LSAC cohort. Instead, visible ethnic minority status was determined primarily by parental country of birth and the language(s) spoken at home.^80^ Maternal age at delivery was collected only in MCS. In other cohorts, we used maternal age (in years) at first sweep of data collection to reflect the variation in maternal age at delivery.

For cognitive aptitudes and other socio-demographic factors, including SES and deprivation, we adopted summary scores using principal component analysis (PCA) to capture information measured by diverse scales. However, as including more social factors lead to smaller sample sizes, we prioritised factors based on the availability of data among already limited autistic samples. Information on socio-demographic factors in each cohort, including variables and scales included in PCA and resulting sample sizes, can be found in Supplementary Table 25. Note, in the birth cohorts, no autistic child was identified as having intellectual disability (ID), defined as scoring two or more standard deviations below the mean value of the first principal component score (‘g’ factor) derived from multiple cognitive aptitude measures in corresponding cohort.

#### Statistical analyses

Following the participant selection process, we used two methods to model the longitudinal trajectories of SDQ total and subscale scores. In the first analyses, we *a priori* defined two groups of autistic individuals - childhood diagnosed (diagnosed before ages 9 – 11, depending on the cohort), and adolescent diagnosed (diagnosed after the ages of 9 – 11, depending on the cohort). This age period was chosen as there is epidemiological evidence showing increased autism incidence among females during this window^27^ and because trajectory analyses have identified increasing autism-related traits in a subset of individuals after this period ^81^. We were also limited in choosing alternate cutoffs due to the absence of information on both SDQ and autism diagnosis at earlier and later time points in some of the cohorts, and the relatively low sample sizes of the resulting groups.

Anyone who had no report of an autism diagnosis were included in the general population group. For MCS in particular, children with neither autism nor ADHD diagnosis were included in the general population group. We used linear Latent Growth Curve Models (LGCM) to identify the latent trajectories of SDQ total and subscale scores in the three groups (childhood diagnosed, adolescent diagnosed, and the general population) for all cohorts. Each linear model included a latent intercept to represent the initial level of the outcome variable, and a linear latent slope to represent the mean rate of change over time. As sensitivity analyses, quadratic models were also fitted in MCS, MCS-expanded, and LSAC-B, in which quadratic time scores were assigned across sweeps respectively to capture this nonlinear change over time. However, quadratic models for most subscales among the three cohorts either did not converge or demonstrated Heywood cases, i.e., negative variance estimates, in slope terms This likely stems from insufficient statistical power due to the small sample sizes or model misspecification, which hinders meaningful theoretical interpretation. Therefore, we decided to use linear models.

Given the well-known sex differences in diagnosis age^27^, we also applied the same models stratified by sex, i.e., estimating latent intercept and slope for each sex, within the autistic samples. All LGCM were fitted under the structural equation modelling framework using the *lavaan* package in R^82^.

In parallel, we conducted Growth Mixture Models (GMM) to identify if there were latent groups of autistic individuals based on their trajectories of SDQ total and subscale scores. GMM assumes that the sample being studied consists of multiple mixed effects models, each capturing a subgroup trajectory with shared intercept and slope^83^. We fitted models with one to four groups for each subscale and SDQ total scores in each cohort, using the *lcmm* package in R^84^. The optimal number of latent trajectories were then determined by comparing fit indices, including Bayesian Information Criterion (BIC) values, classification quality measure (entropy), and substantive interpretation. Models with lower BIC values and higher entropy are favoured^85^. Also, models identifying subgroups with less than 5% of the sample size were not considered for poor statistical reliability and limited practical significance^86^.

Multiple regressions were subsequently conducted to investigate the association between individuals’ age at diagnosis (the outcome variable) and their SDQ total and subscale latent class memberships identified in optimal GMM, as well as other socio-demographic covariates. We did not detect any multicollinearity among the variables using variance inflation factors. Estimates of coefficients of predictors and corresponding p-values were interpreted to determine which factors contribute to differing age at autism diagnosis.

Additionally, considering the limited sample sizes and the number of explanatory variables included, the relative importance of each predictor was assessed using dominance analysis^87^. We employed the *misty*^88^ package in R for this analysis, using a correlation matrix extracted from the fitted model via the *lavInspect* function from the *lavaan* package^82^. This approach leverages the correlation matrix to consider not only individual predictors but also the correlations among them, providing a more comprehensive assessment of their relative importance^89^.

To examine potential causal pathways, mediation analyses were conducted, allowing socio-demographic factors to indirectly influence the age at diagnosis through their effects on latent class memberships identified in the optimal GMM. Using structural equation modelling in the *lavaan* package^82^, both direct and indirect effects were assessed, with their significance calculated using bootstrapping analysis. Further details are provided in Supplementary Note 5.

To investigate the specificity of our findings to autism, we conducted LGCM, GMM, and sequential mediation analyses in individuals with ADHD but without a co-occurring autism diagnosis in the MCS cohort (N = 89, Supplementary Table 4). ADHD diagnoses were available in the same sweeps as autism diagnoses, reported at age 5,7,11, and 14. Carers were asked the following question: ‘Has a doctor or other health professional ever told you that <child’s name> had Attention Deficit Hyperactivity Disorder (ADHD)?’.

In autistic individuals, using the GMM based latent classes of the SDQ total scores, we used multiple regression to investigate the association with mental health phenotypes in MCS, LSAC-K, and LSAC-B. We included sex as a covariate.

##### Imputation

To assess the impact of missingness, we applied *softImpute*, to impute missing data for all children with an autism diagnosis reported by any carer in any sweep in the MCS cohort (N = 623, Supplementary Table 3). Given the longitudinal nature of data collections for SDQ subscale scores and some cognitive aptitude measures, SoftImpute was chosen for its computational efficiency in handling large-scale matrices through low-rank approximation, effectively preserving underlying structure of input data. To enhance imputation quality and reduce bias, we included related auxiliary variables in the imputation process, along with SDQ subscale scores in all available sweeps (Supplementary Table 3). Further information is provided in Supplementary Note 2.

#### SPARK cohort: Genotyping, quality control and imputation

We used data from the SPARK cohort^39^ iWES2 v1 dataset (released in Feb 2022) which included data from 70,487 autistic individuals and their families. All participants were genotyped using the Illumina Global Screening Array (GSA_24v2–0_A2). To avoid false positives due to fine-scale population stratification, we restricted the analyses to individuals of genetically-inferred European ancestries (N = 51,869 autistic and non-autistic participants), which was provided by the SPARK consortium. From this, we restricted to individuals with genotyping rate > 98%, individuals without sex mismatches and excess heterozygosity (3 standard deviations from the mean heterozygosity), and where trio data was available, trios with fewer than 5% Mendelian errors, resulting in 47,170 autistic and non-autistic individuals. We included genetic variants with minor allele frequency > 1%, genotyping rate > 95%, and that were in Hardy Weinberg Equilibrium (HWE p-value > 1×10^−6^), resulting in 518,189 SNPs.

We used this quality controlled genotype data for imputation, calculating genetic principal components, and inferring relatedness among individuals. We inferred genetic relatedness using KING^90^. For genetic principal component analysis, we pruned SNPs for linkage disequilibrium (LD) (maximum r^2^ = 0.1) and removed the human leukocyte antigen (HLA) region. Using PC-AiR^91^, we first calculated principal components (PCs) in genetically unrelated individuals and then projected the PCs onto related individuals. We imputed genotypes using the TOPMED imputation panel^92^ on the Michigan imputation server (v1.7.3)^93^ using Minimac4^93^ and after phasing using Eagle v2.5^94^. Post imputation, variants were converted from GRCh38/hg38 to GRCh37/hg19 using liftOver. We restricted downstream analyses only to variants with minor allele frequency > 0.1% and with an imputation R^2^ > 0.6.

#### SPARK cohort: Association analyses

##### PGS association analyses

Polygenic scores (PGS) were calculated using PRScs^95^ which uses a Bayesian shrinkage prior. PGS were calculated for autism (iPSYCH only dataset, N = 19,870 autistic individuals and 39,078 non-autistic individuals)^50,96^, ADHD^40^, bipolar disorder^97^, major depressive disorder^44^, schizophrenia^41^, educational attainment^98^, and cognitive aptitude^46^, autism diagnosed before age 11 (iPSYCH_before11_), and autism diagnosed after age 10 (iPSYCH_after10_). The latter two GWAS were generated using the iPSYCH2015^96^ cohort, details of which are provided below. For simplicity we refer to this cohort as iPSYCH throughout.

We ran association analyses between PGS and age at autism diagnosis (converted to years in all analyses) in the quality controlled dataset. We excluded individuals older than 22 to focus on those who had an autism diagnosis using either the DSM-IV^99^ or DSM-5, retaining a maximum of 18,809 autistic individuals for PGS analyses. This criteria also allowed us to focus on individuals who received their diagnosis in childhood or adolescence, as older adults may have missed an earlier diagnosis of autism due to secular changes in social attitudes towards autism. For psychiatric conditions, we ran separate linear regressions with age at autism diagnosis and the aforementioned PGS. The baseline model included ID (caregiver reported), sex, and the first 10 genetic principal components as covariates. For schizophrenia, ADHD, depression, cognitive aptitude, educational attainment, iPSYCH_before11_ and iPSYCH_after10_, we ran sensitivity analyses by sequentially including age at walking and age at first words (developmental milestones), parental occupation, highest parental education, and household income (together, socio-economic status or SES), and national area deprivation percentile (deprivation) as covariates. We also included trio status in the baseline model to account for potential participation bias. Additionally, for iPSYCH_before11_ and iPSYCH_after10_ we included any diagnosis of an attentional or behavioural disorder as a covariate in the baseline model to account for diagnostic overshadowing. For the PGS with iPSYCH_before11_ and iPSYCH_after10_, we ran sensitivity analyses after stratifying by sex.

We tested if the effects varied by sex by including a PGS by sex interaction term in the baseline model.

We tested for direct and indirect genetic effects of ADHD and educational attainment (EA) PGS in two ways. First, we generated pseudocontrols for complete trios in Plink 1.9^100,101^, and calculated PGS separately for autistic individuals and pseudocontrols. We regressed the effects of the ADHD and EA PGS for autistic individuals and pseudocontrols on age at autism diagnosis. We included sex, ID (caregiver reported), and the first 10 genetic principal components as covariates. The direct effects were calculated by subtracting the effects of the untransmitted PGS (indirect effect) from the transmitted PGS (total effects). Standard errors and p-values were calculated by bootstrapping 10,000 times as done previously^102^.

Second, in complete trios, we used polygenic transmission disequilibrium tests (pTDT)^103^ to calculate the deviation of PGS from the parental mean PGS for ADHD and EA. We checked for over-transmission of PGS stratified by sextiles based on age at autism diagnosis.

We used pTDT to investigate if there is an over-transmission of PGS for iPSYCH_before11_ and iPSYCH_after10_ among autistic individuals in the SPARK cohort.

In the SPARK cohort, we obtained data for age at achieving nine developmental milestones (in months) for autistic individuals. For all milestones, we excluded individuals who were greater than five median absolute deviations from the median. We ran multiple linear regression with PGS for iPSYCH_before11_ and iPSYCH_after10_ in which we included sex, age at recruitment into the study, and the first 10 genetic principal components as covariates.

##### Rare high-impact de novo variants and inherited variantes

We identified rare (minor allele frequency [MAF] < 0.1%) *de novo* and inherited variants in complete trios from SPARK as previously described^104^. We identified high impact protein truncating variants by restricting variants in loss-of-function observed/expected upper bound fraction (LOEUF)^105^ highly constrained decile (LOEUF < 0.37) that were annotated as either frameshift, stop gained, or start lost; and had a loss-of-function transcript effect estimator (LOFTEE) “high confidence annotation”. To identify high-impact *de novo* missense variants, we restricted to variants in LOEUF highly constrained genes (LOEUF < 0.37), and had an MPC (missense badness, PolyPhen-2, and constraint) score^106^ > 2. All variants were rare, with an allele frequency < 0.1% in SPARK and gnomAD.

We ran regression analyses separately for high-impact *de novo* and inherited protein truncating variants and missense variants, and additionally by combining both protein truncating and missense variants. We included sex and age at recruitment into the study as covariates for analyses with *de novo* variants, and additionally the first 10 genetic principal components for analyses with inherited variants.

#### GWAS for age at autism diagnosis and age at diagnosis stratified autism

We generated a GWAS of age at autism diagnosis (in years) on the quality controlled dataset from SPARK, restricting it to individuals who were under 22 years of age (N = 18,809), and SNPs with a MAF > 1%. GWAS was generated using FastGWA^107^ with age at recruitment into the study, sex, ID, and the first 10 genetic principal components as covariates. In iPSYCH, we generated an additional GWAS of age at autism diagnosis (in years) in a quality controlled dataset of unrelated individuals with sex and ID included as covariates using FastGWA^107^, restricting to SNPs with an MAF > 1%. To keep it consistent with SPARK, we excluded individuals who were diagnosed after age 22, resulting in 18,965 individuals. Briefly, pre-imputation quality control of the iPSYCH data was performed using the Ricopili pipeline^108^, prephased using Eagle v.2.3.5, and imputated using Minimac3^109^, using the downloadable version of the Haplotype Reference Consortium (HRC)^110^ (accession no. EGAD00001002729). Further details of quality control and imputation are provided in Als et al., 2023^44^.

We additionally generated three age at autism diagnosis stratified GWAS in SPARK using (unscreened) non-autistic parents and siblings as controls (N_control_ = 24,965). The three GWAS were: (1) SPARK, diagnosed before age 6 (SPARK_before6_; N_autistic_ = 14,578); (2) SPARK, diagnosed before age 11 (SPARK_before11_, N_autistic_ = 18,719); and (3) SPARK, diagnosed after age 10 (SPARK_after10_, N_autistic_ = 3,358). For these analyses, we did not restrict it to individuals under 22 to increase sample size. GWAS were generated using quality controlled SNPs with a MAF > 1% using FastGWA-GLMM^111^. We included age at recruitment into the study (to account for parental controls potentially lacking autism diagnoses due to historical diagnostic changes), sex and the first 10 genetic principal components as covariates. Fast-GWA GLMM can account for relatedness and fine-scale population stratification even in family-based samples like SPARK.

Although inclusion of unscreened related individuals as controls can decrease heritability and statistical power to identify loci^112^, we used the GWAS to primarily conduct genetic correlation and related analyses. To ensure the robustness of these models we: (1) confirmed that the attenuation ratio for all GWAS was not significantly greater than 1; (2) generated an additional GWAS of SPARK without stratifying by age at autism diagnosis using the same methods and confirmed a high genetic correlation (r_g_ = 0.92, s.e.m = 0.17) with a previous SPARK GWAS^52^ which used a case-pseudocontrol approach; and (3) in the genomicSEM analyses, ran sensitivity analyses using a trio-based SPARK GWAS in lieu of the age at diagnosis stratified GWAS from SPARK and confirmed our findings.

We generated four age at diagnosis stratified GWAS of autism in iPSYCH cohort^96^. The primary GWAS used in the analyses were GWAS of autism diagnosed before age 11 (iPSYCH_before11_: 9,500 autistic and 36,667 non-autistic individuals) individuals and autism diagnosed after age 10 (iPSYCH_after10_: 9,231 autistic and 36,667 non-autistic individuals). We chose this age cutoff to divide the iPSYCH cohort into two subgroups with similar sample sizes and because age coincided with the window in which we observe an increase in SDQ scores in the birth cohorts, and which is associated with an increase in diagnosis of females in epidemiological samples^27^.

We conducted two additional GWAS with smaller sample sizes: GWAS of autism diagnosed before age nine (iPSYCH_before9_: 5,451 autistic and 36,667 non-autistic individuals) and after age 11 (iPSYCH_after11_: 8,260 autistic and 36,667 non-autistic individuals). These were used in sensitivity analyses. For the last two GWAS, we also conducted GWAS after excluding individuals born after 1994, to ensure that all autistic individuals received a diagnosis using either DSM-IV or DSM-5 criteria. However, we observed high genetic correlation between the GWAS when using the full sample and when restricting the sample to those born after 1994, suggesting that changes in the diagnostic criteria do not substantially impact the genetic analyses. To increase sample size and statistical power, we conducted all downstream analyses without excluding autistic individuals born before 1994.

All individuals included in these GWAS from iPSYCH were born between May 1980 and December 2008 to mothers who were living in Denmark. GWAS was conducted on individuals of European ancestry, with the first 10 genetic principal components included as covariates using logistic regression as provided in PLINK.

#### Heritability, genetic correlation, and genomicSEM

Heritability analyses for age at autism diagnosis were conducted using a single-component genome-wide complex trait analysis with genomic-relatedness-based restricted maximum likelihood approach (GCTA-GREML)^113,114^ in unrelated autistic individuals using the quality controlled genetic data in SPARK. We estimated SNP-based heritability first after including sex, age, and the first ten genetic principal components as covariates. We ran sensitivity analyses after sequentially including ID, developmental milestones, SES and area deprivation as covariates.

SNP-based heritability for the age at diagnosis stratified autism GWAS from iPSYCH was calculated using linkage disequilibrium score regression coefficient (LDSC)^58,115^ using linkage disequilibrium scores from the north-west European population. We converted observed scale heritability estimates to liability scale estimates using a range of autism lifetime prevalence estimates, including a “best guess” autism lifetime prevalence estimate for each of the age-stratified autism GWAS.

We conducted genetic correlation analyses using LDSC, using linkage disequilibrium scores from the north-west European populations.

For genomicSEM^54^ analyses, we first conducted genetic correlation analyses among fourteen different autism GWAS using LDSC. This included a multi-ancestry case-pseudocontrol GWAS in SPARK^52^ (6,222 case-pseudocontrol pairs); GWAS from FinnGen (Data Release - r10)^116^ (646 cases and 301,879 controls), the PGC-2017 autism GWAS^51^ (7,387 cases and 8,567 controls), seven GWAS from iPSYCH, and age at diagnosis stratified GWAS from SPARK. The iPSYCH GWAS included an unstratified (19,870 autistic individuals [15,025 males and 4,845 females] and 39,078 controls) and sex-stratified GWAS^50^, and four age at diagnosis stratified GWAS as mentioned earlier.

Subsequently, we restricted to six GWAS with minimal sample overlap, without high genetic correlation (r_g_ > 0.95), and with wide variation in age at diagnosis to conduct genomicSEM analyses using autosomes. Using the patterns of genetic correlations observed we tested an age at diagnosis related correlated two-factor model. We additionally tested: (1) a single-factor model; (2) a correlated two-factor “geography” model where three US-based autism GWAS loaded onto one factor, and three Europe-based autism GWAS loaded onto a second factor; (3) a bifactor model based on age at diagnosis; (4) a bifactor model based on the geography of the cohorts; and (5) a hierarchical factor model based on age at diagnosis. The two-factor model was chosen as it had lower RMSEA and higher CFI and was more parsimonious than the bifactor model. We ran sensitivity analyses using different GWAS of autism as input and confirmed that the two-correlated-factor model was the best fitting model of the models tested.

#### Analyses in ALSPAC and MCS

##### Genetic quality control

We obtained quality controlled and imputed genotype data from ALSPAC^117–119^. Further details about the cohort are provided in the Supplementary Note 1. Briefly, ALSPAC children were genotyped using the Illumina HumanHap550 quad chip genotyping platforms by 23andme. Individuals were excluded due to sex mismatches, excess heterozygosity, missingness > 3%, and insufficient sample replication (Identical-By-Descent [IBD] < 0.8). After multidimensional scaling, and comparison with Hapmap II (release 22), only individuals of genetically inferred European ancestries were retained. SNPs with low frequency (MAF < 1%), poor genotyping (call rate < 95%) and deviations from Hardy-Weinberg equilibrium (P < 5×10^−7^) were removed. 9,115 subjects and 500,527 SNPs passed quality control. Genotypes were phased using ShapeIT, and imputation was done using the Haplotype Reference Consortium panel using the Michigan imputation server. After imputation, we further removed low frequency SNPs (MAF < 1%). Further details of the quality control and imputation of ALSPAC are provided here: https://proposals.epi.bristol.ac.uk/alspac_omics_data_catalogue.html#org89bb79b. Genome-wide genotype data was generated by Sample Logistics and Genotyping Facilities at Wellcome Sanger Institute and LabCorp (Laboratory Corporation of America) using support from 23andMe.

We also obtained quality controlled and imputed data from MCS. Briefly, MCS samples were genotyped using the Illumina Global Screening Array^120^. Individuals were excluded due to sex mismatches, excess heterozygosity, and missingness > 2%. We identified European samples using the GenoPred pipeline^121^ (https://github.com/opain/GenoPred). SNPs with low frequency (MAF < 1%), poor genotyping (call rate < 97%) and deviations from Hardy-Weinberg equilibrium (p < 1×10^−6^) were removed. Imputation was conducted using Minimac4^93^ using the TOPMED reference panel^92^ in the Michigan imputation server^93^. Post imputation, SNPs with an imputation R^2^ INFO score < 0.8, with > 3% missing, and with a MAF < 1% were excluded. Further details are available here: https://cls-genetics.github.io/docs/MCS.html

PGS for both ALSPAC and MCS were calculated in individuals of genetically inferred European ancestries. Genetic principal components were calculated for both cohorts using PC-AiR as described earlier. We calculated PGS for iPSYCH_before11_ and iPSYCH_after10_ and used these in all analyses in the MCS to keep it consistent with analyses in SPARK where we could only use the iPSYCH GWAS to avoid overlap between the training and testing sample.

##### Association with SDQ

We obtained scores on the SDQ total and subscales for six ages in the MCS and five ages in ALSPAC. We ran cross-sectional analysis at each age using multiple linear regression with PGS for iPSYCH_before11_ and iPSYCH_after10,_ with sex, age, and the first 10 genetic principal components as covariates. Additionally, we ran multiple linear mixed effects regression using *lme4* package in R^122^, fitting a PGS by age interaction term to investigate if the effects of PGS on SDQ change over time.

To investigate if the differences in association between MCS and ALSPAC were due to differences in ascertainment between the two cohorts, we matched ALSPAC to MCS using entropy balancing^123^ and re-ran the PGS association analyses. Entropy balancing is a reweighting technique that ensures the covariate distributions are identical between groups. This method uses optimisation algorithms to assign weights to individuals such that the weighted average of the covariates in ALSPAC (the larger genotyped cohort) matches that of MCS (the smaller genotyped cohort), minimising confounding biases and increasing comparability. We used the child’s biological sex, maternal age at delivery, and maternal highest educational qualification at first data collection in each cohort as matching factors. We considered using propensity score matching with a 1:1 ratio to obtain a well-balanced subsample of ALSPAC, yet this approach would have resulted in substantial data loss and potential risk of residual confounding due to limited covariate selection. Therefore, we opted for entropy balancing to retain a larger sample size in the ALSPAC cohort. Entropy balancing was conducted using the *ebal* package in R^124^.

##### Association with developmental milestones and autism diagnosis

In ALSPAC, we obtained understanding of simple phrases (e.g., “do you want that”, or “come here”) and gesture scores from the Macarthur-Bates Communicative Development Inventories^125^ at 15 months of age. We conducted multiple linear regression using PGS for iPSYCH_before11_ and iPSYCH_after10_, with sex, age, and the first 10 genetic principal components as covariates.

Autism diagnosis in the MCS was obtained using parent/caregiver reports of autism/asperger syndrome diagnosis by a doctor at ages 5, 7, 11, and 14. We identified individuals with an autism diagnosis at age 7 or earlier, age 11 or earlier, or between ages 11 and 14. We conducted Firth’s bias-reduced multiple logistic regression (*logistf* package in R) using PGS for iPSYCH_before11_ and iPSYCH_after10_, with sex, age and the first 10 genetic principal components covariates.

## Extended Data

## Supplementary Material

Supplement 1

Supplement 2

## Data Availability

SPARK autism GWAS: https://bitbucket.org/steinlabunc/spark_asd_sumstats/src Finngen autism GWAS: https://www.finngen.fi/en/access_results iPSYCH autism GWAS (unstratified, sex-stratified and age at diagnosis stratified) can be obtained from Anders Borglum and Jakob Grove. Psychiatric GWAS summary stats: https://pgc.unc.edu/ GWAS educational attainment: https://thessgac.com/ GWAS cognitive aptitude: https://cncr.nl/research/summary_statistics/ For ALSPAC, the study website contains details of all the data that is available through a fully searchable data dictionary and variable search tool”: http://www.bristol.ac.uk/alspac/researchers/our-data/ For MCS, data can be obtain after application through the UK Data Service: https://beta.ukdataservice.ac.uk/datacatalogue/series/series?id=2000031 Summary statistics for the SPARK based GWAS will be made upon publication.
