# Supplementary material for: An axis of genetic heterogeneity in autism is indexed by age at diagnosis and is associated with varying developmental and mental health profiles
Source: medRxiv. 2024 Aug 2:2024.07.31.24311279. Preprint. [Version 1] doi: 10.1101/2024.07.31.24311279 (PMC11312648; doi:10.1101/2024.07.31.24311279)
Supplement: Supplementary file 1 [file media-1.pdf]

Supplementary Notes, Figures, and FAQs

Note 1: Overview of the birth cohorts **2**
Millennium Cohort Study 2
Longitudinal Study of Australian Children (Birth and Kindergarten) 2
Growing Up in Ireland 3
ALSPAC 3
Note 2: Imputation **5**
Note 3: Impact of various demographic and clinical characteristics on age at autism diagnosis **9**
1. Clinical factors: Changes in diagnostic criteria over time 9
2. Clinical factors: Diagnostic misclassification 10
3. Clinical factors: Intellectual disability and co-occurring developmental delays 13
4. Clinical factors: Country/continent level differences in diagnosis 14
5. Demographic factors: Sex 14
6. Demographic factors: Parental characteristics 15
Note 4: GMM river plots and explanation **16**
Note 5: Mediation analyses **21**
Note 6: Sensitivity analyses of the genetic correlation results **22**
Summary **24**
FAQs **25**
Glossary of terms **29**
Supplementary Figure 1: Conduct problems **30**
Supplementary Figure 2: Emotional symptoms **31**
Supplementary Figure 3: Hyperactivity/Inattention **32**
Supplementary Figure 4: Peer relationship problems **33**
Supplementary Figure 5: Prosocial behaviour **34**
Supplementary Figure 6: Results from GUI **35**
Supplementary Figure 7: Sensitivity analyses for ADHD **36**
Supplementary Figure 8: Sensitivity analyses for ADHD (cont.) **37**
Supplementary Figure 9: Genetic correlation between age at autism diagnosis and autism GWAS ordered by sex and median age at diagnosis. **38**
Supplementary Figure 10: GenomicSEM models with alternate GWAS **39**
References **40**

Note 1: Overview of the birth cohorts

Millennium Cohort Study

The Millennium Cohort Study is a longitudinal study that follows the lives of approximately 19,000 children born in the early years of the 21st century, along with their families, across the United Kingdom. The study participants were born over a 12-month period starting in September 2000 in England and Wales, and over a 13.5-month period starting in November 2000 in Scotland and Northern Ireland.

The sample design ensured an overrepresentation of families residing in areas with high levels of child poverty and areas in England with significant ethnic minority populations. The initial data collection was conducted through a home-based survey when the children were nine months old, gathering information on various aspects, including circumstances surrounding pregnancy and birth, early life experiences, and the socio-economic backgrounds of the families. Subsequent data collection waves have occurred at ages 3, 5, 7, 11, 14, and 17, allowing researchers to track the development and life trajectories of these children as they progress through various stages of life.

Ethical approval was obtained from Multi-centre Research Ethics Committees.

Longitudinal Study of Australian Children (Birth and Kindergarten)

Growing Up in Australia: The Longitudinal Study of Australian Children (LSAC) is a longitudinal study that follows two cohorts of about 5,000 children and their families randomly selected from across Australia. The sampling strategy ensured that the number of children selected can reflect the overall child population distribution across states/territories. The B ("baby") cohort comprises children born between March 2003 and February 2004 (aged 0-1 years in the first data collection sweep); the K ("kindergarten") cohort comprises children born between March 1999 and February 2000 (aged 4-5 years in the first data collection sweep). Since 2004, data collections have happened biennially using multiple methods, including face-to-face interviews, computer-assisted telephone interviews, self-completed questionnaires, physical measures, and linking to administrative data (e.g., MySchool). From Sweep 3 onwards, there is data on children of the same age from both cohorts at different time points, featuring the unique LSAC "accelerated cross-sequential" design.

By systematically tracking various facets of children's environments—encompassing social, economic, familial, and educational dimensions—LSAC seeks to identify opportunities for early intervention and inform policy decisions aimed at enhancing overall well-being and support systems for children.

	Sweep 1 (2004)	Sweep 2 (2006)	Sweep 3 (2008)	Sweep 4 (2010)	Sweep 5 (2012)	Sweep 6 (2014)	Sweep 7 (2016)	Sweep 8 (2018)	Sweep 9 (2020-21)
**B Cohort**	0-1 Years	2-3 Years	4-5 Years	6-7 Years	8-9 Years	10-11 Years	12-13 Years	13-14 Years	15-16 Years
**K Cohort**	4-5 Years	6-7 Years	7-8 Years	9-10 Years	11-12 Years	12-13 Years	13-14 Years	15-16 Years	17-18 Years

Growing Up in Ireland
Growing up in Ireland (GUI) is a longitudinal study that follows children living in the Republic of Ireland. We used one of the two cohorts, Cohort 98' (aka the Child Cohort), who were born in 1998. Since 2008, data collections have been conducted every four years, from parents, teachers, and young people themselves. We did not study the other cohort, Cohort 08' (aka the Infant Cohort), due to the unavailability of information on autism diagnoses in the earliest few sweeps.

The GUI study aims to describe and understand the lives of children in Ireland, with Cohort 98' tracking their development from childhood to adulthood and identifying key factors impacting their well-being. It also seeks to examine the effects of early childhood experiences on later life outcomes, map variations in children's lives, gather children's perspectives, and provide data to inform the development of effective policies and services for children and families.

ALSPAC
Pregnant women resident in Avon, UK with expected dates of delivery between 1st April 1991 and 31st December 1992 were invited to take part in the study. 20,248 pregnancies have been identified as being eligible and the initial number of pregnancies enrolled was 14,541. Of the initial pregnancies, there were a total of 14,676 foetuses, resulting in 14,062 live births and 13,988 children who were alive at 1 year of age. When the oldest children were approximately 7 years of age, an attempt was made to bolster the initial sample with eligible cases who had failed to join the study originally. As a result, when considering variables collected from the age of seven onwards (and potentially abstracted from obstetric notes) there are data available for more than the 14,541 pregnancies mentioned above: The number of new pregnancies not in the initial sample (known as Phase I enrolment) that are currently represented in the released data and reflecting enrolment status at the age of 24 is 906, resulting in an additional 913 children being enrolled. The total sample size for analyses after the age of seven is therefore 15,447 pregnancies, resulting in 15,658 foetuses. Of these 14,901 children were alive at 1 year of age.

Of the original 14,541 initial pregnancies, 338 were from women who had already enrolled with a previous pregnancy, meaning 14,203 unique mothers were initially participating in the study. As a result of the additional phases of recruitment, a further

630 women who did not enrol originally have provided data since their child was 7 years of age. This provides a total of 14,833 unique women (G0 mothers) enrolled in ALSPAC as of September 2021.

Please note that the study website contains details of all the data that is available through a fully searchable data dictionary and variable search tool: http://www.bristol.ac.uk/alspac/researchers/our-data/

Ethical approval for the study was obtained from the ALSPAC Ethics and Law Committee and the Local Research Ethics Committees. Consent for biological samples has been collected in accordance with the Human Tissue Act (2004)[1]. Informed consent for the use of data collected via questionnaires and clinics was obtained from participants following the recommendations of the ALSPAC Ethics and Law Committee at the time.

Note 2: Imputation

To handle missing data in the MCS cohort, we considered several imputation methods, each with distinct advantages and limitations.

For example, median imputation is a simple and computationally efficient method that replaces missing values with the median of the observed values for a given variable. While this method is straightforward and preserves the central tendency of the data, it does not account for relationships between variables or the longitudinal structure of the data. Thus, it can lead to biassed estimates and reduced variability, which may not be suitable for complex, large-scale datasets like the MCS.

Multiple Imputation by Chained Equations (MICE) is another commonly used method. It involves creating multiple complete datasets by imputing missing values iteratively based on predictive models for each variable. Each iteration involves fitting a model to predict the missing values of one variable, given the others. This process is repeated for all variables with missing data, cycling through them until all missing data are imputed. Although MICE can handle a variety of data types and relationships, determining the optimal imputation model within MICE in this study can be challenging. Typically, linear regression models are used for continuous variables, logistic regression for binary variables, and multinomial regression for categorical variables. But in our study, the complexity of the relationships between variables, their interactions over time, and the mixture of variable types complicate the process of defining appropriate models for MICE.

Given these considerations, we opted for SoftImpute[2], an imputation method based on matrix completion techniques. SoftImpute employs a soft-thresholding operation on the singular value decomposition (SVD) of the incomplete data matrix, iteratively refining the approximation of missing values. Leveraging low-rank matrix approximation, SoftImpute is particularly suitable for large-scale datasets due to its computational efficiency. Moreover, by focusing on the underlying structure of the data instead of fitting specific predictive models for each variable type, as in MICE, SoftImpute can effectively capture and preserve complex relationships and interactions over time in our data.

In this study, the regularisation parameter λ was set to the largest possible values, determined by the maximum singular value obtained from the Singular Value Decomposition (SVD) of the input matrix. This choice encourages simpler solutions with lower-rank structures, promoting generalisation and preventing overfitting of the imputed data. Also, the maximum rank (*rank.max*), which determines the maximum number of singular values to retain during the low-rank approximation, was set to be one less than the minimum dimension of the input matrix, enabling retention of as many patterns as possible while maintaining computational efficiency. Auxiliary variables, including socio-demographic variables and developmental milestones, were included in the imputation to improve the precision of estimated values (**Supplementary Table 3**).

Visual inspection of density plots (**Note 2, Figures 1 and 2**) and *Kolmogorov-Smirnov (KS)* tests demonstrated that the distributions of imputed data restored that of the original data (see **Supplementary Table 3**). Although there was

less satisfactory imputation quality observed for subscale scores at the age 17 sweep, likely due to the expected highest sweep attrition.

SDQ total scores were then calculated from imputed subscale scores in the corresponding sweeps. To generate principal components of socio-demographic factors and cognition, imputed data were combined with the non-imputed raw data in MCS. Subsequently, principal component analyses were conducted separately for (i) cognitive aptitude measures; (ii) household socio-economic status; and (iii) living area deprivation following the procedures outlined in **Methods.**

In the following latent growth curve models, due to the significantly larger sample size compared to other cohorts used in this study, most estimated latent slopes were statistically significant, confirming the phenomena observed in non-imputed samples. In GMM, more than two latent trajectory groups were identified as optimal across four of the subscales. However, the most prominent drop in BIC value was from models with one to those with two latent groups. For SDQ total scores, GMMs still identified two latent trajectory groups as optimal, confirming previous findings. In subsequent regression analyses, the model with group memberships derived from two-group GMMs explained greater variance in the age at diagnosis, compared to the model using group memberships taken from the optimal GMMs of each subscale (R^2 = 0.59, adjusted R^2 = 0.51, see **Supplementary Table 3**). Thus, group memberships derived from the two-group GMMs were used in the final mediation analyses. All variables together explained 59.8% of the variance in age at diagnosis, and trajectory groups of SDQ total and subscale scores explained 56.6% of the variance collectively.

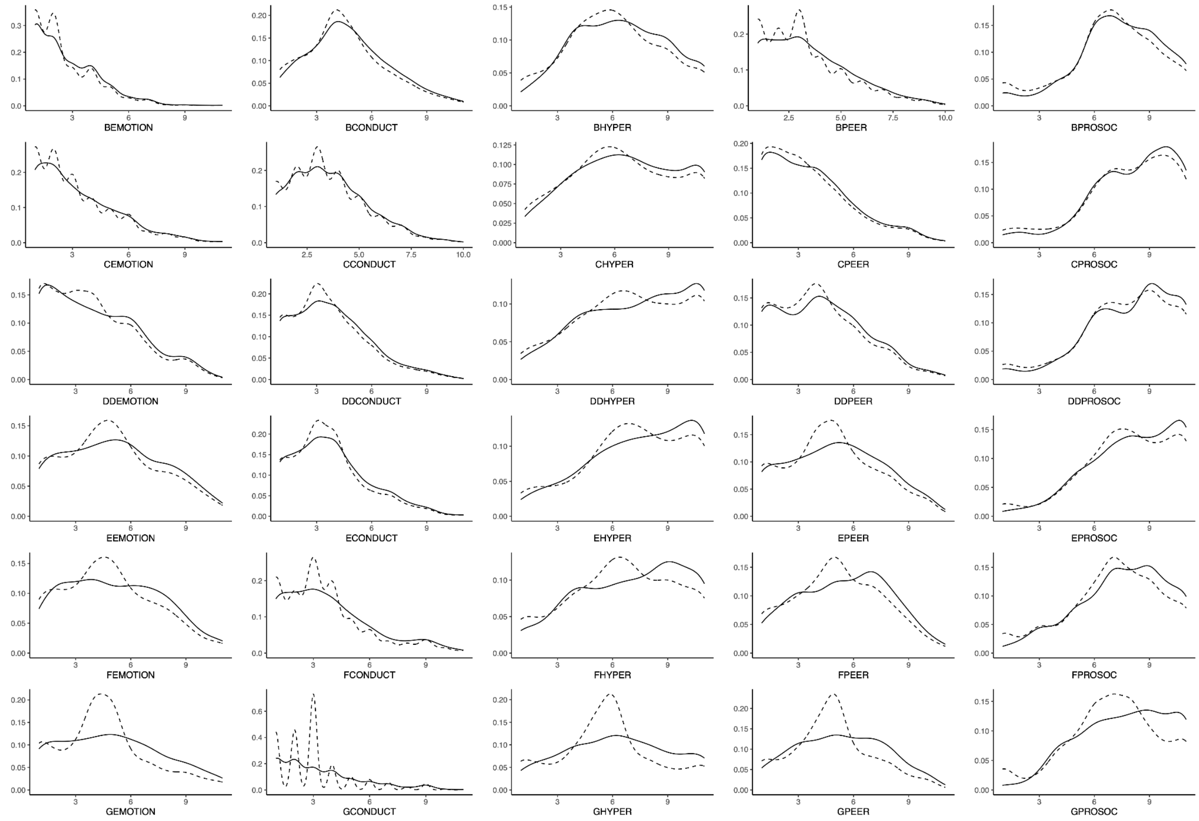

Note 2, Figure 1: Density plots of raw and imputed SDQ subscale scores for imputation quality evaluation. Solid lines represent original raw data, while dashed lines indicate imputed data. For each graph, the first letter represents the sweep using an ordinal coding system (e.g.,B = Sweep 2 at age 3, ..., G = Sweep 7 at age 17). EMOTION = Emotional Symptoms; CONDUCT = Conduct Problems; HYPER = Hyperactivity/Inattention; PEER = Peer Relationship Problems; PROSOC = Prosocial Behaviours.

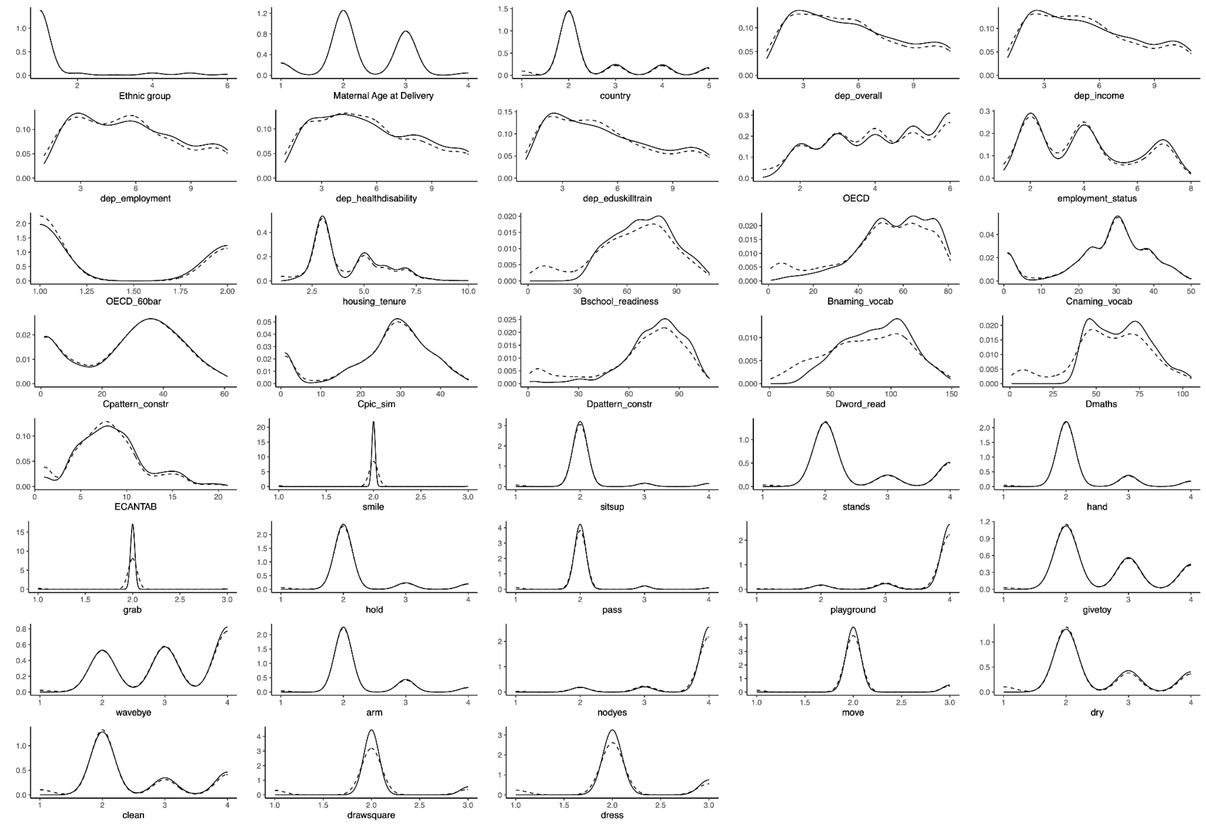

Note 2, Figure 2: Density plots of raw and imputed auxiliary variables for imputation quality evaluation. Solid lines represent original raw data, while dashed lines indicate imputed data.
dep_ = deprivation; OECD = Organisation for Economic Co-operation and Development score; OECD_60bar = OECD below 60% Poverty Indicator; naming_vocab = Naming Vocabulary; pattern_constr = Pattern Construction; pic_sim = Picture Similarity; word_read = Word Reading; CANTAB = Cambridge Neuropsychological Test Automated Battery Verbal Similarities Total Correct Responses. stands = stands up holding on; hands = puts hands together; grab = grabs objects; hold = holds small objects; pass = passes a toy; playground = Frequency take child to park or playground; givetoy = gives toy; wavebye = waves bye-bye arm = Extend his arms for being picked up; nodyes = nods for yes; move = can move from place to place; dry = child dry during the day; clean = child clean during the day; drawsquare = can draw or copy a square; dress = can dress without help. See details in **Supplementary Table 3**.

Note 3: Impact of various demographic and clinical characteristics on age at autism diagnosis

Age at autism diagnosis is complex and likely impacted by several socio-biological factors that vary across time and geography. Some of these factors can confound our results. We assessed the impact of six factors on our findings. These include: 1. Changes in diagnostic criteria over time; 2. Diagnostic misclassification; 3. Co-occurring intellectual disability and developmental delays in the child; 4. Country-level differences in diagnostic practices; 5. Sex; and 6. Parental factors influencing age at autism diagnosis.

Our primary aim was to investigate whether genetic and longitudinal results we obtained primarily reflect these six factors. We do not exclude the possibility of these factors impacting age at autism diagnosis through other mechanisms. Although we cannot say these factors do not contribute to some of the observed differences, we can say, through a series of analyses, that these factors are not the primary drivers of the results we have obtained.

1. Clinical factors: Changes in diagnostic criteria over time

The following cohorts primarily used an ICD based system for diagnosis: iPSYCH, MCS, GUI. The following cohorts primarily used a DSM based system for diagnosis: LSAC-B, LSAC-K, SPARK.

Details of when the birth cohorts were collected and the diagnostic changes that affect them are provided in **Supplementary Table 1**.

We considered the impact of the following changes to the diagnostic criteria in our analyses.

(1) DSM - III (1980) to DSM - IV (1994)

- Does not impact any of the longitudinal analyses as all the participants in the birth cohorts were born after the introduction of DSM - IV.
- To minimise the impact on the genetic analyses in SPARK[3] we restricted it to autistic individuals who were 22 years or younger, as these autistic individuals were born after 1994 (diagnosed using DSM - IV and DSM5).

(2) ICD - 9 (1977) to ICD - 10 (1994)

- Does not impact any of the longitudinal analyses as all the birth cohorts were born after the introduction of ICD - 10.
- To minimise the impact on the genetic analyses, in iPSYCH[4], we ran sensitivity analyses by conducting GWAS of autism diagnosed before age 9 ($iPSYCH_{before9}$) and after age 12 ($iPSYCH_{after11}$), and after restricting it to individuals born in 1994 or after. We then compared these results to the original GWAS ($iPSYCH_{before9}$ and $iPSYCH_{after11}$) that included all birth years. The genetic correlation between the GWAS with and without individuals born before 1994 was high ($r_g > 0.95$) and not statistically different from 1, indicating strong consistency in the genetic findings regardless of birth year restrictions.

(3) DSM - IV (1994) to DSM - 5 (2013)

- Affects diagnosis in the Australian cohorts (LSAC-K and LSAC-B). However, it does not affect diagnosis in MCS or GUI from the UK and Ireland respectively, where ICD was predominantly used. We observe similar results between the Australian cohorts and cohorts in Ireland and UK, suggesting that the results observed are not primarily due to changes in the diagnostic criteria, or differences between DSM and ICD.
- Does not affect iPSYCH analyses. However, in SPARK, approximately 60% of the participants included in the study were diagnosed after 2013, implying that they were diagnosed using the DSM-5 criteria. The remaining 40% were diagnosed using the DSM-IV criteria. The PGS for $iPSYCH_{after10}$ was associated with age at autism diagnosis in SPARK even after controlling for the DSM edition (DSM-IV vs DSM-5). Notably, we observed no significant interaction effect between $iPSYCH_{after10}$ PGS and DSM edition, suggesting that $iPSYCH_{after10}$ PGS has similar effects on age at autism diagnosis regardless of which DSM edition was used to diagnose autism.

(4) ICD - 10 (1994 in Denmark) to ICD - 11 (2018)

- This does not impact any of the longitudinal analyses as children in the MCS and GUI cohorts were diagnosed before ICD-11 has been used by the UK and Ireland respectively .
- Does not impact any genetic analyses as the iPSYCH participants were all diagnosed before 2015.

2. Clinical factors: Diagnostic misclassification

We wondered if later diagnosed autism reflects diagnostic misclassification of mental health conditions and ADHD, especially among adolescents and adults, where developmental history is difficult to ascertain[5]. This would indicate that later diagnosed autism would be a mixture of both “true autism” and other mental health and neurodevelopmental conditions diagnostically misclassified as autism. Consequently, in the context of our study, while the earlier diagnosed autism factor (and the GWAS contributing to the factor) would represent a “true” genetic signal for autism, the later diagnosed autism factor represented a mixture of genetic signals from autism and other mental health and neurodevelopmental phenotypes.

We tested if later diagnosed autism can be completely explained by diagnostic misclassification using genomicSEM.

To investigate this, we assumed that the PGC-2017 autism GWAS[6] was a “gold-standard” GWAS of autism as autistic participants in this GWAS typically underwent rigorous assessment using tools such as ADOS[7] and ADI-R[8]. We further assumed that autistic individuals included in the iPSYCH GWAS consisted of a fraction of autistic individuals with a true autism diagnosis and individuals who were diagnostically misclassified as autistic. If this were true then the total genetic variance of the iPSYCH autism GWAS could be explained by the PGC-2017 autism GWAS and other mental health conditions. In other words, the residual genetic variation would be statistically nonsignificant.

We tested this using genomicSEM after regressing the genetic effects of the iPSYCH GWAS simultaneously on the genetic effects of PGC-2017 autism GWAS[6], ADHD[9], depression[10], schizophrenia[11], bipolar disorder[12], anorexia nervosa[13], and PTSD[14] (**Note 3, Figure 1**).

The residual genetic variance for iPSYCH autism GWAS was 0.37 (s.e.m = 0.10, P = $2.97x10^{-4}$). This suggests that just under 40% of the genetic variance in the iPSYCH autism was not explained by either the PGC-2017 autism GWAS or other mental health conditions. In the multiple regression framework within genomicSEM, we observed significant genetic correlation between the iPSYCH autism GWAS and the PGC-2017 autism GWAS (r_g = 0.59, s.e.m = 0.12, P = $1.64x10^{-6}$) and the ADHD GWAS (r_g = 0.31, s.e.m = 0.10, P = $1.80x10^{-3}$). We observed no significant genetic correlation between the iPSYCH autism GWAS and any of the other GWAS. Additionally, the genetic correlation between the iPSYCH and PGC-2017 without conditioning on the other GWAS was (r_g = 0.61, s.e.m = 0.10, P = $2.64x10^{-9}$). The genetic correlation between the PGC-2017 and iPSYCH autism GWAS were statistically similar before and after conditioning on the genetic effects of other mental health phenotypes.

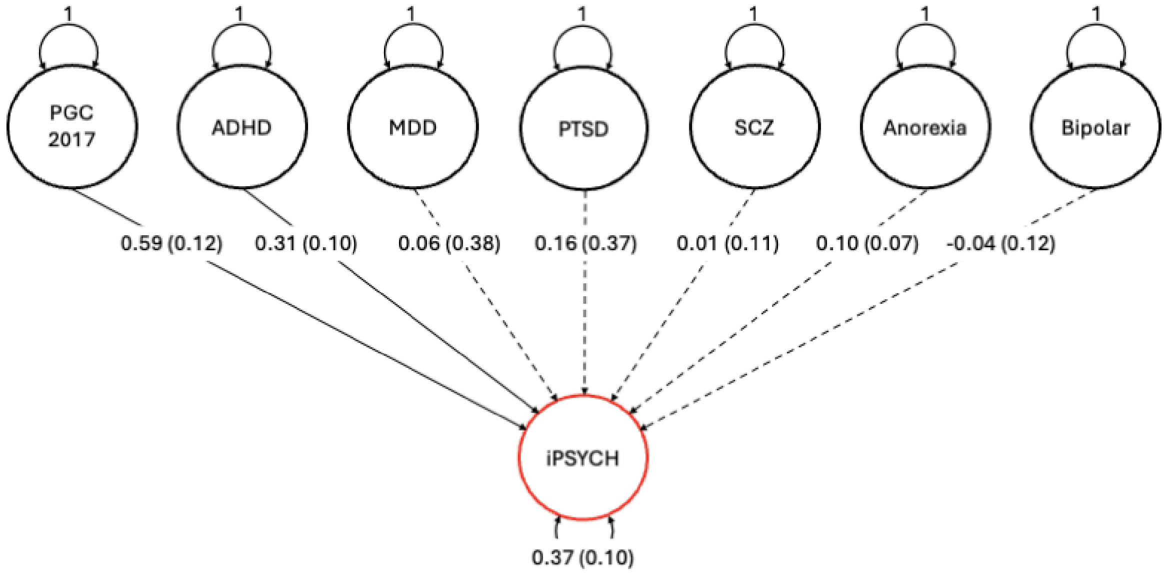

Note 3, Figure 1: Path diagrams representing results from genomic multiple regression analyses using genomicSEM. The genetic effects of the iPSYCH autism GWAS were regressed on the genetic effects of the PGC-2017 autism GWAS, attention-deficit/hyperactivity disorder (ADHD), major depressive disorder (MDD), posttraumatic stress disorder (PTSD), schizophrenia (SCZ), anorexia nervosa (Anorexia), and bipolar disorder (Bipolar) simultaneously. Single-headed arrows indicate conditional genetic associations between the explanatory variables and the iPSYCH autism GWAS. Numbers represent standardised correlation coefficients, with standard errors in parentheses. Genetic associations between explanatory factors were accounted for in the analyses but are not shown on the graph for simplicity. The two-headed arrows connecting the genetic component of the iPSYCH autism GWAS to itself represent the residual genetic variance unexplained by the genetic influence of either the PGC-2017 autism GWAS or other mental health conditions. Solid lines indicate significant genetic associations, while dashed lines indicate non-significant associations.

As the PGC-2019 autism GWAS indexes earlier diagnosed autism, we reasoned that the residual genetic variance in iPSYCH autism GWAS may be partly explained by an index of later diagnosed autism. We used the independent FinnGen GWAS as an index of later diagnosed autism. Subsequently, we reconducted the genomicSEM analyses using the multiple regression framework with the additional inclusion of the FinnGen autism GWAS (**Note 3, Figure 2**). In this model, the residual genetic variance of the iPSYCH autism GWAS was not statistically significant (r_g = 0.21, s.e.m = 0.15, P = 0.16). In this model, the iPSYCH autism GWAS had statistically significant genetic correlations with the PGC-2017 autism GWAS (r_g = 0.53, s.em = 0.13, P = 8.81×10^{-5}), ADHD (r_g = 0.33, s.e.m = 0.11, P = 4.23×10^{-3}), and with the FinnGen autism GWAS (r_g = 0.43, s.e.m = 0.20, P = 3.83×10^{-2}). There were no statistically significant genetic correlation among the ADHD, FinnGen autism, and PGC-2017 autism GWAS.

The PGC-2017 autism GWAS explained 39% of the genetic variance in iPSYCH autism, followed by FinnGen which explained an additional 21% of the genetic variance, and finally, ADHD explained an additional 17% of the genetic variance.

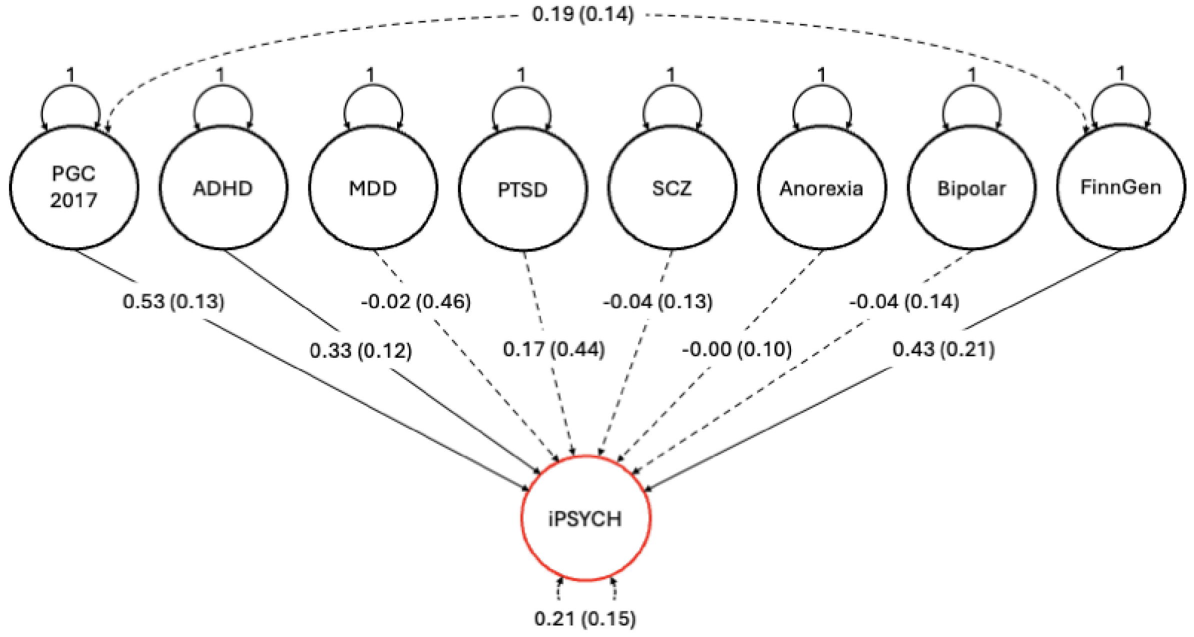

Note 3, Figure 2: Path diagrams representing results from genomic multiple regression analyses using genomicSEM. The genetic effects of the iPSYCH autism GWAS were regressed simultaneously on the genetic effects of the PGC-2017 autism GWAS, and other neurodevelopmental and mental health conditions, as well as FinnGen GWAS. Single-headed arrows indicate conditional genetic associations between the explanatory variables and the iPSYCH autism GWAS. Numbers represent standardised correlation coefficients, with standard errors in parentheses. Genetic correlation between the PGC autism GWAS and FinnGen GWAS are shown with a two-headed arrow connecting them. Other genetic associations between explanatory variables are omitted for readability. The two-headed arrows connecting the genetic component of the iPSYCH autism GWAS to itself represent the residual genetic variance unexplained by the genetic influence of either the PGC-2017 autism GWAS or other mental health conditions. ADHD, attention-deficit/hyperactivity disorder; MDD, major depressive disorder; PTSD, posttraumatic stress disorder; SCZ, schizophrenia; Anorexia, anorexia nervosa; Bipolar, bipolar disorder. Solid lines indicate significant genetic associations, while dashed lines indicate non-significant associations.

Taken together, we find no evidence that the later diagnosed autism is explained by diagnostic misclassification of schizophrenia, anorexia, depression, PTSD, or bipolar. We find modest shared genetics between later diagnosed autism and ADHD, suggesting that a fraction of the genetic signal of later diagnosed autism may be due to diagnostic misclassification of ADHD as autism (**Note 3, Figure 3**). However, this does not exclude the possibility that this could merely reflect shared genetics between ADHD and later diagnosed autism. Further confirming the contribution of late diagnosed autism, we identified a significant genetic correlation between the FinnGen autism GWAS and the iPSYCH autism GWAS after conditioning on the genetic effects of the childhood diagnosed autism (PGC-2017), ADHD, and other mental health conditions.

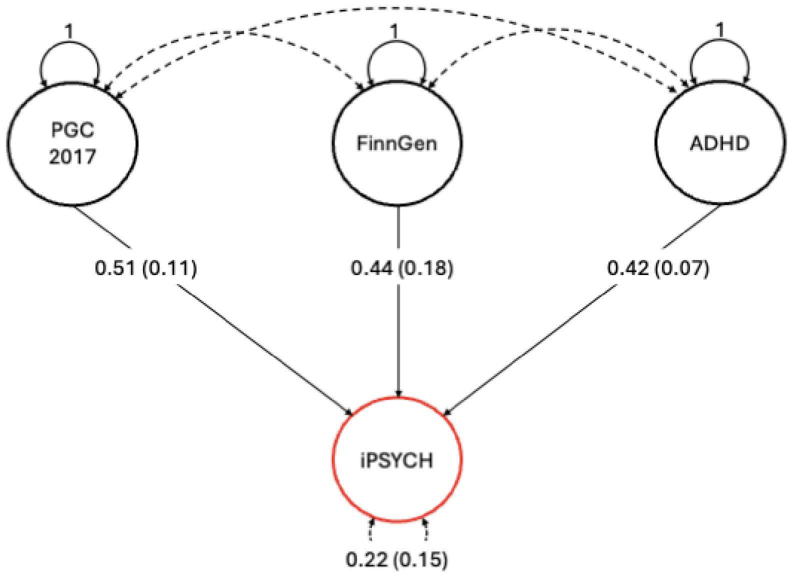

Note 3, Figure 3: Path diagrams representing results from genomic multiple regression analyses using genomicSEM. The genetic effects of the iPSYCH autism GWAS were regressed on the genetic effects of the PGC-2017 autism GWAS, and FinnGen GWAS simultaneously. Single-headed arrows indicate conditional genetic associations between the explanatory variables and the iPSYCH autism GWAS. Numbers represent standardised correlation coefficients, with standard errors in parentheses. Genetic associations between the PGC autism GWAS and FinnGen GWAS are shown with a two-headed arrow connecting them. The two-headed arrows connecting the genetic component of the iPSYCH autism GWAS to itself represent the residual genetic variance unexplained by the genetic influence of either the PGC-2017 autism GWAS or other mental health conditions. Solid lines indicate significant genetic associations, while dashed lines indicate non-significant associations.

3. Clinical factors: Intellectual disability and co-occurring developmental delays

Several sensitivity analyses indicate that the findings are not driven by co-occurring intellectual disability (ID) and developmental delays.

- In longitudinal analyses of birth cohorts, none of the autistic participants had ID. Furthermore, in regression models, the first principal component of a child's cognitive aptitude does not explain a significant proportion of the variance in age at autism diagnosis.

- In SPARK, we observed no significant attenuation of the SNP-based heritability in age at autism diagnosis when accounting for co-occurring ID, and age at attaining developmental milestones.
- In SPARK, we find similar over-transmission of the polygenic scores (PGS) for $iPSYCH_{before11}$ and $iPSYCH_{after10}$ among autistic individuals with and without ID.
- In SPARK, PGS for $iPSYCH_{after10}$ has a similar positive association with age at autism diagnosis even after excluding individuals with ID and limited verbal ability (**Supplementary Table 15**).

4. Clinical factors: Country/continent level differences in diagnosis

Our analyses indicate that the results do not primarily reflect differences in diagnostic practices across countries.

- In the longitudinal analyses of birth cohorts, we find similar results in cohorts from the UK, Ireland, and Australia. Notably, the UK uses the ICD, and Australia primarily uses the DSM.
- The pattern of genetic correlation among different autism GWAS was not explained by country level/cohort differences. For example, $iPSYCH_{before9}$ had significantly higher genetic correlation with the PGC-2017 (r_g = 0.90, s.e.m = 0.09) than with $iPSYCH_{after11}$ (r_g = 0.70, s.e.m = 0.05). Similarly, the $SPARK_{after10}$ had higher genetic correlation with $iPSYCH_{after10}$ (r_g = 0.83, s.e.m = 0.17) than with $SPARK_{before6}$ (r_g = 0.31, s.e.m = 0.19).
- We further tested this using genomicSEM. In the genomicSEM analyses, a geography model wherein we tested the hypothesis that cohorts from Europe will load onto one factor and cohorts predominantly from North America will load onto a second factor had poor fit statistics.

5. Demographic factors: Sex

We ran several sensitivity analyses to confirm that the results were not primarily picking up sex differences.

- In the longitudinal analyses of birth cohorts, both latent growth curve models and GMM analyses conducted only in autistic males identified consistent results to the analyses conducted in both males and females.
- In the latent growth curve analyses, age of diagnosis stratified models better fit the data compared to sex-stratified models.
- In birth cohorts, multiple regression and mediation analyses indicated that trajectories explained a larger proportion of the variance in age at autism diagnosis than sex.
- In genetic analyses, we find a significant SNP-based heritability for age at autism diagnosis even after accounting for sex as a covariate.
- PGS for $iPSYCH_{after11}$ is associated with later autism diagnosis in both autistic males and females in the SPARK cohort.
- The pattern of genetic correlation between age at autism diagnosis and the various GWAS did not align well with the sex ratios in the various GWAS. For example, age at autism diagnosis in SPARK had a higher positive genetic correlation with iPSYCH males-only autism GWAS (r_g = 0.05, s.e.m = 0.10) compared to PGC-2019 (r_g = -0.67, s.e.m = 0.12). Given that PGC-2019 had a greater proportion autistic females than the iPSYCH males-only autism GWAS, these findings are incongruous with the hypothesis that the age of diagnosis GWAS is picking up sex differences.

- The pattern of genetic correlations among the different autism GWAS was not explained by sex differences. For example, the female-stratified autism GWAS in iPSYCH had higher genetic correlation with the male-stratified autism GWAS (r_g = 0.80, s.e.m = 0.08) than with the sex-unstratified PGC-2017 (r_g = 0.48, s.e.m = 0.12) or the SPARK autism GWAS (r_g = 0.50, s.e.m = 0.12).

6. Demographic factors: Parental characteristics

We ran a few analyses to understand if parental characteristics can impact age at autism diagnosis, primarily through gene-environment correlations.

- In the longitudinal analyses of birth cohorts, controlling for ethnic minority status[15], parental socio-economic status, material deprivation, and maternal age at birth does not impact the variance explained by the GMM latent classes on age at autism diagnosis (**Supplementary Table 25**).
- In SPARK, controlling for parental socio-economic status and neighbourhood deprivation does not significantly attenuate the SNP heritability for age at autism diagnosis.

Note 4: GMM river plots and explanation

In the Growth Mixture Model analyses in MCS-expanded and MCS-imputed, the optimal GMM identified more than two latent trajectory classes in some SDQ subscales (**Supplementary Tables 3 and 5**).

River plots (**Note 4, Figures 1 - 8**) tracking changes in trajectory latent class memberships between the two-class model and the optimal model (three or four latent) for different subscales show that the additional latent classes were predominantly subsets of the original two latent classes. These are indicative that although two broad trajectories remain even with expanded sample sizes, these can be further decomposed into smaller groups with different trajectories.

For instance, in the GMM analyses of the SDQ Conduct problems subscale on MCS-expanded, the two-group model identified two trajectories: one with stably decreasing conduct problems (green latent class) and another with accelerating conduct problems, particularly after age 7 (purple latent class) (**Note 4, Figure 1A**). The optimal three-trajectory model further refined these trajectories. It identified a consistently low conduct problems group, largely composed of individuals from the green class in the two-trajectory model (**Note 4, Figure 3A**). Children in the late childhood emergent group predominantly remained in the overall increasing class (purple in both models). Stacked bar charts indicated that more children in the excessive increasing groups (purple) were diagnosed at or after age 7 in both models (**Note 4, Figure 1**). Additionally, a distinct third group with initially high but progressively decreasing conduct problems was identified; their symptoms declined to become the second-lowest (blue latent class) (**Note 4, Figure 1B**). With their aligned decreasing trend, this group appeared to be a subset of the generally decreasing trajectory group (green class in the two-trajectory model) (**Note 4, Figure 3A**).

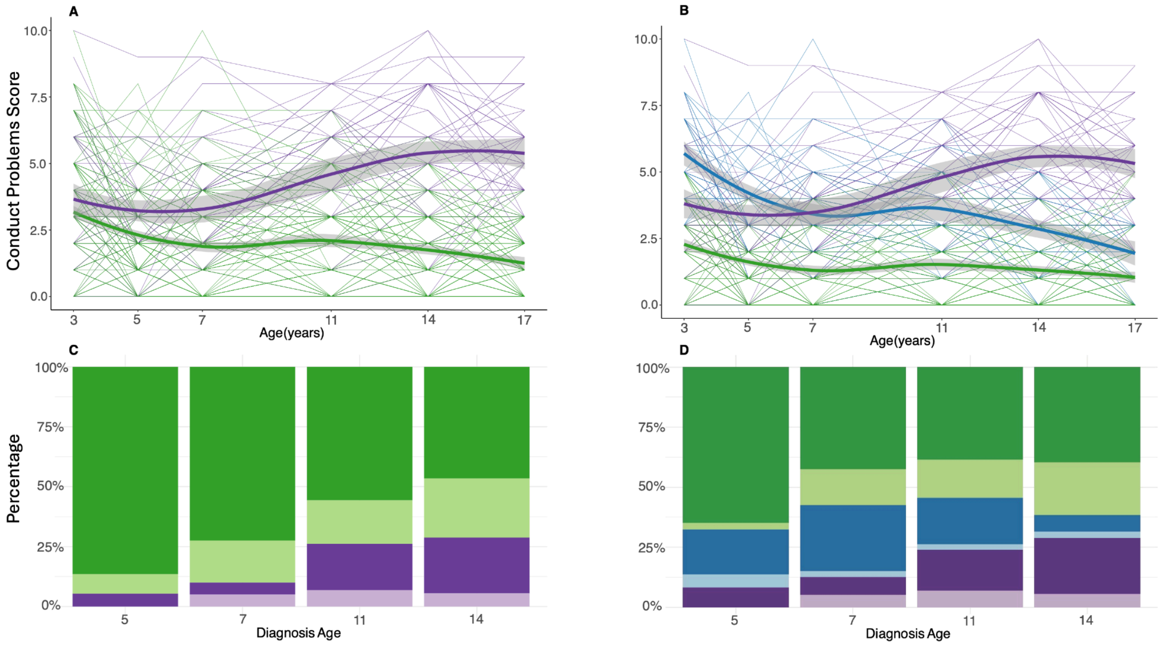

Note 4, Figure 1: A: Two-group longitudinal growth mixture model of total SDQ Conduct Problems score among autistic individuals in the MCS-expanded. B: The optimal longitudinal growth mixture model of total SDQ Conduct Problems score among autistic individuals in MCS-expanded, demonstrating three latent trajectories. C-D: Stacked bar charts providing proportion of individuals who had been diagnosed as autistic at specific ages by the latent classes membership from the corresponding growth mixture models. Darker colours indicate males and lighter colours indicate females.

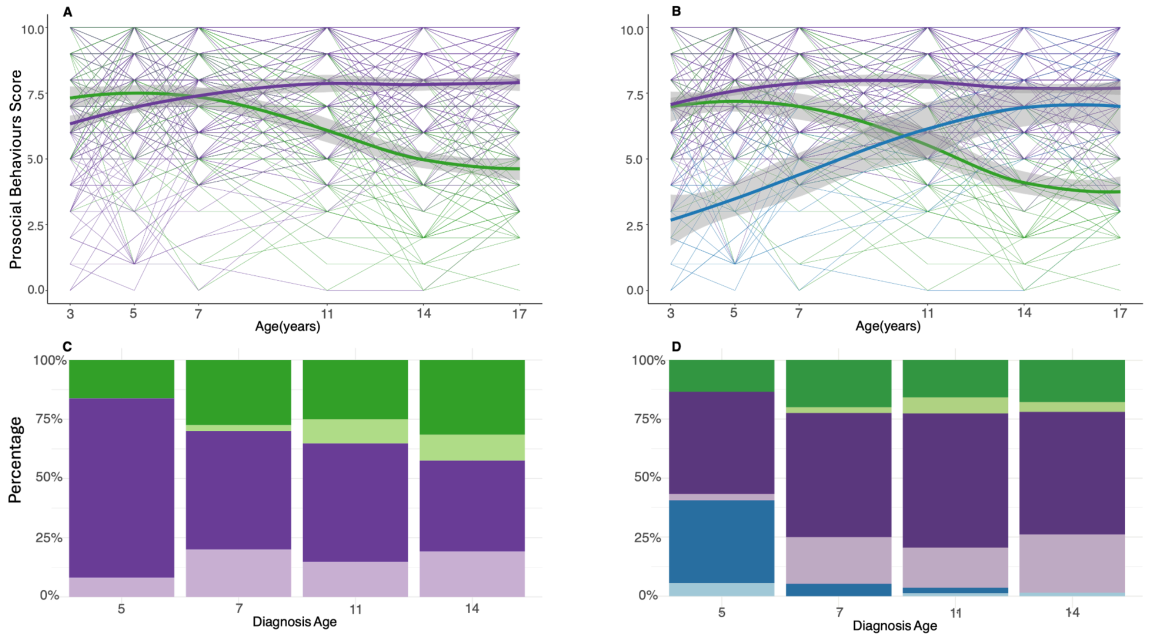

Note 4, Figure 2: A: Two-group longitudinal growth mixture model of total SDQ Prosocial Behaviours score among autistic individuals in MCS-expanded. B: The optimal longitudinal growth mixture model of total SDQ Conduct Problems score among autistic individuals in MCS-expanded, demonstrating three latent trajectories. C-D: Stacked bar charts providing proportion of individuals who had been diagnosed as autistic at specific ages by the latent classes membership from the corresponding growth mixture models. Darker colours indicate males and lighter colours indicate females.

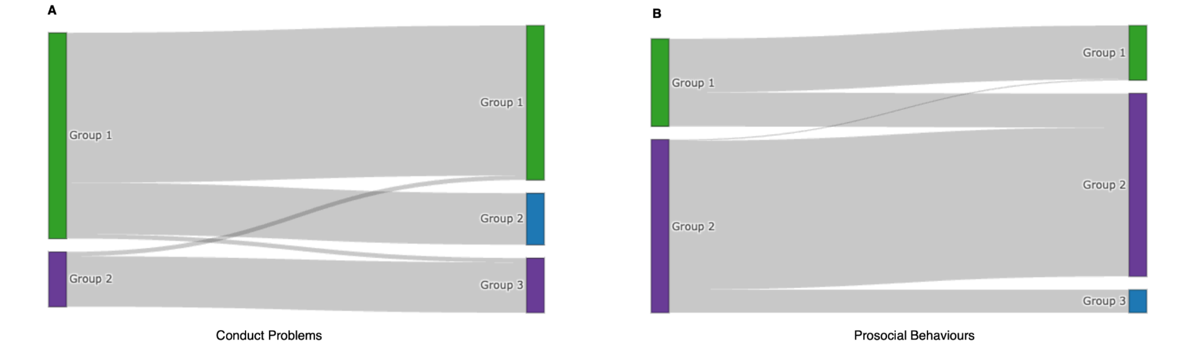

Note 4, Figure 3: River plots (Sankey Plots) of Conduct Problems (A) and Prosocial Behaviours (B) for expanded MCS, illustrating the changes in latent trajectory group memberships between the two-group GMM and the optimal three-group GMM in the MCS-expanded. Colours are consistent with Note 4, Figures 1 and 2.

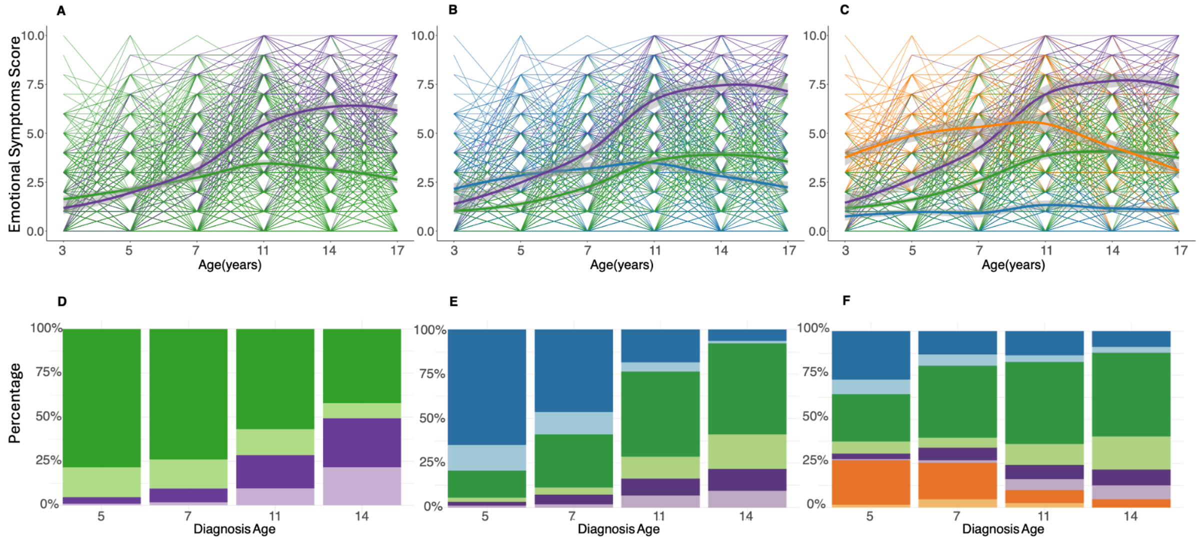

Note 4, Figure 4: A: Two-group longitudinal growth mixture model of total SDQ Emotional Symptoms score among autistic individuals in the MCS-imputed. B: Three-group longitudinal growth mixture model of total SDQ Emotional Symptoms score among autistic individuals in MCS-imputed C: The optimal longitudinal growth mixture model of total SDQ Emotional Symptoms score among autistic individuals in MCS-imputed, demonstrating four latent trajectories. D-F: Stacked bar charts providing proportion of individuals who had been diagnosed as autistic at specific ages by the latent classes membership from the corresponding growth mixture models. Darker colours indicate males and lighter colours indicate females.

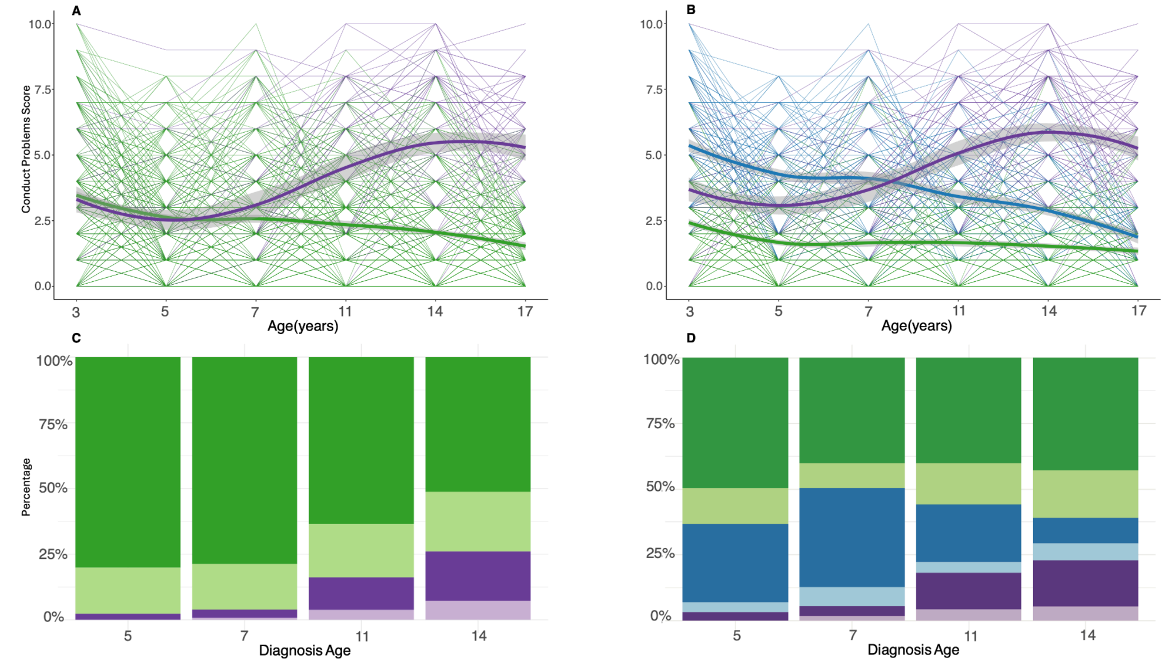

Note 4, Figure 5: A: Two-group longitudinal growth mixture model of total SDQ Conduct Problems score among autistic individuals in MCS-imputed B: The optimal longitudinal growth mixture model of total SDQ Conduct Problems score among autistic individuals in MCS-imputed, demonstrating three latent trajectories. C-D: Stacked bar charts providing proportion of individuals who had been diagnosed as autistic at specific ages by the latent classes membership from the corresponding growth mixture models. Darker colours indicate males and lighter colours indicate females.

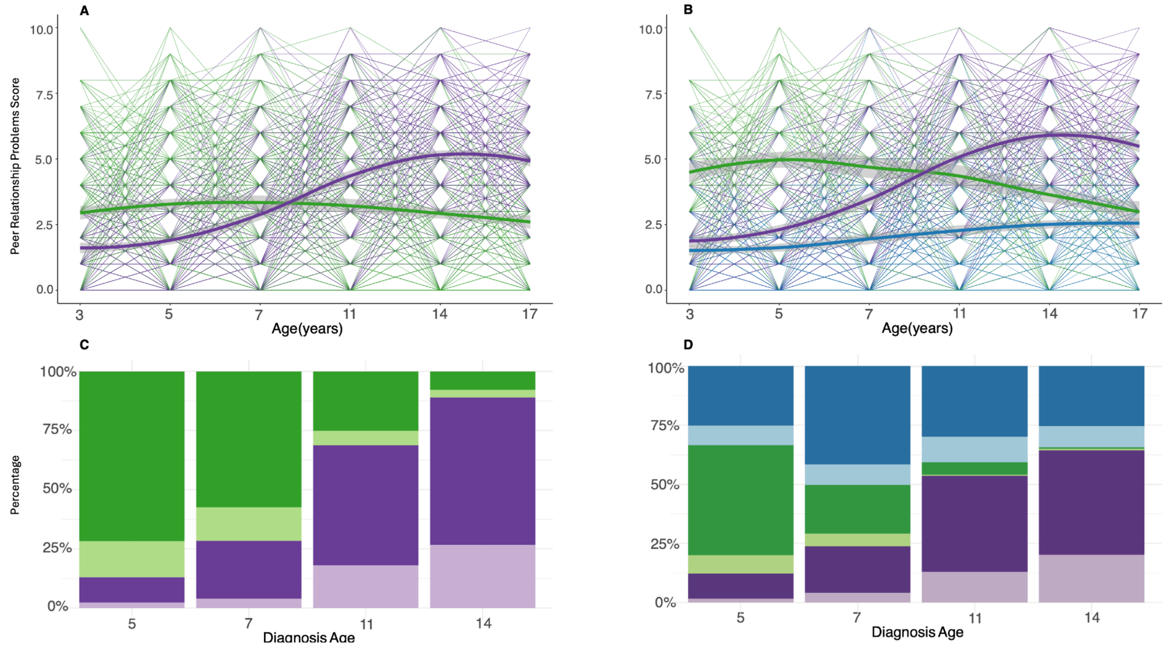

Note 4, Figure 6: A: Two-group longitudinal growth mixture model of total SDQ Peer Relationship Problems score among autistic individuals in MCS-imputed. B: The optimal longitudinal growth mixture model of total SDQ Peer Relationship Problems score among autistic individuals in the MCS-imputed, demonstrating three latent trajectories. C-D: Stacked bar charts providing proportion of individuals who had been diagnosed as autistic at specific ages by the latent classes membership from the corresponding growth mixture models. Darker colours indicate males and lighter colours indicate females.

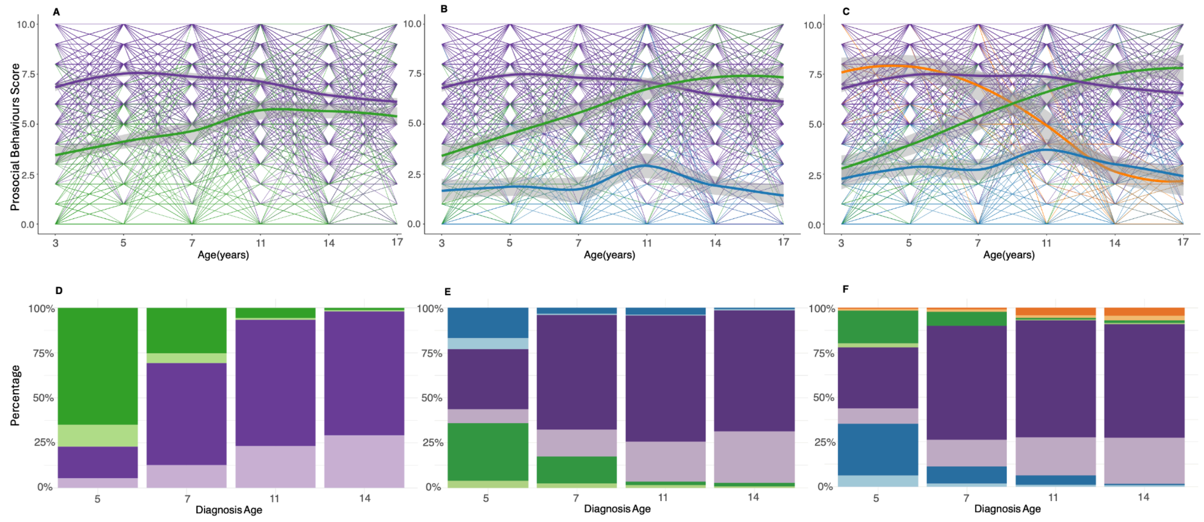

Note 4, Figure 7: A: Two-group longitudinal growth mixture model of total SDQ Prosocial Behaviours score among autistic individuals in MCS-imputed. B: Three-group longitudinal growth mixture model of total SDQ Prosocial Behaviours score among autistic individuals in MCS-imputed. C: The optimal longitudinal growth mixture model of total SDQ Prosocial Behaviours score among autistic individuals in MCS-imputed, demonstrating four latent trajectories. D-F: Stacked bar charts providing proportion of individuals who had been diagnosed as autistic at specific ages by the latent classes membership from the corresponding growth mixture models. Darker colours indicate males and lighter colours indicate females.

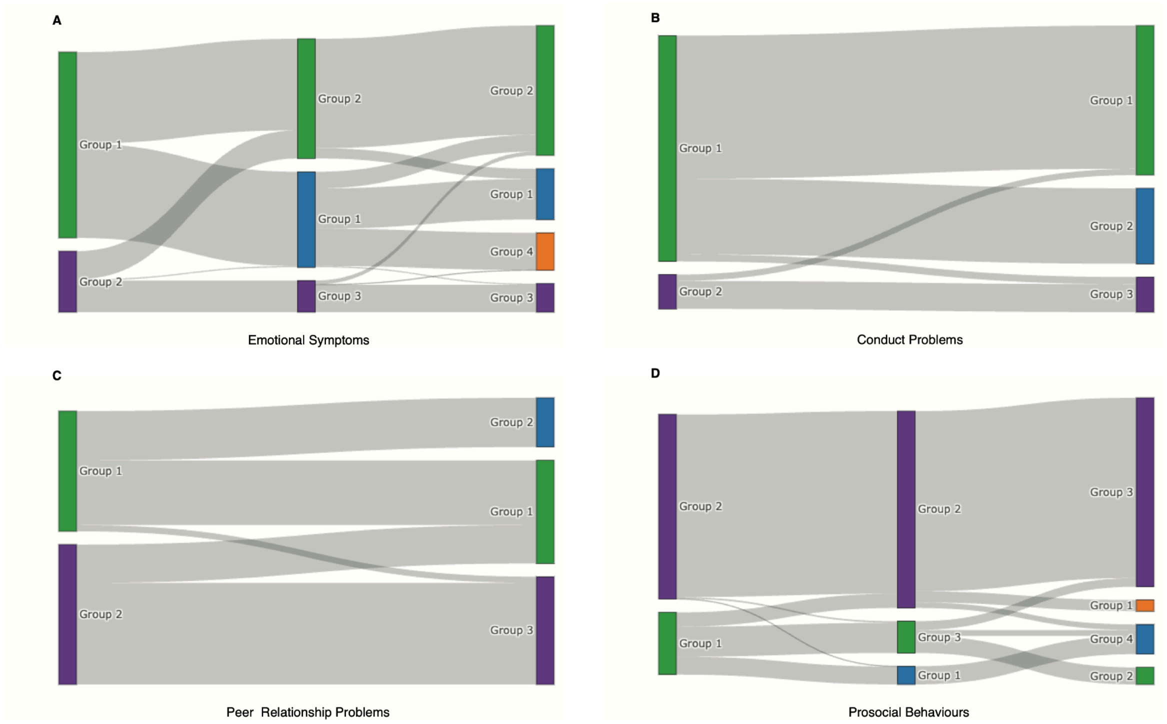

Note 4, Figure 8: River plots (Sankey Plots) of Emotional Symptoms (A), Conduct Problems (B), Peer Relationship Problems (C) and Prosocial Behaviours (D) for MCS-imputed, illustrating changes in latent trajectory group memberships between the two-group GMM and the optimal GMM (three or four groups identified). Colours are in alignment with Note 4, Figure 4-7.

Note 5: Mediation analyses

In multiple regression analysis, there were relatively modest effects of sex and other socio-demographic variables on age at autism diagnosis. Also, as discussed above in Note3, socio-demographic factors such as socio-economic statuses (SES) or ethnicities may have impacts on developmental profiles (as reflected in latent class identified in GMMs), which could in turn affect age at diagnosis. To test this potential causal relationship, we conducted a mediation analysis with the latent classes serving as mediators. Specifically, using structural equation modelling, we modelled the direct effects of latent classes and socio-demographic variables on age at diagnosis of autism, as well as indirect effects through affecting latent class memberships of SDQ total and subscale scores. Utilising the *lavaan* package (v. 0.6.17) in R, we adhered to the methodology outlined in ref[16], to assess the strength and significance of four effect sets: specific indirect, total indirect, direct, and total effects (**Supplementary Table 8**). We employed bootstrap analysis, a nonparametric sampling procedure, to ascertain the significance of the indirect effects.

However, most socio-demographic variables did not have significant direct or indirect effects, underscoring the small effect sizes of these factors in explaining the age at autism diagnosis in our samples. Overall, results of both multiple regression and mediation analysis suggest that neurodevelopmental trajectories explain a relatively large proportion of variance in age at autism diagnosis (**Supplementary Table 8**). Consistent results were obtained for the MCS-expanded and MCS-imputed. In ADHD, neurodevelopmental trajectories and other socio-demographic factors did not have a significant impact on age at ADHD diagnosis, explaining a much smaller proportion of variation compared to autism. The results were primarily driven by the hyperactivity/inattention and peer relationship problems subscales, demonstrating relative specificity of the findings to autism (**Supplementary Table 4**).

Note 6: Sensitivity analyses of the genetic correlation results

We conducted genetic correlation analyses between individual age of diagnosis stratified autism GWAS in iPSYCH and SPARK and cognitive, psychiatric and neurodevelopmental phenotypes. These results are provided in **Note 6, Figure 1**. We note consistent results between iPSYCH and SPARK for all phenotypes except for educational attainment and cognitive aptitude. The possible differences in SPARK may be attributed to participation bias - more educated individuals may be more likely to both pursue an autism diagnosis and participate in a study like SPARK.

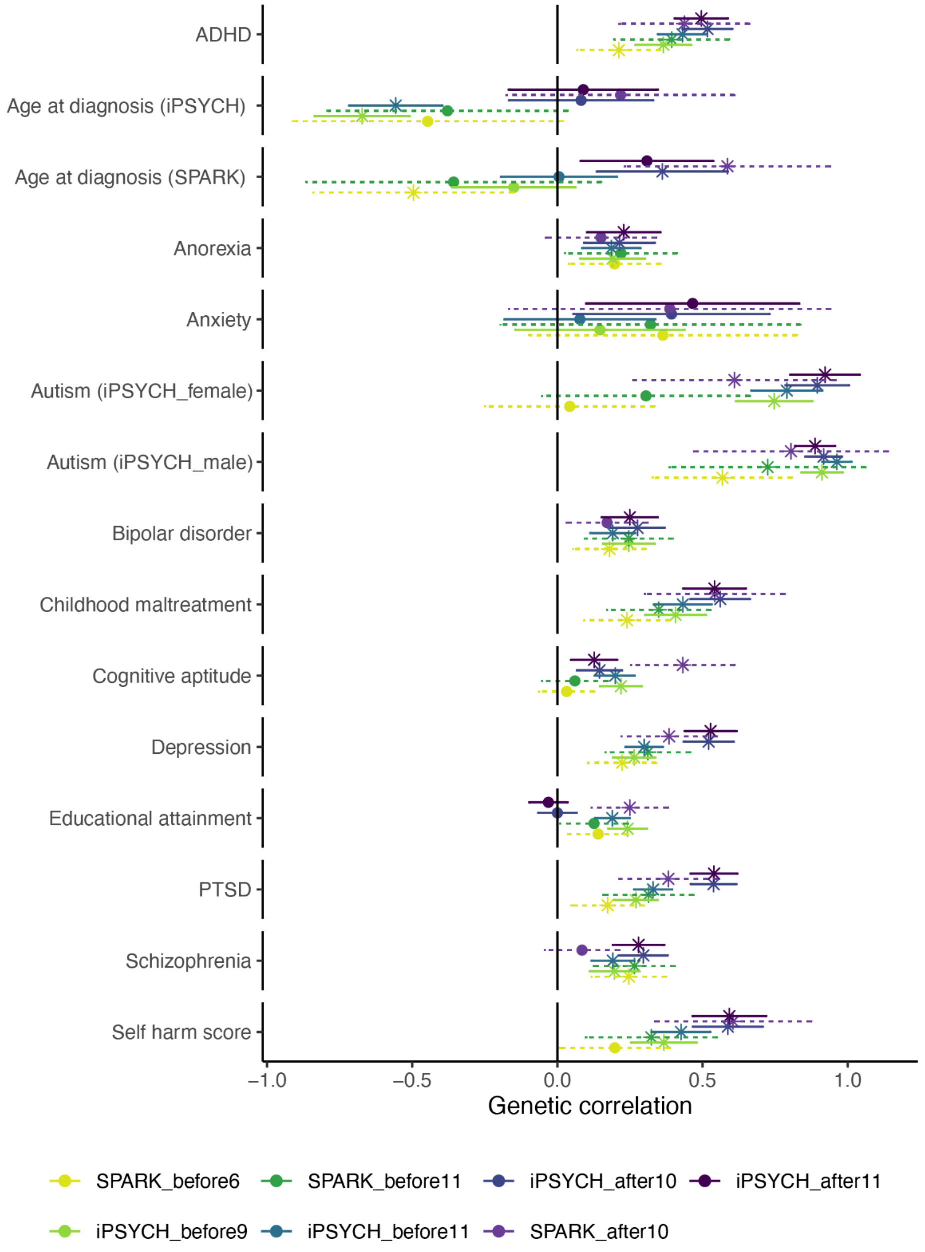

Note 6, Figure 1: Genetic correlation between age at diagnosis stratified autism GWAS and other mental health, neurodevelopmental, and cognition related traits. Points indicate the estimate, whiskers indicate 95% confidence intervals, and points with asterisk (*) indicates significant associations with Benjamini-Yekutieli adjustment. Solid lines represent GWAS from iPSYCH. Dotted lines represent GWAS from SPARK.

We also used genomicSEM to investigate if the association between iPSYCH$_{after10}$ and mental health phenotypes is attenuated after conditioning on the genetic effects of ADHD.

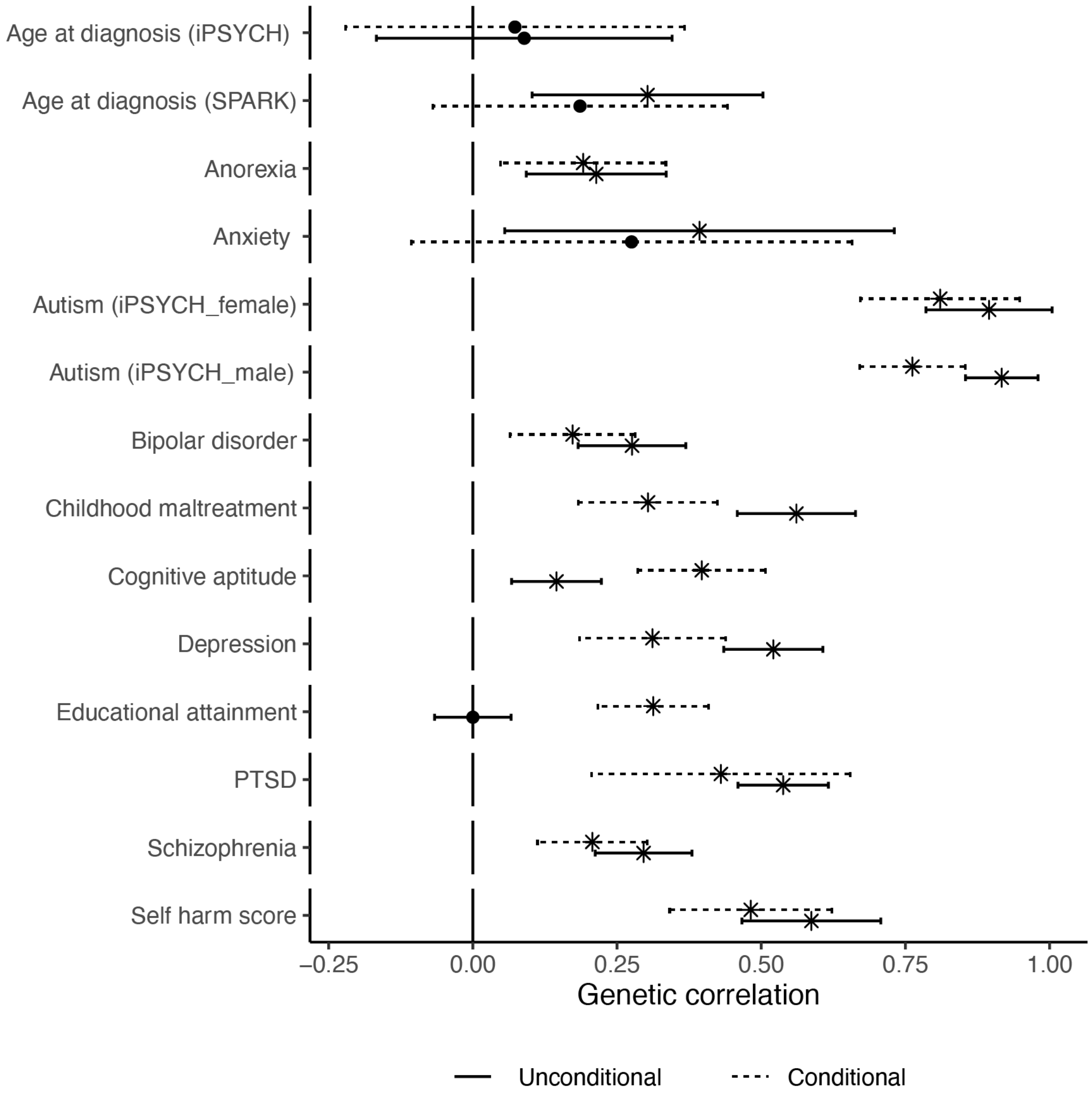

Note 6, Figure 2: Genetic correlation between iPSYCH$_{after10}$ autism GWAS and other traits before and after conditioning on the genetic effects of ADHD. Points indicate the estimate, whiskers indicate 95% confidence intervals, and points with an asterisk (*) indicate significant associations after Benjamini-Yekutieli adjustment. Solid lines represent genetic correlation estimates from iPSYCH$_{after10}$ that have not been conditioned on the genetic effects of ADHD. Dotted lines represent genetic correlation estimates from iPSYCH$_{after10}$ after conditioning on the genetic effects of ADHD

Summary

We wanted to understand why some autistic people receive a diagnosis only in late childhood or afterwards. We knew, from previous research, that one reason for variable age at autism diagnosis is because behavioural features that typically lead to an autism diagnosis change from infancy to adolescence.

To better understand how changes in behavioural features can impact when someone receives an autism diagnosis, we analysed data from four long-term studies that followed children from birth through adolescence. In all four studies, children were born around the same time. We measured behavioural features using a parent/caregiver reported questionnaire called the Strengths and Difficulties Questionnaire, measured at multiple time points as the children grew up.

We found that autistic children tended to follow one of two different behavioural trajectories as they grew up. One group showed higher behavioural difficulties from a very young age that remained relatively stable over time. Children in this group were more likely to be diagnosed with autism at an earlier age.

The other group did not show as many difficulties in early childhood, but began to struggle more with social skills, emotional problems, and peer relationships in late childhood and adolescence. Those in this second group were more commonly diagnosed with autism later, often in adolescence.

We then looked at genetic factors and found that a person's genetic profile correlates with their age of autism diagnosis. We identified two correlated genetic profiles, or "polygenic factors", associated with autism that seem to correspond to the two behavioural trajectories.

One genetic factor was linked to being diagnosed earlier and having more social communication difficulties in infancy. The other genetic factor was associated with a later autism diagnosis, more emotional and peer problems in adolescence, and higher rates of other conditions like ADHD and trauma.

These findings suggest there are different genetic influences that can predispose some people to show clear autism traits from a very young age, leading to an earlier diagnosis. For others, genetic influences may alter which autism features emerge and when. Some of these children may have features that are not picked up by parents or caregivers until they cause significant distress in late childhood or adolescence. These children seem to navigate childhood reasonably typically, but once faced with the complex social demands of adolescence, autism characteristics emerge more prominently at that point.

Our study suggests that the age when someone is diagnosed with autism seems to depend on a combination of their genetic factors influencing developmental trajectories, socio-behavioural challenges emerging at different points, and other factors still to be fully understood. Recognising this diversity and variability in autism can help more timely diagnosis and provide more personalised support for autistic people.

FAQs

About the Study

Why did you do this study?
We wanted to understand why some autistic people receive a diagnosis only in late childhood or later. We knew that there is some epidemiological and some qualitative studies on the topic. In parallel, we were also aware of studies that show that social, emotional, and behavioural features change over the course of one's life, especially during childhood and adolescence. Parents and caregivers typically seek an autism diagnosis when they observe social, emotional, and behavioural features in their children that need support. We wondered if changes in these features during early life partly contribute to when someone receives an autism diagnosis. Given that how social, emotional, and behavioural features change over a young person’s life is partly due to their genetics, we also wanted to understand how genetic variants can impact when these features emerge, leading to an autism diagnosis.

Simply put, the model we studied was this:

Genetic variants → Variable developmental trajectories over time → Variable emergence of autism features → Variable age at autism diagnosis

What did you find?

The main findings of this study are:

- There are at least two behavioural trajectories that autistic children tend to follow - one group shows elevated social, emotional, and behavioural difficulties from very early childhood, while the other group does not show as many issues until late childhood or adolescence.
- A person's age of being diagnosed with autism is partly explained by genetic factors and their neurodevelopmental trajectories. However, we still do not know many factors that explain when someone receives an autism diagnosis.
- We identified two correlated genetic profiles or "polygenic factors" that correspond to the two behavioural trajectories. One is linked to earlier autism diagnosis, social communication difficulties in infancy, and higher cognitive abilities. The other is associated with later diagnosis, increasing emotional/peer problems in adolescence, and higher rates of co-occurring conditions like ADHD and PTSD.

What are the implications of this study?
The findings suggest there is diversity in how autism manifests and emerges across childhood and adolescence, influenced by a person's unique genetic makeup. This has implications for:

- Improving our understanding of the diversity and variability seen in autism.
- Recognising that there is no one route to diagnosing autism. Multiple developmental pathways, some of which may fully emerge only later in childhood, can lead to an autism diagnosis.

- The need for personalised support and intervention approaches tailored to different developmental trajectories.
- Considering age of autism diagnosis as a factor in research on sex differences, co-occurring conditions, etc. in autism.

Why did you run genetic analyses?
We ran genetic analyses for a few key reasons:

- The behavioural trajectory data suggested that age of autism diagnosis is influenced by developmental factors. Since many developmental traits have a genetic component, we wanted to test if age of diagnosis itself is partly influenced by their genetics.
- We further sought to understand what specific genetic influences underlying age at autism diagnosis are shared with different behavioural trajectories and developmental milestones in the general population.
- We wanted to understand if studying the genetics of age at autism diagnosis could help explain some of the heterogeneity and diversity seen in the autism genetics studies, including the differential shared genetics with mental health conditions.
- Finally, genetic methods help to provide additional support to results observed from studying the changes in behavioural trajectories among autistic individuals in four long-term bi.

What genetic analyses did you run?
We used a few different genetic methods. Some of the analyses we ran are:

- Estimating the proportion of the variance in age at autism diagnosis explained by the genetic variants studied. This is called genetic heritability or SNP heritability
- Looking at the shared genetics (called genetic correlation) between age at diagnosis and various mental health conditions. In other words, do the genetic variants that increase or decrease the age at autism diagnosis also increase or decrease the genetic propensity for various mental health conditions?
- Using a method called polygenic scores to create a summed index of the genetic likelihood for autism, ADHD, and mental health conditions and test their association with age at autism diagnosis.
- Running a statistical method called genetic structural equation model that looks at the relationship between different genetic studies to identify two correlated genetic factors linked to early vs later diagnosis.
- Analysing very rare genetic variants and their links to age at autism diagnosis.

What are the limitations of the study?
Some key limitations of this study are:

- The findings do not fully explain all of the variance in age at diagnosis, suggesting other contributing factors.

- We were unable to measure and study the effect of potentially non-genetic influences such as camouflaging/masking, stigma, healthcare access, waiting lists etc. on age at autism diagnosis.
- Analyses were limited by sample sizes for some datasets therefore we cannot assume that all our results apply to the entire population. For example, the number of autistic individuals in the long-term studies were around a hundred in each cohort.
- We observed some differences in the variance in age at autism diagnosis explained by the factors studied across cohorts. This suggests that there are cultural and geographic features across cohorts that impact these findings.
- We still do not fully understand what are the early developmental features that lead to a diagnosis of autism in late childhood/adolescence.
- Although we used the largest genetic datasets to date, it is possible that there may be more than two underlying genetic latent traits that contribute to when some of these features that lead to an autism diagnosis emerge, leading to differences in when an autism diagnosis is made.
- All datasets we used were from developed/western countries. It is unclear if we will find similar findings in countries from other parts of the world.

Although the study does not provide the full picture of the factors that contribute to age at autism diagnosis, it is an important first step towards understanding all the different factors that may impact when someone would receive an autism diagnosis.

Implications of the study

What is the impact of factors like camouflaging, waiting time, stigma on age at autism diagnosis?
The study did not directly measure the impact of factors like camouflaging autistic traits, delays in assessment/diagnosis waitlists, or stigma on age at autism diagnosis. However, we acknowledge that these likely play a role, in addition to the genetic, some demographic, and developmental factors that we have studied.

It is important to note that in this study, genetics explain only about 11% of the total variation in when someone receives an autism diagnosis. Similarly, developmental and some demographic factors explain between 10 - 60% of the variance in age at autism diagnosis across cohorts, with considerable variation among the cohorts studied. Taken together, it is clear that there are several other unmeasured factors that contribute to when someone receives an autism diagnosis.

More research is needed to understand how much these social/environmental variables influence the timing of when someone receives an autism diagnosis.

Are adolescent and childhood diagnosed autism two different types of autism?
No, our findings do not suggest that autism diagnosed in childhood versus adolescence are completely distinct conditions. Rather, the findings indicate there are at least two main genetic and developmental profiles. One of these profiles predispose some autistic individuals to showing clear traits from very early childhood leading to earlier diagnosis, while autistic individuals with the second profile may not

exhibit prominent characteristics until adolescence resulting in later diagnosis. However, the polygenic factors identified were moderately correlated.

I am/my child is potentially autistic, what are the implications of this study?
For individuals who may be autistic or have an autistic child, our findings underscore that there can be diversity in how and when autism traits manifest across development. Some key implications are:

- Closely monitoring a child's social/emotional development is important, as difficulties can potentially emerge later even if not apparent in early years.
- Consider getting an evaluation if difficulties arise - although some of these difficulties may be transient, they may be linked to other difficulties that emerge later on.
- Understanding a later autism diagnosis does not mean someone's autistic traits are any less valid.
- Different support approaches may be helpful depending on the child's unique profile and trajectory.

Can you use the findings from this study to diagnose/predict autism?
No, the findings from this study alone cannot be used to diagnose or definitively predict autism in individuals. The genetic scores and developmental trajectory patterns provide insights at a population level, but there is still substantial variability across individuals. Formal autism diagnostic evaluations by qualified professionals are still required.

What are some potential misinterpretations of the study?
Some potential misinterpretations of the study's findings include:

- Assuming all autistic children will follow one of just two rigid trajectories, when there is more variability.
- Families delaying seeking evaluation based on expectation that difficulties may naturally resolve.
- Oversimplifying the results to suggest there are two completely distinct "types" of autism.
- Overemphasising genetics while minimising environmental/social influences on age at autism diagnosis.
- Trying to use genetic analyses to self-diagnose or make predictions at an individual level.
- Assuming that autistic individuals can be subgrouped based on when they were diagnosed. Age at diagnosis is merely a loose index of different emergence of developmental trajectories. Several other factors can influence the age of diagnosis in a person.

Glossary of terms

Trajectories: Trajectories are the different paths or ways that things can develop over time. In this study, we found two different paths for how autistic children's behaviours and social skills changed as they got older.

Genetic variant: A genetic variant is a difference in someone's genes or DNA code compared to other people. These small variations can sometimes affect things like a person's traits or health.

GWAS: This is a way for scientists to look at all of a person's genes to try and find which genetic variants are linked to certain traits or conditions, like autism.

Polygenic score: This is a number that shows how many genetic variants linked to a trait, like autism, a person has. A higher number means more of those variants.

*De novo varian*t: These are new genetic variants that weren't inherited from the person's parents, but appeared newly in a person’s DNA.

Genetically inferred ancestry: This refers to using a person's genes to figure out what broad geographic regions their ancestors likely came from a long time ago.

Latent trait: This is an unseen characteristic that cannot be directly measured but can be calculated using other correlated measured characters.

Supplementary Figure 1: Conduct problems

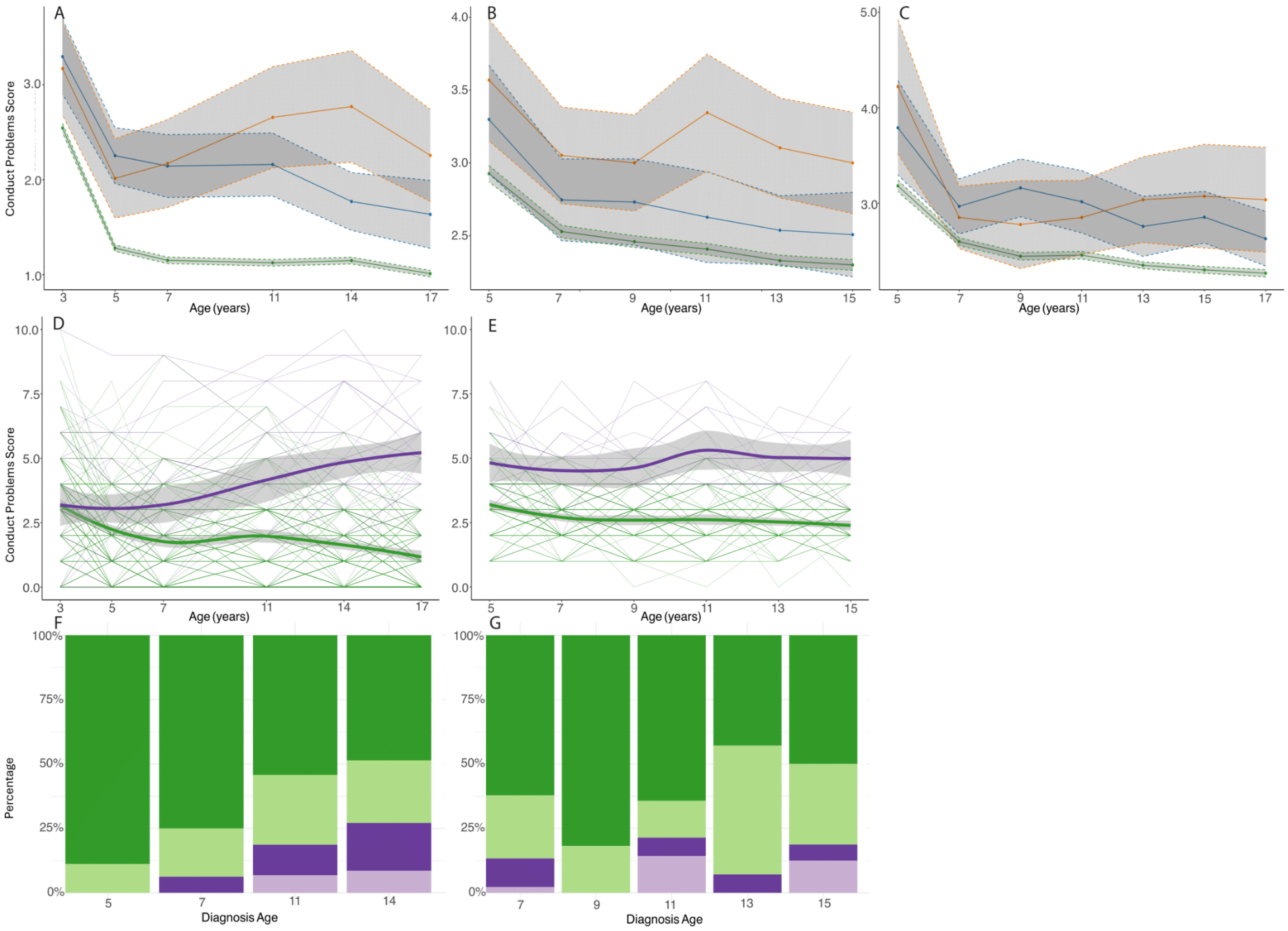

A - C: Mean total conduct problem scores in autistic individuals diagnosed in childhood (blue) and adolescence (orange), and individuals without an autism diagnosis (green) in the MCS-C (A), LSAC-B (B), and LSAC-K (C) cohorts. Grey regions indicate 95% confidence intervals. D - E: Longitudinal growth mixture models of total conduct problem scores among autistic individuals, demonstrating the presence of two groups (green indicating early childhood emergent latent class and purple indicating late childhood emergent latent class) in the MCS-C (D), LSAC-B (E). Only a single latent group was identified in LSAC-K and hence not plotted. F-G: Stacked bar charts providing proportion of individuals who had been diagnosed as autistic at specific ages by the latent classes membership from the growth mixture models in MCS-C (F) and LSAC-B (G).

Supplementary Figure 2: Emotional symptoms

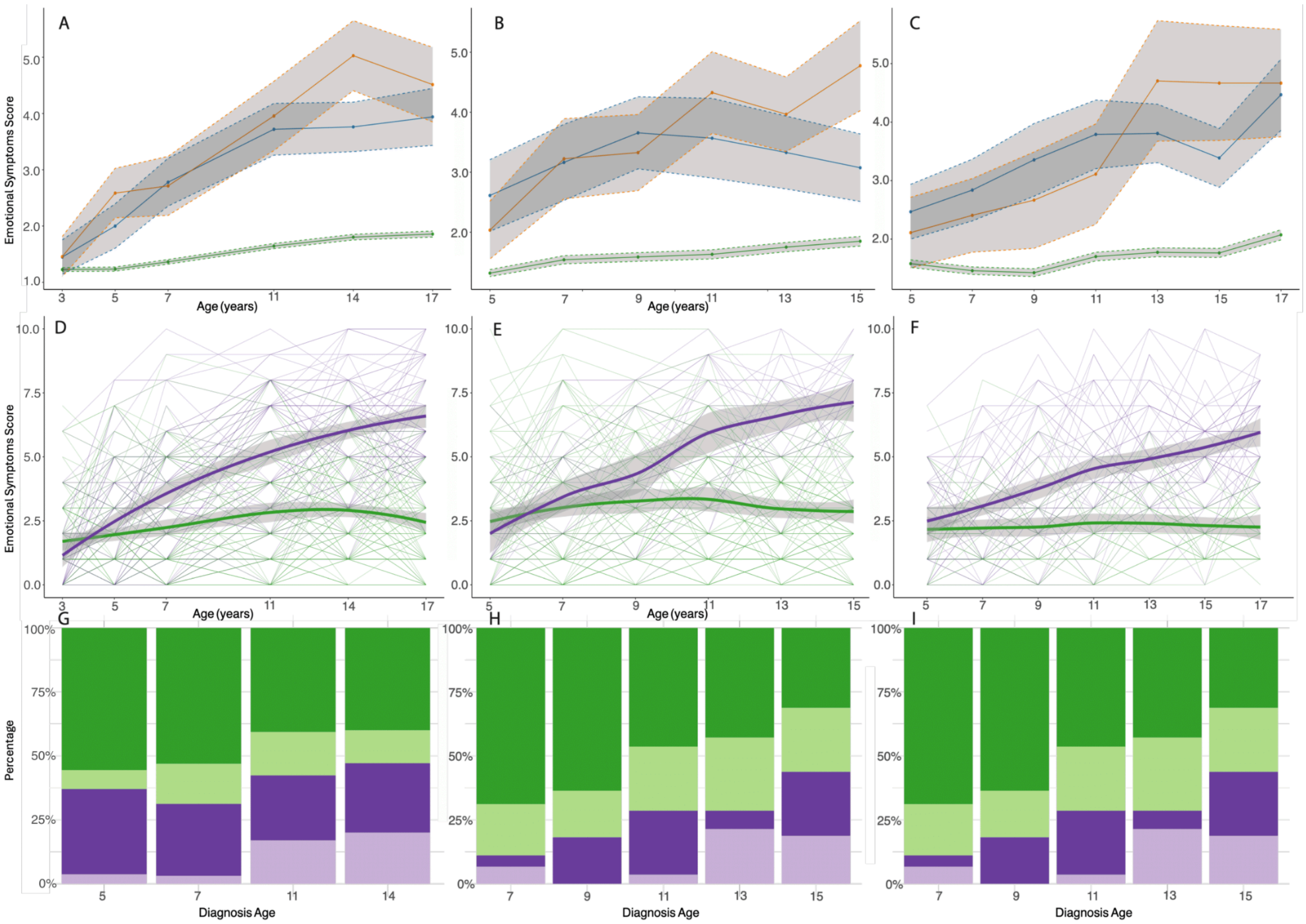

A - C: Mean total emotional symptom scores in autistic individuals diagnosed in childhood (blue) and adolescence (orange), and individuals without an autism diagnosis (green) in the MCS (A), LSAC-B (B), and LSAC-K (C) cohorts. Grey regions indicate 95% confidence intervals. D - F: Longitudinal growth mixture models of total emotional symptoms scores among autistic individuals, demonstrating the presence of two groups (green indicating early childhood emergent latent class and purple indicating late childhood emergent latent class) in the MCS (D), LSAC-B (E) and LSAC-K (F) cohorts. G - J: Stacked bar charts providing proportion of individuals who had been diagnosed as autistic at specific ages by the latent classes membership from the growth mixture models in MCS (G), LSAC-B (H), and LSAC-K (I) cohorts. Darker colours indicate males and lighter colours indicate females.

Supplementary Figure 3: Hyperactivity/Inattention

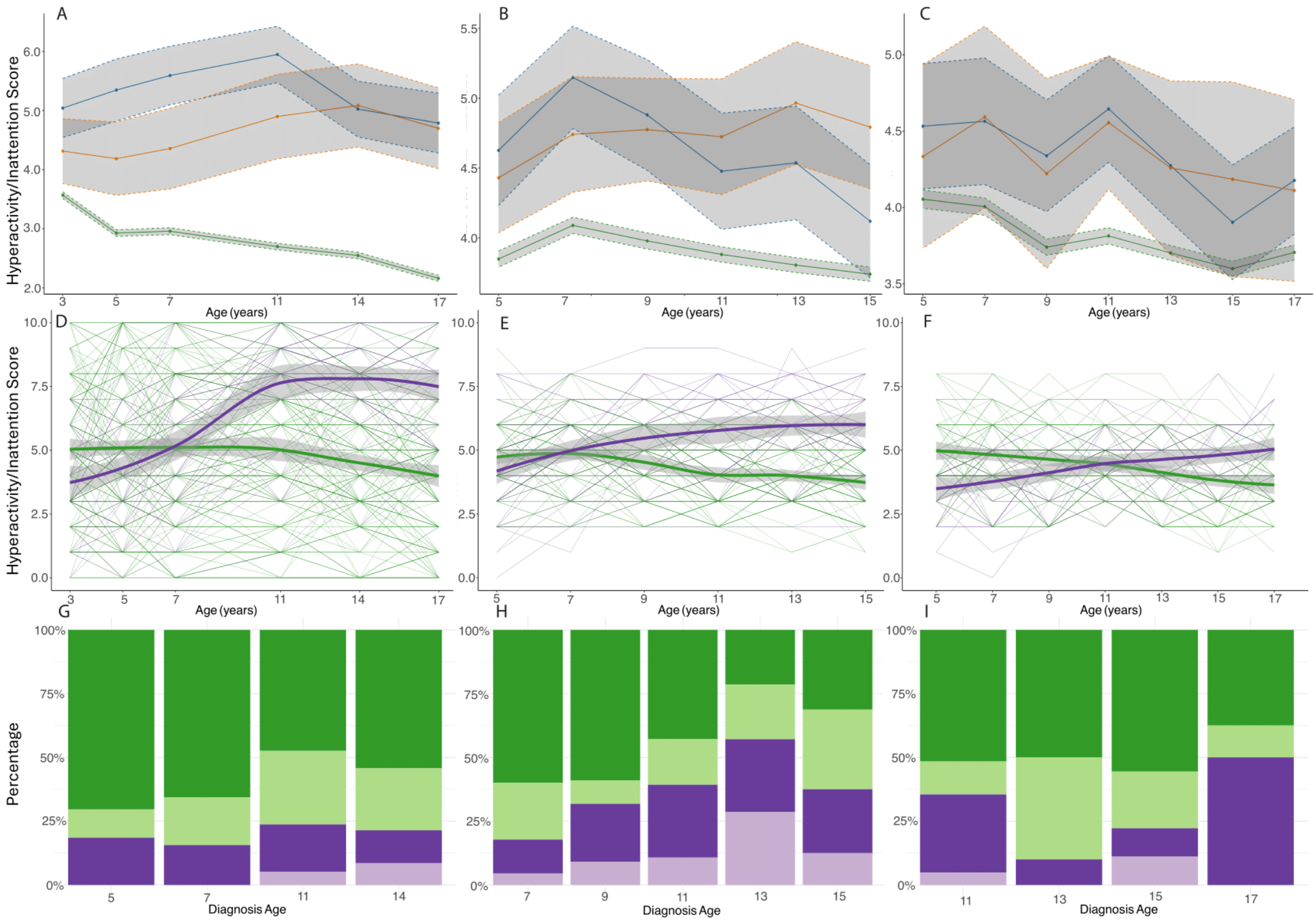

A - C: Mean total emotional symptom scores in autistic individuals diagnosed in childhood (blue) and adolescence (orange), and individuals without an autism diagnosis (green) in the MCS (A), LSAC-B (B), and LSAC-K (C) cohorts. Grey regions indicate 95% confidence intervals. D - F: Longitudinal growth mixture models of total emotional symptoms scores among autistic individuals, demonstrating the presence of two groups (green indicating early childhood emergent latent class and purple indicating late childhood emergent latent class) in the MCS (D), LSAC-B (E) and LSAC-K (F) cohorts. G - J: Stacked bar charts providing proportion of individuals who had been diagnosed as autistic at specific ages by the latent classes membership from the growth mixture models in MCS (G), LSAC-B (H), and LSAC-K (I) cohorts. Darker colours indicate males and lighter colours indicate females.

Supplementary Figure 4: Peer relationship problems

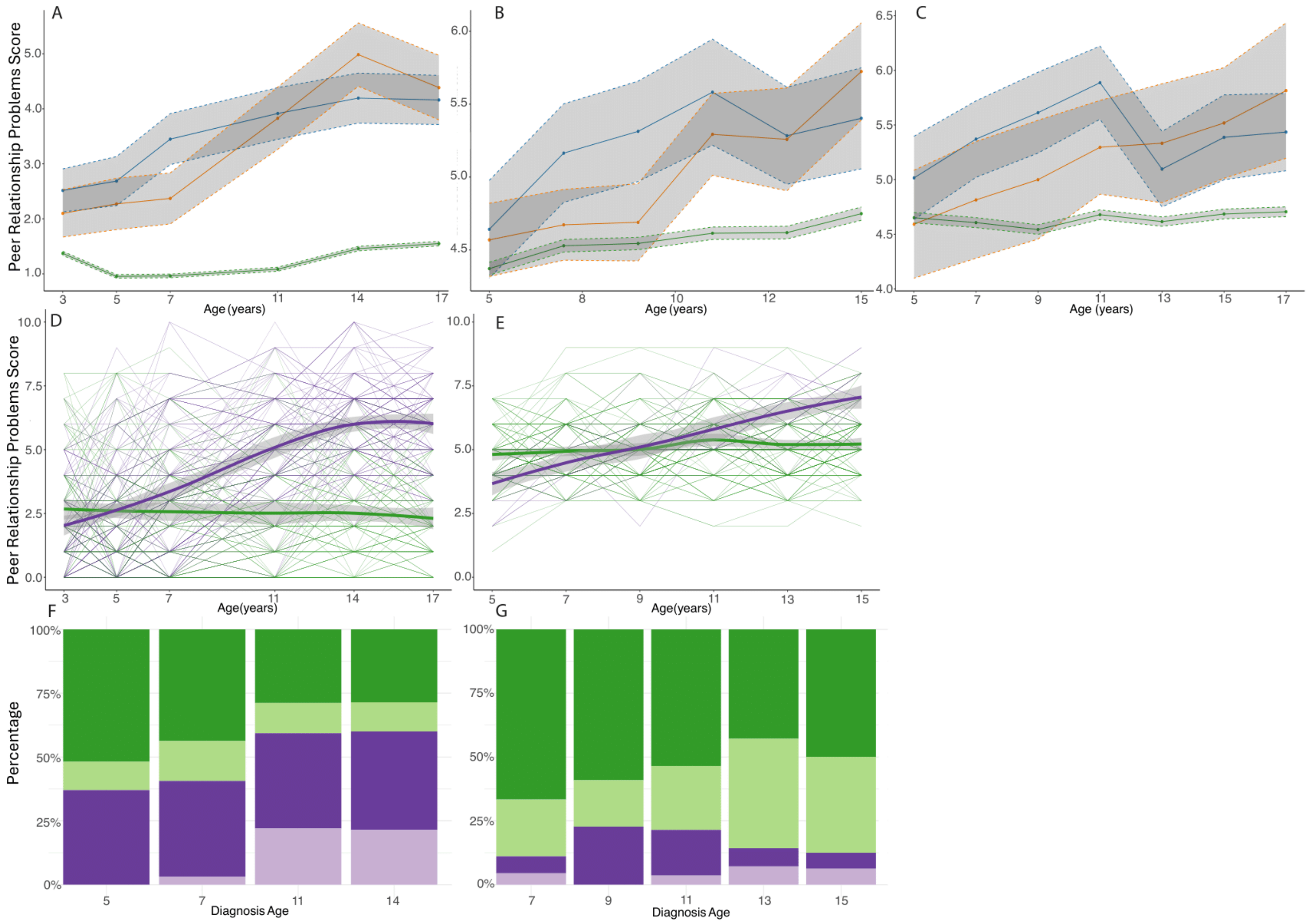

A - C: Mean peer relationship scores in autistic individuals diagnosed in childhood (blue) and adolescence (orange), and individuals without an autism diagnosis (green) in the MCS-C (A), LSAC-B (B), and LSAC-K (C) cohorts. Grey regions indicate 95% confidence intervals. D - E: Longitudinal growth mixture models of peer relationship scores among autistic individuals, demonstrating the presence of two groups (green indicating early childhood emergent latent class and purple indicating late childhood emergent latent class) in the MCS-C (D), LSAC-B (E). Only a single latent group was identified in LSAC-K and hence not plotted. F-G: Stacked bar charts providing proportion of individuals who had been diagnosed as autistic at specific ages by the latent classes membership from the growth mixture models in MCS-C (F) and LSAC-B (G).

Supplementary Figure 5: Prosocial behaviour

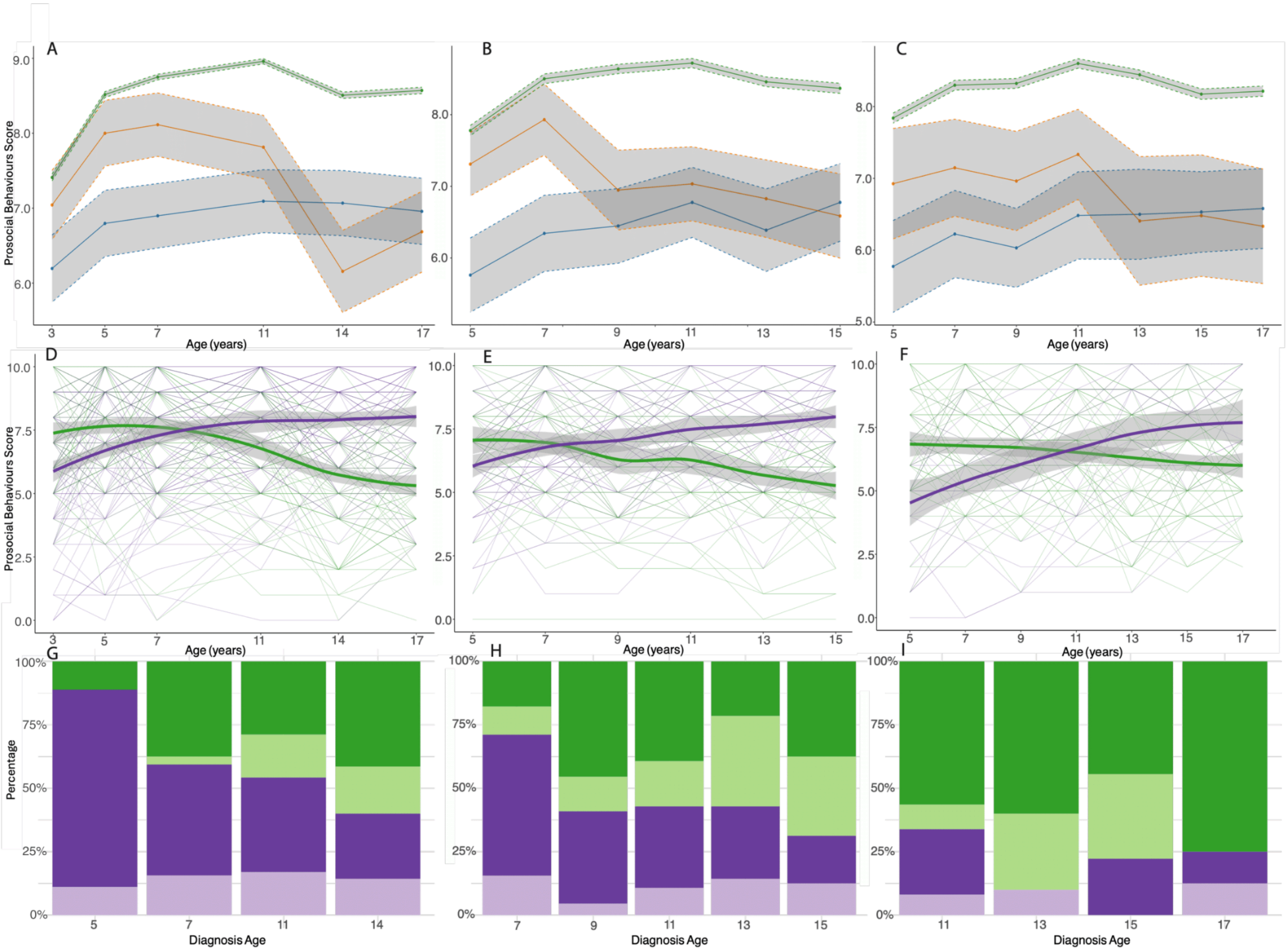

A - C: Mean total prosocial behaviour scores in autistic individuals diagnosed in childhood (blue) and adolescence (orange), and individuals without an autism diagnosis (green) in the MCS (A), LSAC-B (B), and LSAC-K (C) cohorts. Grey regions indicate 95% confidence intervals. D - F: Longitudinal growth mixture models of total prosocial behaviour scores among autistic individuals, demonstrating the presence of two groups (green indicating early childhood emergent latent class and purple indicating late childhood emergent latent class) in the MCS (D), LSAC-B (E) and LSAC-K (F) cohorts. G - J: Stacked bar charts providing proportion of individuals who had been diagnosed as autistic at specific ages by the latent classes membership from the growth mixture models in MCS (G), LSAC-B (H), and LSAC-K (I) cohorts. Darker colours indicate males and lighter colours indicate females.

Supplementary Figure 6: Results from GUI

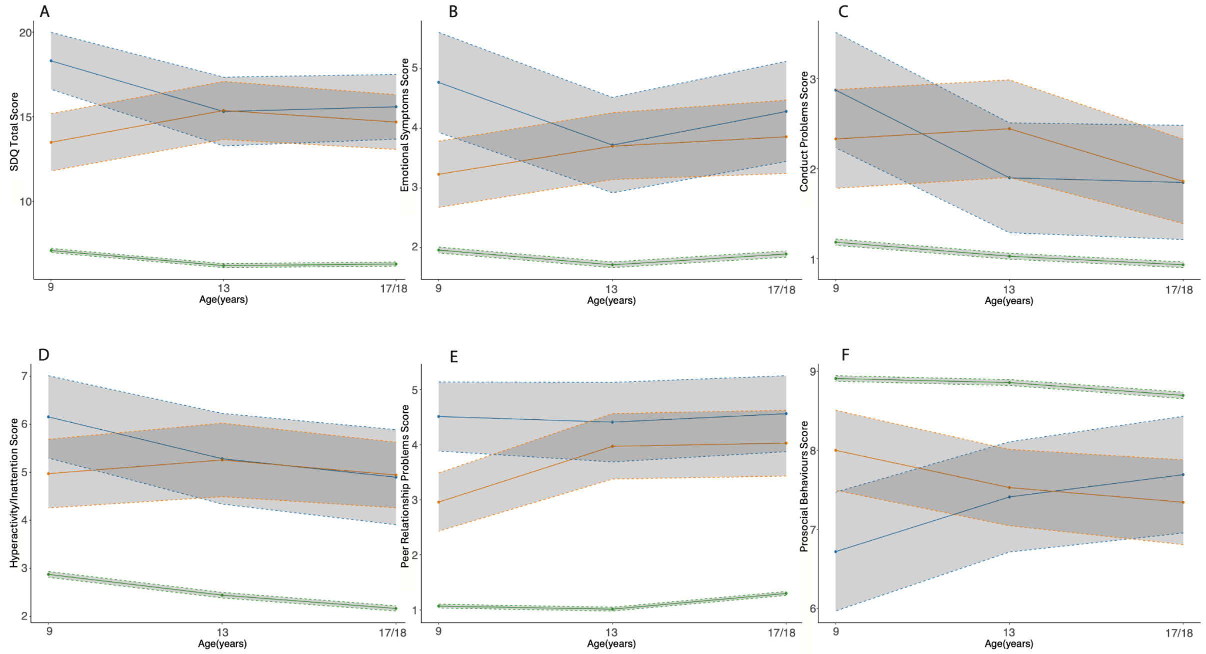

Mean SDQ and subscale scores in autistic individuals diagnosed in childhood (blue) and adolescence (orange), and individuals without an autism diagnosis (green) in the GUI cohorts. (A) SDQ total scores; (B) Emotional symptoms scores; (C) Conduct problem scores; (D) Hyperactivity/inattention scores; (E) Peer problem scores; (F) Prosocial behaviour scores.

Supplementary Figure 7: Sensitivity analyses for ADHD

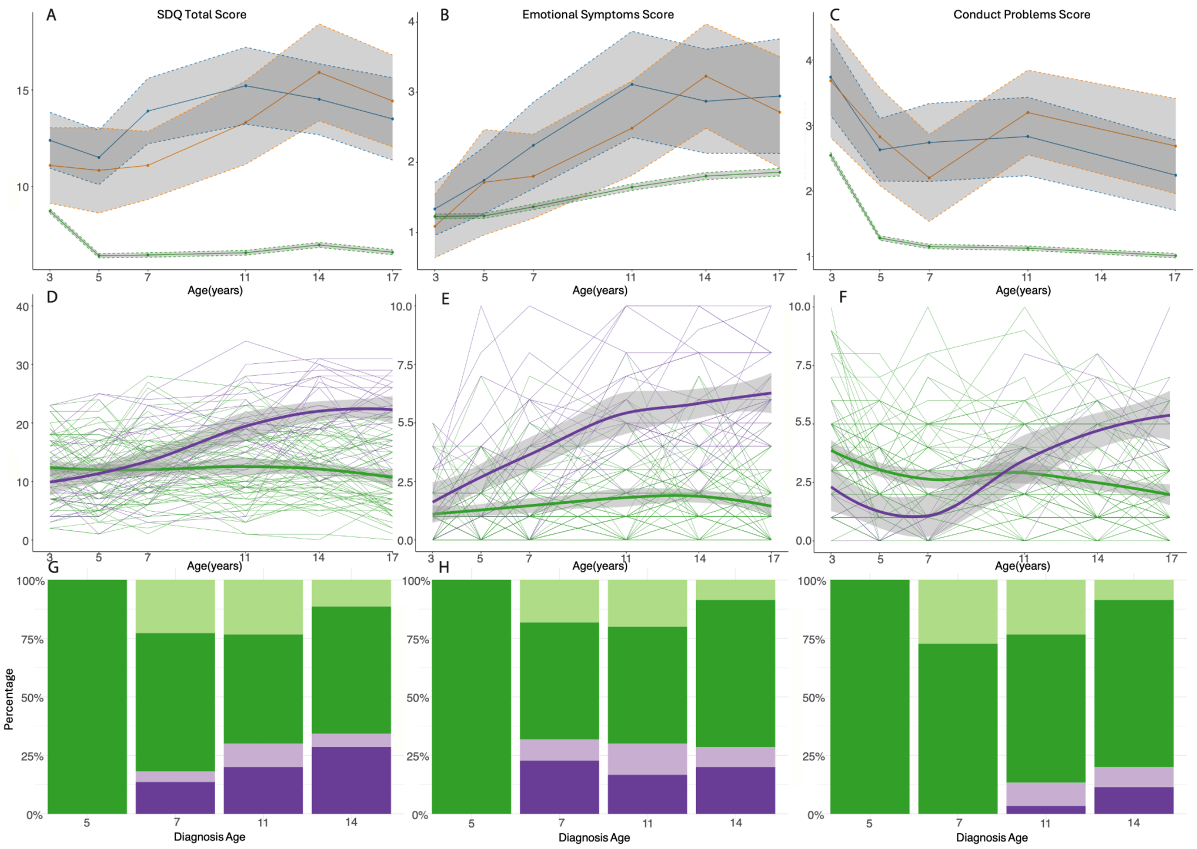

A - C: Mean total SDQ (A), emotional symptoms (B), and conduct problem (C) scores in individuals diagnosed with ADHD in childhood (blue) and adolescence (orange), and individuals without an ADHD diagnosis (green) in the MCS cohort. Grey regions indicate 95% confidence intervals. D - F: Longitudinal growth mixture models of total SDQ (D), emotional symptoms (E), and conduct problem (F) scores among individuals with an ADHD diagnosis, demonstrating the presence of two groups (green indicating early childhood emergent latent class and purple indicating late childhood emergent latent class) in the MCS. G - J: Stacked bar charts providing proportion of individuals who had been diagnosed with ADHD at specific ages by the latent classes membership from the growth mixture models of total SDQ (G), emotional symptoms (H) and conduct problem (J) scores in the MCS. Darker colours indicate males and lighter colours indicate females.

Supplementary Figure 8: Sensitivity analyses for ADHD (cont.)

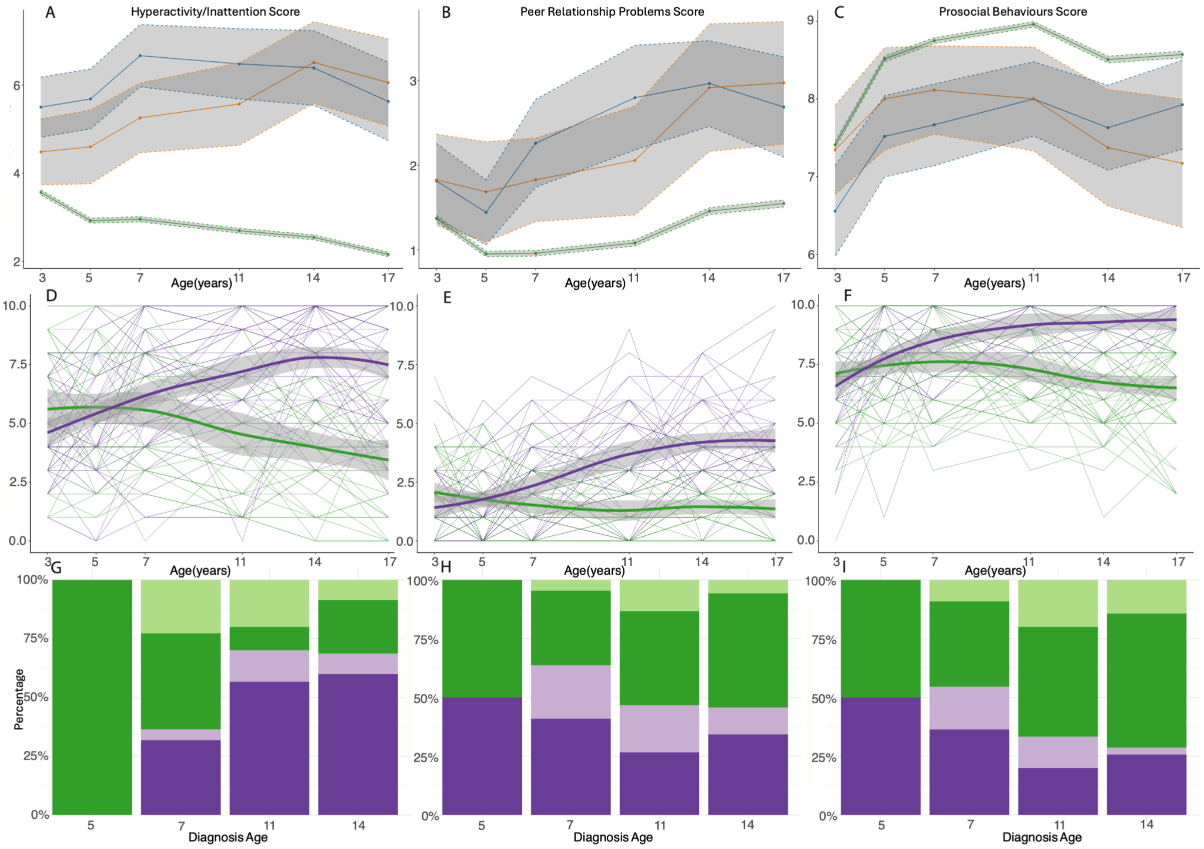

A - C: Mean hyperactivity/inattention (A), peer relationship problems (B), and prosocial behaviours (C) scores in individuals diagnosed with ADHD in childhood (blue) and adolescence (orange), and individuals without an ADHD diagnosis (green) in the MCS cohort. Grey regions indicate 95% confidence intervals. D - F: Longitudinal growth mixture models of hyperactivity/inattention (D), peer problems (E), and prosocial behaviour (F) scores among individuals with an ADHD diagnosis, demonstrating the presence of two groups (green indicating early childhood emergent latent class and purple indicating late childhood emergent latent class) in the MCS. G - J: Stacked bar charts providing proportion of individuals who had been diagnosed with ADHD at specific ages by the latent classes membership from the growth mixture models of hyperactivity/inattention (G), peer problems (H) and prosocial behaviour (J) scores in the MCS. Darker colours indicate males and lighter colours indicate females.

Supplementary Figure 9: Genetic correlation between age at autism diagnosis and autism GWAS ordered by sex and median age at diagnosis.

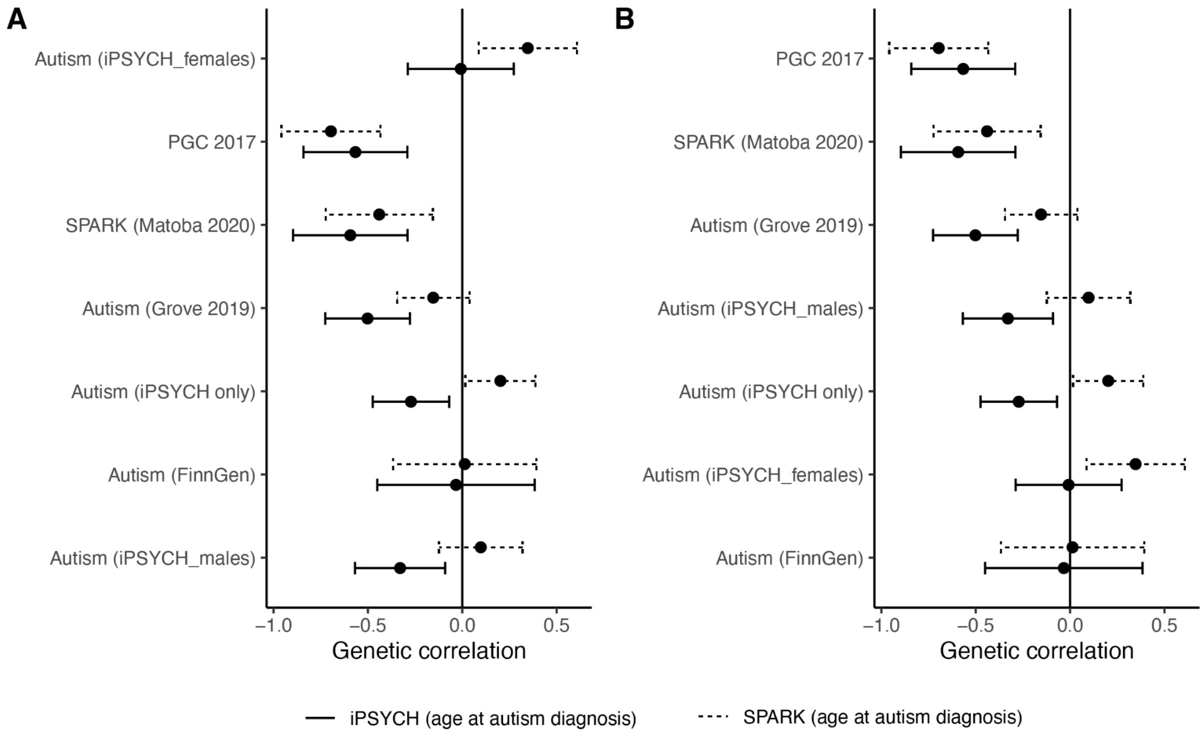

Genetic correlation between age at autism diagnosis and autism GWAS. A. GWAS have been ordered by the proportion of autistic female individuals ranging from 1 (iPSYCH_females) to 0 (iPSYCH_males). B. GWAS have been ordered by the approximate median age at autism diagnosis ranging from early twenties (FinnGen) to under 4 years of age (SPARK and PGC 2017).

Supplementary Figure 10: GenomicSEM models with alternate GWAS

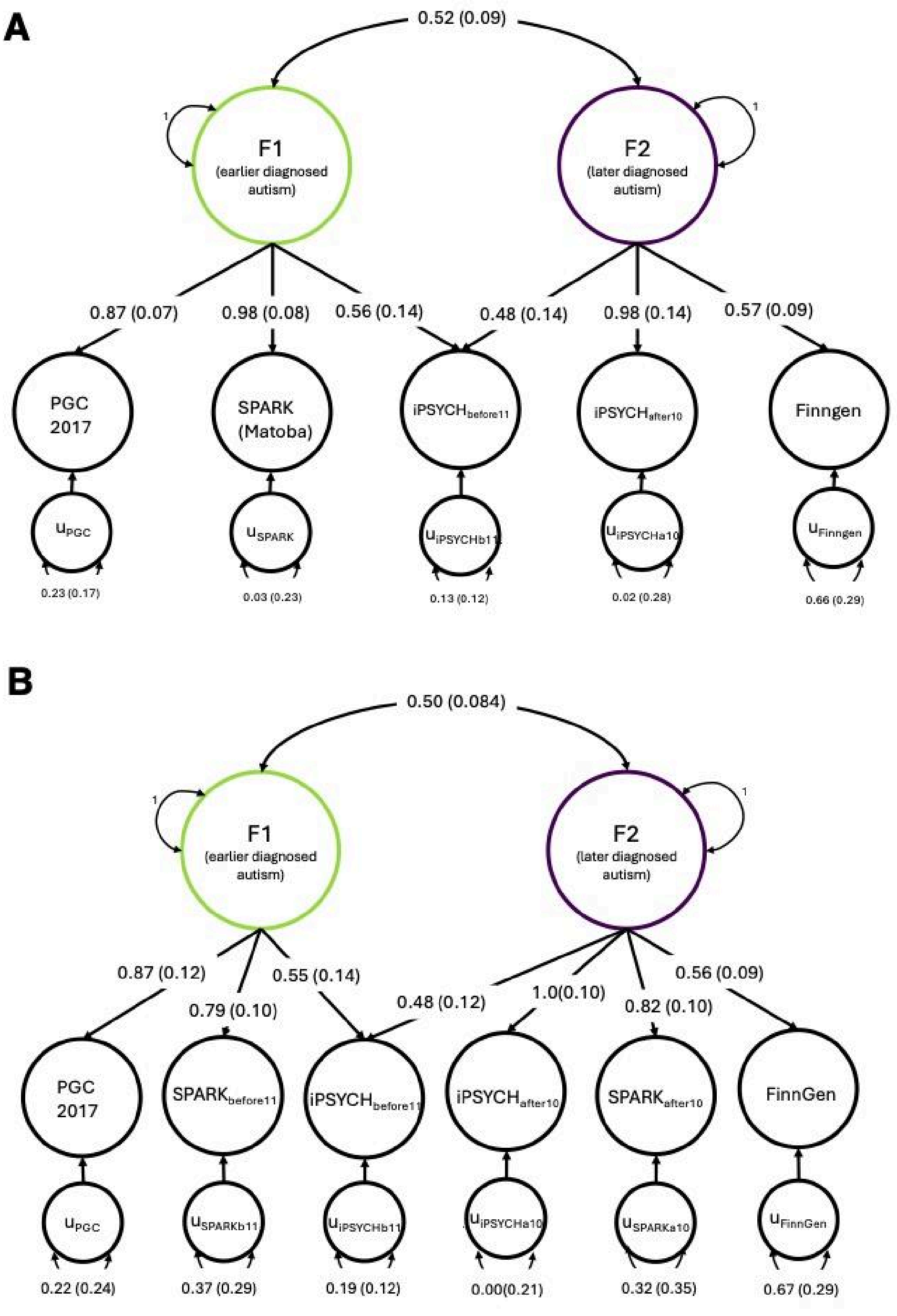

A. Path diagram illustrating the two correlated genetic factor models for autism, using five (A) and six (B) minimally overlapping autism GWAS datasets. F1 = Factor 1, F2 = Factor 2. One-headed arrows depict the regression relationship pointing from the independent variables to the dependent variables. The numbers are the regression coefficients of the factor loadings, with the standard errors provided in parenthesis. Covariance between variables are represented as two-headed arrows linking the variables. The numbers on the two-headed arrows can be interpreted as genetic correlation estimates with the standard errors provided in parenthesis. Residual variances are represented using a two-headed arrow connecting the variable to itself. Standard errors are provided in parenthesis.

References

1. United Kingdom. Parliament. Human Tissue Act 2004. (2004) doi:10.5040/9781474200592.ch-004.
2. softImpute: Matrix Completion via Iterative Soft-Thresholded SVD. *Comprehensive R Archive Network (CRAN)* https://cran.r-project.org/web/packages/softImpute/index.html.
3. SPARK Consortium. Electronic address: pfeliciano@simonsfoundation.org & SPARK Consortium. SPARK: A US Cohort of 50,000 Families to Accelerate Autism Research. *Neuron* **97**, 488–493 (2018).
4. Bybjerg-Grauholm, J. *et al.* The iPSYCH2015 Case-Cohort sample: updated directions for unravelling genetic and environmental architectures of severe mental disorders. Preprint at https://doi.org/10.1101/2020.11.30.20237768.
5. Fombonne, E. Autism in adult life. *Can. J. Psychiatry* **57**, 273–274 (2012).
6. The Autism Spectrum Disorders Working Group of The Psychiatric Genomics Consortium. Meta-analysis of GWAS of over 16,000 individuals with autism spectrum disorder highlights a novel locus at 10q24.32 and a significant overlap with schizophrenia. *Mol. Autism* **8**, 21 (2017).
7. Lord, C. *et al.* The autism diagnostic observation schedule-generic: a standard measure of social and communication deficits associated with the spectrum of autism. *J. Autism Dev. Disord.* **30**, 205–223 (2000).
8. Lord, C. *et al.* Autism Diagnostic Interview-Revised: A revised version of a diagnostic interview for caregivers of individuals with possible pervasive developmental disorders. *J. Autism Dev. Disord.* **24**, 659–685 (1994).
9. Demontis, D. *et al.* Genome-wide analyses of ADHD identify 27 risk loci, refine the genetic architecture and implicate several cognitive domains. *Nat. Genet.* **55**, 198–208 (2023).
10. Als, T. D. *et al.* Depression pathophysiology, risk prediction of recurrence and comorbid psychiatric disorders using genome-wide analyses. *Nat. Med.* **29**, 1832–1844 (2023).
11. Trubetskoy, V. *et al.* Mapping genomic loci implicates genes and synaptic biology in schizophrenia. *Nature* **604**, 502–508 (2022).

12. Mullins, N. *et al.* Genome-wide association study of more than 40,000 bipolar disorder cases provides new insights into the underlying biology. *Nat. Genet.* **53**, 817–829 (2021).
13. Watson, H. J. *et al.* Genome-wide association study identifies eight risk loci and implicates metabo-psychiatric origins for anorexia nervosa. *Nat. Genet.* **51**, 1207–1214 (2019).
14. Nievergelt, C. M. *et al.* Genome-wide association analyses identify 95 risk loci and provide insights into the neurobiology of post-traumatic stress disorder. *Nat. Genet.* **56**, 792–808 (2024).
15. Terhaag, S., Fitzsimons, E., Daraganova, G. & Patalay, P. Sex, ethnic and socioeconomic inequalities and trajectories in child and adolescent mental health in Australia and the UK: findings from national prospective longitudinal studies. *J. Child Psychol. Psychiatry* **62**, 1255–1267 (2021).
16. Hayes, A. F. *Introduction to Mediation, Moderation, and Conditional Process Analysis: A Regression-Based Approach*. (Guilford Publications, 2021).
